# PU.1 is a mediator of the reactive stromal response associated with castration-resistant prostate cancer

**DOI:** 10.1038/s42003-026-10297-7

**Published:** 2026-05-29

**Authors:** Francesco Bonollo, Daniel Hanhart, Simone de Brot, Wanli Cheng, Panagiotis Chouvardas, Beat Roth, George N. Thalmann, Natalie Sampson, Marianna Kruithof-de Julio, Sofia Karkampouna

**Affiliations:** 1 Urology Research Laboratory, Department for BioMedical Research, University of Bern, Bern, Switzerland; 2 COMPATH, Institute of Animal Pathology, University of Bern, Bern, Switzerland; 3 Department of Urology, Inselspital, Bern University Hospital, University of Bern, Bern, Switzerland; 4 Department of Urology, Medical University of Innsbruck, Innsbruck, Austria; 5 Institute of Pathophysiology, Medical University of Innsbruck, Innsbruck, Austria

**Keywords:** Prostate cancer, Cancer microenvironment

## Abstract

Emerging evidence demonstrates the pivotal role played by the tumor microenvironment, particularly cancer-associated fibroblasts, during the development of castration-resistant prostate cancer. In this study, the molecular composition of the tumor microenvironment of androgen-sensitive and castration-resistant metastatic prostate cancer is investigated by utilizing patient-derived xenograft models. Transcriptomic and histological analysis identify the presence of a pro-fibrotic stroma in the castration-resistant (LAPC9) versus the castration-sensitive (PNPCa, BM18) models, characterized by high levels of collagen and tenascin C deposition and upregulation of inflammatory markers. Intra-tumoral collagen- and tenascin-positive stromal areas specifically correlate with higher tumor invasiveness. Master regulator analysis identifies the transcription factor PU.1 as a mediator of the LAPC9 pro-fibrotic phenotype, whose transcriptional activity can be specifically inhibited by the small molecule DB1976. To test the effect of pharmacologic PU.1 inhibition a novel organoid-fibroblast 3D co-culture system, able to mimic features of the in vivo tumor microenvironment, is established. Inhibition of stromal PU.1 activity reverts the pro-fibrotic phenotype and, in turn, reduces tumor organoid growth. Besides identifying a novel molecular player of the pro-fibrotic stromal phenotype in prostate cancer, this study highlights the applicability of 3D tumor organoid-fibroblast co-cultures as in vitro tools to test the effect of stromal-targeting compounds.

## Abstract (web summary)

Pro-fibrotic and pro-inflammatory tumor microenvironment is a typical feature of castration-resistant advanced prostate cancer. Inhibition of identified master regulator PU.1, reverts the profibrotic phenotype and in turn tumor cell proliferation.

## Introduction

Prostate cancer (PCa) is one of the most diagnosed cancers in men, with the second-highest incidence in the male population worldwide^1^. Androgen deprivation therapy (ADT) is usually prescribed for patients who experience relapse after primary treatment (radical prostatectomy) since the growth of this tumor type is, at least in the initial stages of disease, dependent on androgen receptor (AR) signaling^2^. Despite improved survival upon second-line ADT (e.g., abiraterone acetate, enzalutamide^3–5^), resistance eventually develops, leading to progression towards castration-resistant prostate cancer (CRPC), and in turn to metastatic progression, whereby bone metastasis accounts for up to 90% of PCa-related deaths^2,6,7^.

An increasing number of studies have demonstrated the crucial role played by the tumor microenvironment (TME) during PCa progression^8,9^. The prostate TME is mainly composed of cancer-associated fibroblasts (CAFs), smooth muscle cells, endothelial cells, pericytes, immune cells, and extracellular matrix (ECM) proteins (e.g., collagen, fibronectin). CAFs interact with PCa cells through several mechanisms, such as secretion of signaling molecules and growth factors (paracrine communication), direct cell-cell contact, and ECM remodeling^8^. Compared to normal prostate tissue, the prostate TME, also referred to as “reactive stroma,” displays a higher number of fibroblasts and a lower number of smooth muscle cells^10^. In addition, fibroblasts in the TME acquire a contractile “myofibroblast (MFB)-like” phenotype, induced by increased TGFβ-signaling activity, characterized by the co-expression of the mesenchymal marker vimentin and alpha-smooth muscle actin (α-SMA)^10^. MFB-like CAFs increase collagen deposition and secrete the ECM protein Tenascin C (TNC), which is not observed in the normal prostate microenvironment^10^. TNC is a hexameric glycoprotein that acts as a reservoir of growth factors and binds other ECM proteins and cellular receptors (integrins), modulating intracellular signaling activity^11^. It is normally expressed during embryonic development or during wound healing, but is re-expressed in many cancer types, such as prostate or breast, and plays an important role in mediating immune evasion and formation of bone metastasis^12–14^. Recent studies have highlighted how CAFs not only display MFB-like features but rather represent a heterogeneous cell population that can express multiple markers such as α-SMA, platelet-derived growth factor receptor -β (PDGFR-β), fibroblast activation protein (FAP), and fibroblast-specific protein-1 (FSP-1, also known as S100a4), playing both tumor-supporting and tumor-suppressive functions in the TME^15,16^. The advent of single-cell RNA sequencing (scRNA-seq) has shown that CAFs display distinct gene expression profiles within and across different solid cancer types (pancreatic, breast, lung, ovarian, and prostate)^17–21^. Three main CAF subtypes are currently largely recognized, namely myofibroblast-like CAFs (myCAFs), inflammatory CAFs (iCAFs), and antigen-presenting CAFs (apCAFs)^22,23^. myCAFs represent the CAF subtype with the closest features to MFBs, displaying a contractile phenotype with high α-SMA expression, inducing ECM remodeling, and responding to TGF-β signaling. iCAFs secrete pro-inflammatory cytokines such as interleukin-6 (IL-6) and IL-1 and modulate the recruitment and activity of immune cells. apCAFs express major histocompatibility complex class-II (MHC-II) molecules (similarly to dendritic cells) needed for antigen presentation. They also modulate the immune system activity, and they can have both tumor-promoting and tumor-protective roles^23^. Recent studies have identified specific CAF subpopulations in PCa that contribute to ADT resistance of tumor cells through distinct molecular mechanisms, such as neuregulin-1 (NRG1)-positive CAFs, chemokine (C-C motif) ligand 5 (CCL5)-secreting CAFs, and secreted phosphoprotein 1-positive (SPP1+) CAFs^24–26^.

Patient-derived xenograft (PDX) models have proven to be useful tools for studying the TME of advanced PCa, particularly in bone metastasis^27,28^. By exploiting the different species of origin of tumor (human) and stromal (mouse) cells, it is possible to discriminate tumor- versus stromal-specific gene expression profiles. Indeed, our previous analysis^28^ of stromal profiling of different PCa disease stages modeled by PDXs, i.e., androgen-sensitive (BM18)^29^ and castration-resistant (LAPC9)^30^ bone metastasis, indicated distinct stromal transcriptomic changes not only among different stages but also upon hormonal modulation (castration). In particular, enrichment of ECM, cell adhesion, and cell migration in the LAPC9 stroma suggested that the stroma of the castration-resistant model displays a fibrotic myCAF-like phenotype.

One of the challenges in CAF/TME research is the limited use of in vitro models that accurately recapitulate tumor-stromal interactions occurring in the in vivo setting^31^. Patient- and PDX-derived organoids preserve histopathological, genomic, and transcriptomic features of the parental tumor and thus represent suitable tools for drug testing^27,32,33^. These models, however, lack key stromal components such as CAFs, endothelial cells, and immune cells, and thus do not permit assessment of the TME contribution to drug response. In contrast, ex vivo tissue slices and indirect/direct tumor-CAF 3D co-culture models represent amenable platforms to study TME changes occurring during tumorigenesis and upon drug treatment^31^. Whilst few studies to date have focused on their application for PCa^24,34–37^, matrigel-based prostate organoid 3D cultures demonstrated improved organoid formation and increased organoid branching upon the addition of prostate-derived fibroblasts, similar to the branched prostate acinar structures observed in vivo. Such studies suggest that 3D co-cultures induce the formation of a microenvironment that may better resemble the in vivo tumor tissue^37^.

Given the multiple and complex roles played by the TME during PCa tumorigenesis, we sought to characterize stromal pro-tumorigenic features that are associated with aggressive CRPC with the goal of identifying targetable genes to revert this pro-tumorigenic phenotype. Using androgen-sensitive (PNPCa^27^, BM18^29^) and castration-resistant (LAPC9^30^) subcutaneous PDX models of advanced PCa, we delineated the TME composition at the molecular level across distinct PCa stages and identified the transcription factor PU.1 as a CAF-specific master regulator (MR) that drives the pro-fibrotic stromal phenotype, whose targeting reverted the CAF-associated MFB phenotype and reduced tumor organoid growth, implying its translational potential to inhibit tumorigenesis of advanced PCa. The in vitro collagen-based 3D tumor-CAF organoid co-culture system established herein recapitulates, at least partially, diverse features of the in vivo TME, underscoring the value of this model for the discovery of CAF-targeting compounds.

## Results

### A myofibroblast- and extracellular matrix-rich stroma distinguishes the castration-resistant LAPC9 model from androgen-sensitive models

**Fig. 1:**
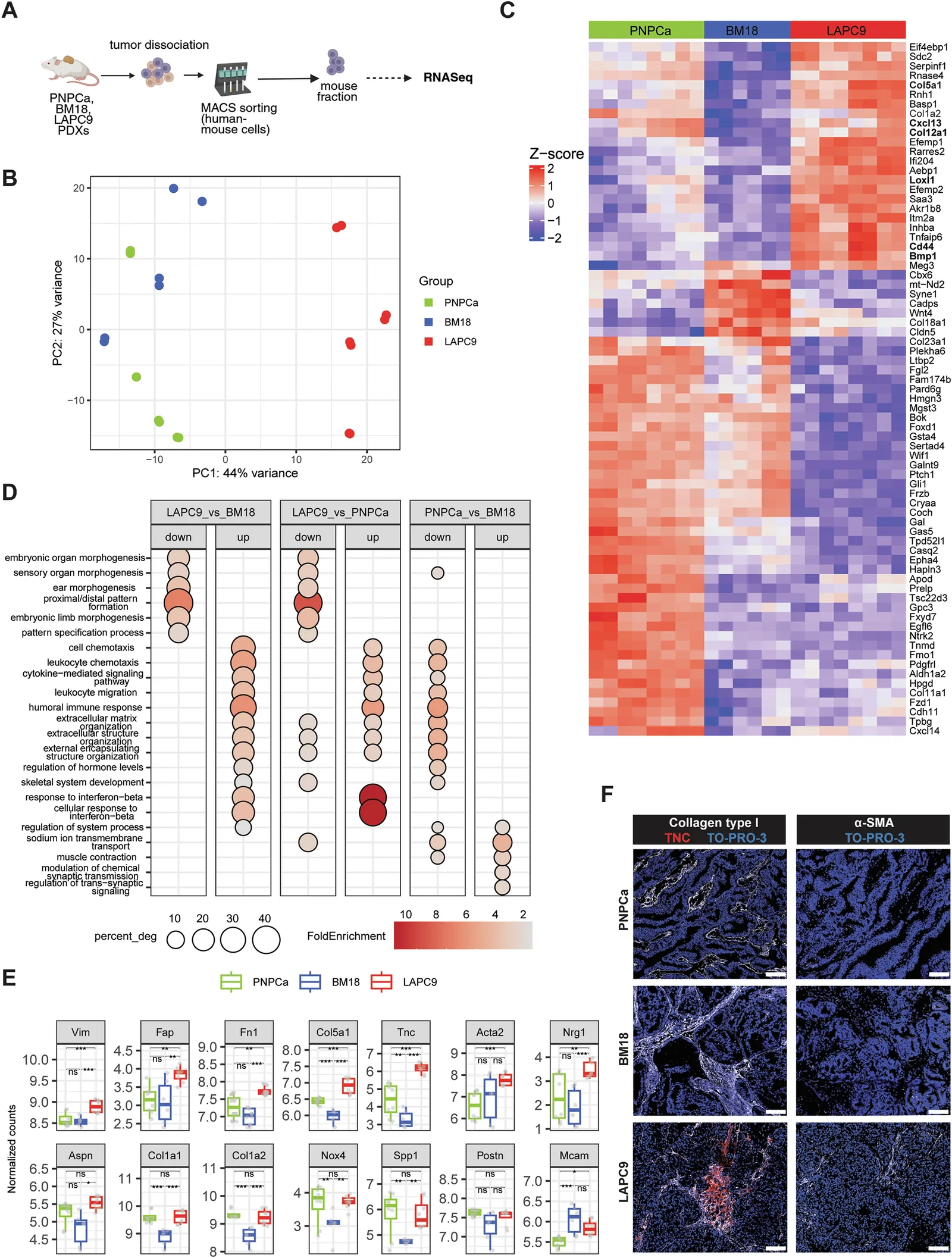
The LAPC9 castration-resistant tumor model displays highly fibrotic, myofibroblast-rich stromal features compared to androgen-sensitive tumors. **A** Schematic overview of stromal-specific RNA-sequencing of patient-derived xenograft (PDX) models. The PDX mouse fraction was sequenced following tumor tissue digestion and magnetic-associated cell sorting (MACS) separation of the tumor (human) and stromal (mouse) fractions at passage 0 (p0). Created in BioRender. Karkampouna, S. (2026) https://BioRender.com/z83i59b. **B** Principal component analysis (PCA) of the gene expression profile of PNPCa, BM18, and LAPC9 stromal (mouse) digested single cells (p0 condition). **C** Heatmap of the top 30 differentially expressed genes (DEGs) ranked by *p*-value from each comparison in the mouse fraction p0 conditions: PNPCa vs. BM18, LAPC9 vs. BM18, and PNPCa vs. LAPC9. Variance-stabilized counts were row-wise Z-scored for each gene. Extracellular matrix (ECM)-related, ECM-remodeling, and pro-inflammatory genes are highlighted in bold. **D** Enrichment analysis of the LAPC9 vs. BM18 and LAPC9 vs. PNPCa comparisons in the mouse fraction p0 (p0 ms) conditions performed with clusterProfiler. Circle size indicates the percentage of genes differentially expressed in a certain gene set, and the color scale indicates the adjusted *p*-value. **E** Boxplots of the expression levels (log1p of median of ratios normalized counts) of the reported mouse genes in PNPCa, BM18, and LAPC9 stromal cells (p0) based on the transcriptomic analysis. Statistical significance was calculated via the Wald test with Benjamini–Hochberg multiple test correction, *p* < 0.05 (*), *p* < 0.01 (**), *p* < 0.001(***). **F** Immunofluorescent staining of formalin-fixed paraffin-embedded (FFPE) tissue sections from the indicated PDX model. Left panels: collagen type I (blue channel, represented in white) and TNC (red). Right panels: αSMA (blue channel, represented in white). TOPRO-3 (far red channel, represented in blue) marks the nuclei. Images representative of whole tumor section areas from *n* = 2 biological replicates. Scale bar: 100 µm. **B**–**E** RNA-seq was performed with *n* = 4 biological replicates for PNPCa and LAPC9 (*n* = 2 technical replicates each), *n* = 3 biological replicates for BM18 (*n* = 2 technical replicates each).

To reveal tumor-induced stromal changes during PCa progression, we performed bulk RNA barcoding and sequencing (BRB-seq)^38^ on the stromal component of PDX models representative of distinct stages of advanced PCa. The PNPCa^27^ (androgen-sensitive and derived from a soft tissue metastasis), BM18^29^ (androgen-sensitive and derived from bone metastasis), and LAPC9^30^ (castration-resistant and derived from bone metastasis) subcutaneous PDX models were used. Sequencing was performed on stromal murine cells obtained directly after tumor tissue digestion and purification from human tumor cells by magnetic-associated cell sorting (MACS) (Fig. 1A). Principal component analysis (PCA) revealed a clear separation of the CRPC-associated LAPC9 stroma from the stromal profile of the androgen-sensitive PNPCa and BM18 models, which clustered together (Fig. 1B).

Analysis of the top 30 stromal differentially expressed genes (DEGs) in the LAPC9 model compared to the PNPCa and BM18 models (Fig. 1C) revealed the presence of ECM-related and ECM-remodeling genes among the cluster of upregulated genes. Several genes involved in ECM deposition, such as collagen type 5 alpha 1 and collagen type 12 alpha 1 (*Col5a1*, *Col12a1)*, collagen and elastin fiber crosslinking enzyme lysyl oxidase like 1 (*Loxl1*), procollagen activation protease bone morphogenetic protein 1 (*Bmp1*), cell-ECM adhesion molecule CD44 (*Cd44*) and the pro-inflammatory gene C-X-C motif chemokine ligand 13 (*Cxcl13*) were among the top upregulated genes in the LAPC9 stroma (Fig. 1C). Pathway analyses indicated that the LAPC9 stroma showed a significant enrichment of gene sets related to “cell chemotaxis”, “leukocyte migration and chemotaxis”, “ECM organization” and “ECM structure organization”, typically enriched in myCAFs (Fig. 1D), suggesting that the LAPC9 stroma shows a pro-fibrotic and pro-inflammatory myCAF-associated phenotype. Indeed, a number of general CAF-associated genes, including vimentin (*Vim*), fibroblast-associated protein (*Fap*) and fibronectin (*Fn1)* as well as a subset of myCAF-associated markers such as collagen 5 a1 (*Col5a1)*, tenascin C *(Tnc)*^8,10^ and neuregulin-1 (*Nrg1)*^24^ was significantly upregulated in the LAPC9 stroma compared to both PNPCa and BM18 stroma, whereby a similar trend was also observed for the myCAF marker alpha-smooth muscle actin (*Acta2)*^8,10^*, and* asporin (*Aspn*)^39^ (Fig. 1E). In contrast, *Col1a1*, *Col1a2* and the myCAF-associated genes NADPH-oxidase 4 (*Nox4*)^40^, secreted phosphoprotein 1 (*Spp1)*^26^ and periostin (*Postn*)^28,39^ were similarly expressed between LAPC9 and PNPCa models but at significantly higher levels than in the BM18 stroma. This suggests that LAPC9 has an advanced myCAF phenotype with PNPCa following a similar, yet less pronounced, trend towards activated myCAF trajectory (*Nox4, Postn*). Melanoma cell adhesion molecule (*Mcam*)^39^, however, showed a trend towards the highest expression in the BM18 model, whereby its expression was significantly higher in the LAPC9 versus PNPCa stroma (Fig. 1E), indicative of pericytes or vascular smooth muscle cells, which is consistent with the high vascularization of this tumor model. Higher expression of CAF genes in the LAPC9 stroma was also validated at the protein level, whereby immunofluorescent staining confirmed higher TNC and α-SMA (encoded by *Acta2*) expression specifically in the stroma of LAPC9 tumors (Fig. 1F).

Collectively, transcriptomic and immunostaining analyses reveal significant differences in the TME of the PNPCa, BM18, and LAPC9 models, whereby the castration-resistant LAPC9 model exhibits a pro-fibrotic myCAF-like phenotype characterized by the upregulation of factors involved in ECM deposition and remodeling.

### LAPC9 tumor cells induce the formation of a fibrotic intratumoral stroma that correlates with a higher prevalence of tumor cell invasion

**Fig. 2:**
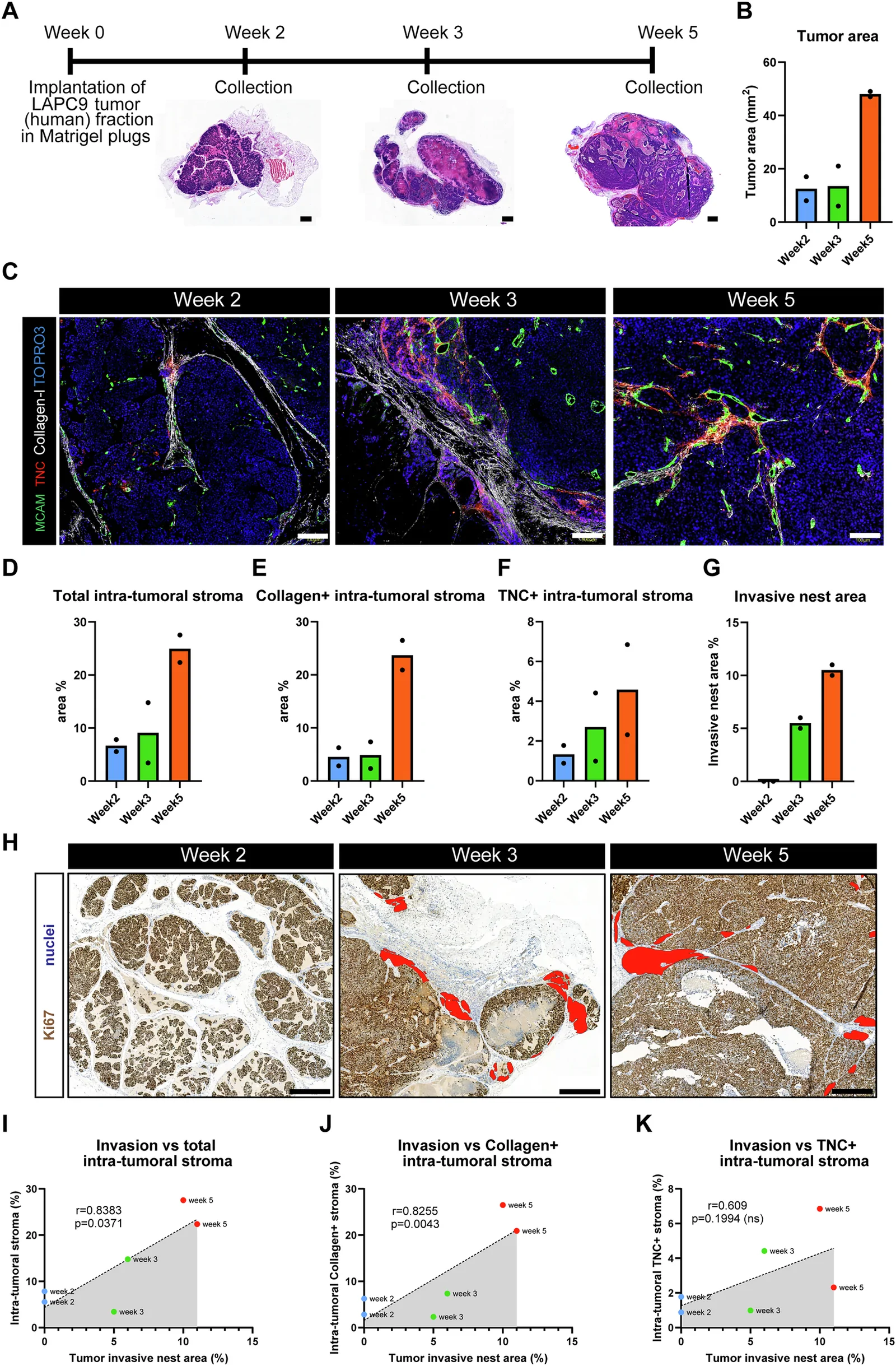
Intratumoral infiltration and deposition of highly fibrotic stroma positively correlate with increased tumor invasiveness during tumor growth. **A** Schematic overview of the LAPC9 stroma infiltration experiment. Briefly, immunocompromised *CB17-SCID* mice were subcutaneously implanted with purified LAPC9 tumor cells in Matrigel plugs. Tumor tissues were collected at weeks 2, 3, and 5 (*n* = 2 tumors/mice per time point) for histological analysis. Representative Hematoxylin & Eosin (H&E) staining images are depicted, scale bar: 500 µm. **B** Bar plots representing the total tumor area quantification (mm^2^) determined through Ki67-staining histopathological analysis. Mean and single measurement from whole tumor areas (dots) reported, *p* = 2 biological replicates per condition. **C** Immunofluorescent staining of MCAM (green), TNC (red), and collagen type-I (blue channel, represented in white). TOPRO-3 (far red channel, represented in blue) marks the nuclei. Scale bar: 100 µm. **D–F** Bar plots reporting the percentage of total intratumoral stroma (**D**), Collagen-positive intratumoral stroma (**E**), and TNC-positive intratumoral stroma (**F**) per time point, based on pathological assessment. The percentage of reported stromal areas was normalized to the total stromal area in each sample. Mean and single measurement from whole tumor areas (dots) reported, *n* = 2 biological replicates per condition. **G** Bar plots representing the percentage of tumor invasive nest area per time point, based on Ki67 histopathological analysis. Mean and single measurement from whole tumor areas (dots) reported, *n* = 2 biological replicates per condition. **H** Representative images of Ki67 immunohistochemistry staining with annotated areas of tumor invasive nests (red) from tumors harvested at the indicated timepoint, Scale bar: 500 µm. **I–K** Correlation plots between the percentage of tumor invasive nest area (normalized to the total tumor area) and the percentage of total intra-tumoral stroma (**I**), collagen-positive (**J**), and TNC-positive (**K**) intratumoral stroma. Pearson correlation coefficient (*r*) and *p*-value (*p*) are reported in each plot; ns = not statistically significant (*p* > 0.05). To visualize the trend between the two variables, a linear regression line (dashed line) is reported in each plot. The time points (week 2, week 3, and week 5) are highlighted with different colors (legends on the upper right of the plots). *n* = 2 biological replicates per condition from whole tumor areas.

To determine how LAPC9 tumor cells induce the formation of a pro-fibrotic stromal phenotype, a time-course assessment of PDX tumor formation was performed. LAPC9 tumor-derived cells were purified from their original stromal component (MACS-mouse cell depletion) and subcutaneously implanted into immunocompromised mice. By harvesting the resulting tumors at different time points post implantation, this approach allowed us to follow and characterize the recruitment and composition of naïve host stromal cells (Fig. 2A) during progressive tumor growth (Fig. 2B). CAFs in the stroma were identified by collagen type-I, TNC, and α-SMA protein expression, and their mouse origin confirmed by positive staining for the mouse-specific antibody cyclophilin A (Fig. 2C and Supplementary Fig. 1A). At week 2, tumor and stromal areas were highly compartmentalized with few cyclophilin A-positive stromal cells found peritumorally, which gradually increased along with α-SMA expression (Supplementary Fig. 1A*)*. However, at later time points, a higher amount of vascularization (MCAM-positive pericytes, Fig. 2C) and intratumoral stroma (Fig. 2D, histopathological evaluation) was detected, with high deposition of TNC (Fig. 2C) and collagen type I (Fig. 2C). Both collagen-positive and TNC-positive intratumoral stromal areas progressively increased across time (Fig. 2E, F, histopathological evaluation). The stroma deposition was independent of the proliferation status of the tumor cells, which were highly proliferative in the majority (Ki67-positive), and at all time points (Supplementary Fig. 1B).

Next, we investigated whether the presence of intratumoral stroma correlates with tumor microinvasion. The tumor-invasive potential was assessed by the presence of small groups of Ki67+ epithelial cells displaying an elongated, tongue-like structure and detached from the main tumor area (hereon termed invasive tumor nests), possibly indicative of tumor cell microinvasion (Supplementary Fig. 1C). Invasive tumor nests were detected at week 3, and in week 5, but not at week 2 (Fig. 2G, H and Supplementary Table 1). This suggests that whilst the LAPC9 tumor cells were highly proliferative from the onset, their invasive capacity is acquired after tumor formation and during tumor growth. In contrast, no or few invasive nests could be detected in PNPCa and BM18 tissues (Supplementary Table 1), in line with the fact that these PDXs represent less aggressive tumor models and they show a slower in vivo tumor growth kinetic (2–3 months for PNPCa, BM18 versus 3–4 weeks for the LAPC9)^27,28^.

Correlation analysis indicated a positive correlation (*r* = 0.8383, *p* = 0.0371) between the quantified area of invasive tumor nests relative to the total area of intratumoral stroma (Fig. 2I). Similarly, the presence of invasive tumor nests was positively correlated with intratumoral collagen-positive (*r* = 0.8255, *p* = 0.0043) (Fig. 2J) as well as TNC-positive stroma (*r* = 0.609, *p*-value not statistically significant) (Fig. 2K), indicative of a linear association of both markers of desmoplastic reaction to tumor invasion features.

Collectively, results thus far suggest that LAPC9 tumor cells modify their surrounding microenvironment, inducing the formation of a desmoplastic, intratumoral stroma characterized by the deposition of collagen and TNC, which may, in turn, facilitate LAPC9 tumor cell invasiveness.

### CRPC-derived myCAFs support the viability of androgen-dependent tumor cells in 3D co-cultures

To specifically investigate the pro-tumorigenic properties of the CRPC-derived stroma, an in vitro model capable of retaining the in vivo PDX-specific stromal and tumor phenotypes was required. We therefore aimed to establish a system in which direct tumor cell–CAF interactions could be studied through the recombination of PDX-derived tumor cells and PDX-derived CAFs in a direct 3D co-culture setting.

PDX-derived stromal cells (fibroblasts) cultured under adherent conditions exhibited an altered transcriptomic profile, even at early in vitro passages, compared to the in vivo setting (Supplementary Fig. 2A–C), indicating the need for ECM support. PDX tumor tissue-derived single cells can be cultured as tumor organoids under ECM-free ultra-low attachment (ULA) conditions. Whilst these culture settings support the growth of prostate luminal epithelial cells^27,33^, they are unsuitable for long-term culture of CAFs. Thus, in the experimental set-up employed herein, PDX-derived tumor cells and CAFs were cultured in a collagen type I matrix. PDX-derived single cells were either first cultured under ULA conditions to enhance organoid formation, before subsequent transfer of the organoids into collagen, or directly seeded into collagen to enable organoid formation directly in the ECM. In both approaches, CAF adhesion to plastic was prevented in order to avoid possible phenotypic alterations, but most importantly, the tumor-CAF cell-cell contact was constantly maintained.

**Fig. 3:**
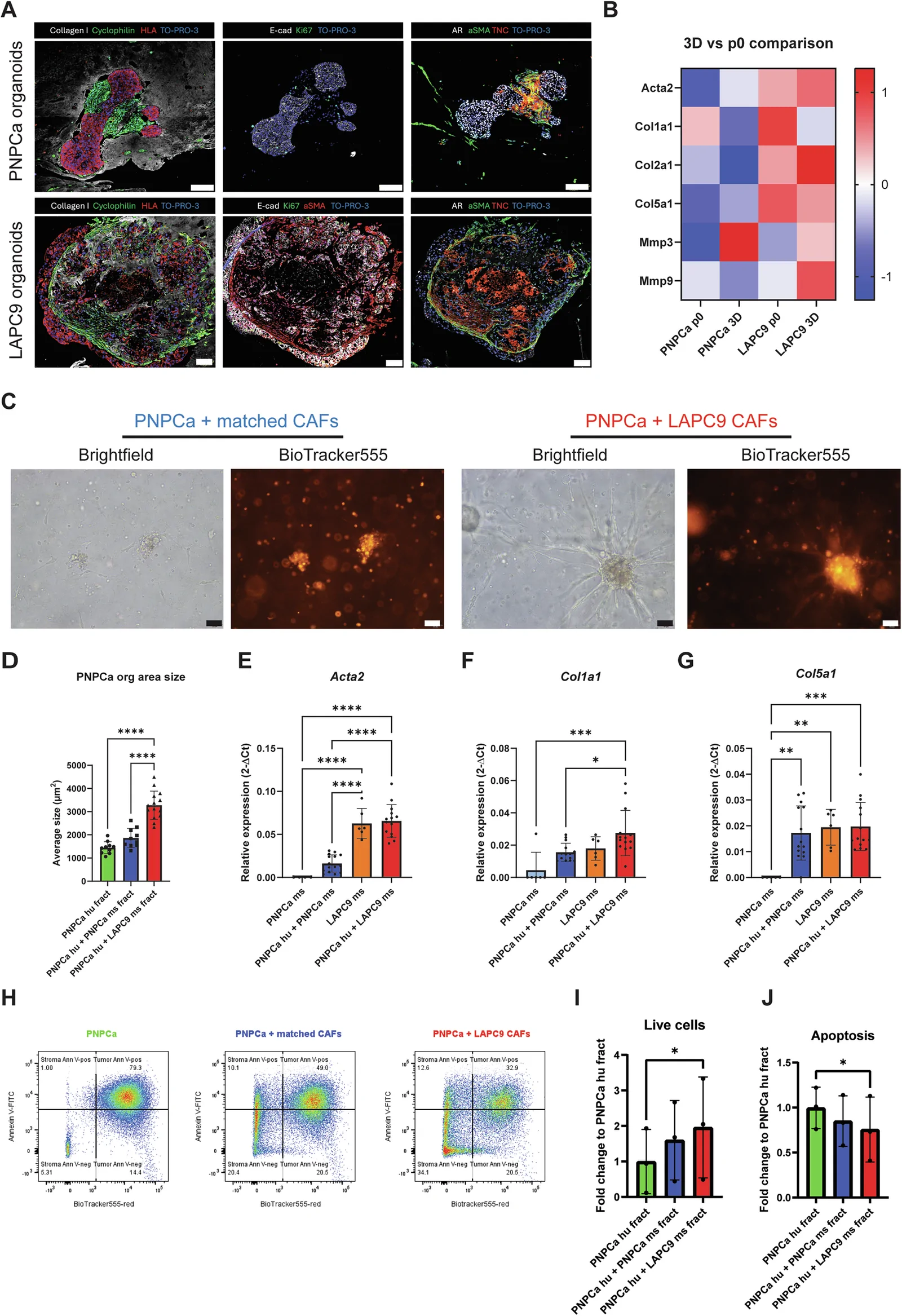
LAPC9-derived CAFs support PNPCa tumor cell viability in hybrid tumor-CAF 3D collagen co-cultures. **A, B** In the experiments described, PNPCa and LAPC9 organoids were pre-formed in ULA and subsequently transferred into a collagen matrix upon organoid formation (after 5–7 days). **A**. Immunofluorescent staining of FFPE sections of PNPCa and LAPC9 tumor-CAF co-cultures after 7 days of collagen co-culture. Left panels: collagen type-I (blue channel, represented in white), cyclophilin A (green), HLA (red). Middle panels: E-cadherin (blue channel, represented in white), Ki67 (green), α-SMA (red). Right panels: AR (blue channel, represented in white), α-SMA (green), TNC (red). TOPRO-3 (far red channel, represented in blue) marks the nuclei. Scale bar: 100 µm. **B** Heatmap representing the RNA expression of *Acta2*, *Col1a1*, *Col2a1*, *Col5a1*, *Mmp3*, and *Mmp9* in PNPCa and LAPC9 stromal cells after PDX tissue digestion (p0), and in collagen tumor-CAF 3D co-cultures (3D). The color code reports the mean of the logarithm of the relative expression (log(2^−∆Ct^)) of the indicated gene to *Actb* (housekeeping gene), with blue indicating lower expression, and red higher expression. Row-wise *Z*-score normalization per gene across samples was performed for the heatmap visualization, *n* = 2 biological replicates with each *n* = 3 technical replicates per condition. **C–J** In the experiment described, hybrid co-cultures were first generated in ULA and subsequently transferred into a collagen matrix upon organoid formation (Day 5–7). **C** Representative brightfield and red fluorescence images of PNPCa tumor + LAPC9 CAF and PNPCa + matched CAF collagen co-cultures. PNPCa tumor cells have been labeled with BioTracker™ 555 Orange Cytoplasmic Membrane Dye prior to recombination with PNPCa or LAPC9 stromal cells. Scale bar: 50 µm. **D** Measurement of PNPCa organoid average area size (µm^2^) in PNPCa tumor (human; hu) cell monocultures, PNPCa tumor + PNPCa CAF (mouse; ms), and PNPCa tumor + LAPC9 CAF co-cultures performed with Fiji software on multiple brightfield images of *n* = 2 biological replicates. Mean, standard deviation, and every single measurement (dots, technical and biological replicates) are reported. Ordinary one-way ANOVA with Tukey’s multiple comparison, *p* < 0.05 (*), *p* < 0.01 (**), *p* < 0.001 (***), *p* < 0.0001 (****). **E–G** RT-qPCR was performed on RNA extracted from PNPCa tumor (hu) + PNPCa CAF (ms), and PNPCa tumor + LAPC9 CAF collagen co-cultures, PNPCa CAF, and LAPC9 CAF collagen mono-cultures using mouse-specific primers. Mean, standard deviation, and every single value (technical and biological replicates) of the logarithm of the relative expression (log(2^−∆Ct^)) of the indicated genes relative to *Actb* (housekeeping gene) are reported, *n* = 5 biological replicates with each *n* = 3 technical replicates for co-culture conditions, *n* = 2 biological replicates with each *n* = 3 technical replicates for mono-culture conditions. Ordinary one-way ANOVA with Tukey’s multiple comparison, *p* < 0.05 (*), *p* < 0.01 (**), *p* < 0.001 (***), *p* < 0.0001 (****). **H** Representative flow cytometry analysis (dot-plots) of PNPCa + LAPC9 CAFs, PNPCa + matched CAFs, or PNPCa monocultures, performed at co-culture Day 4. Annexin V-FITC staining was used to discriminate live (Annexin V -) and apoptotic (Annexin V +) tumor cells (BioTracker555-positive) and CAFs (BioTracker555-negative). **I-J** Fold change of the fraction of viable (**I**) and apoptotic (**J**) cells compared to the monoculture control for *n* = 3 independent flow cytometry experiments (biological replicates). Mean, standard deviation, and every single measurement (dots) are reported. Statistics: Friedman test with Dunn’s multiple comparison.

Given that PNPCa organoid formation could not be achieved upon direct seeding of single cells into collagen, PNPCa organoids were pre-formed in ULA and subsequently transferred into the collagen matrix (Supplementary Fig. 3A). PDX organoid staining under ULA conditions revealed the presence of cells positive for cyclophilin-A (mouse origin), indicating that host stromal cells were retained in the organoid structure under suspension conditions (Supplementary Fig. 3B), even though they did not display their typical morphological appearance due to the lack of ECM and cell adherence. Upon transfer of PNPCa and LAPC9 organoids into a collagen type-I ECM support, stromal cells started budding out from the organoid structure and displayed a typical elongated fibroblast morphology (Supplementary Fig. 3C). These stromal-derived CAFs co-expressed cyclophilin-A and were positive for the CAF markers α-SMA and TNC, whereas tumor cells within the organoid structure expressed human leukocyte antigen (HLA), indicative of their human origin, the epithelial marker E-cadherin and AR (Fig. 3A). Moreover, proliferating Ki67+ tumor epithelial cells were detected in both PNPCa and LAPC9 organoids (Fig. 3A). Real-time quantitative PCR (RT-qPCR) analysis by using stromal (mouse)-specific primers of RNA extracted from the generated tumor-CAF collagen 3D co-cultures revealed higher expression of typical myCAF marker genes (*Acta2, Col1a1, Col2a1, Col5a1*, and *Mmp9*) in LAPC9 versus PNPCa co-cultures, similar to LAPC9 versus PNPCa stroma after PDX tissue digestion (p0) (Fig. 3B). On the contrary, CAF-associated gene expression was lower in PNPCa and LAPC9 fibroblasts cultured in 2D except for *Acta2* (Supplementary Fig. 2C), which was higher than p0 and 3D conditions, in line with the fact that fibroblast adhesion to plastic induces α-SMA upregulation^21^. This analysis also revealed lower *Col1a1* expression in the collagen 3D co-cultures versus the PDX stroma (Fig. 3B), probably due to the fact that these cells are grown in a collagen type I matrix.

We next tested the capability of the newly developed collagen-based direct co-culture system to preserve the pro-fibrotic and pro-tumorigenic features of the LAPC9 CRPC stroma in vitro. Specifically, we recombined LAPC9-derived CAFs with androgen-dependent PNPCa tumor cells (“hybrid” co-cultures) to determine their effect on PNPCa tumor cell viability and growth compared to PNPCa-derived CAFs (Supplementary Fig. 4A). The selection of these tumor models for the hybrid co-cultures was to isolate effects attributable to androgen sensitivity while minimizing other stromal differences; transcriptomic proximity (PCA and CAF‑marker trends) indicates PNPCa stroma is more similar to LAPC9 than BM18, despite disease stage, making PNPCa the more appropriate androgen‑sensitive comparator. Morphological analysis revealed the presence of more elongated CAFs in the hybrid versus the matched tumor-CAF co-cultures (Fig. 3C). Moreover, PNPCa organoids co-cultured with CRPC LAPC9 CAFs (hybrid co-culture) exhibited a significantly larger organoid size compared to PNPCa organoids cultured with their matched CAFs (average organoid area size of 3275.8 μm^2^ for hybrid CAF co-cultures vs. 1864.7 μm^2^ and 1443.8 μm^2^ for matched CAF co-cultures and monoculture controls, respectively, Fig. 3D). RT-qPCR using mouse (stromal)-specific primers revealed higher expression of the CAF-related genes *Acta2*, *Col1a1* (statistically significant), *Col5a1*, and *Tnc* (trend toward higher expression, but not statistically significant) in the hybrid versus the matched co-cultures supporting the “reactive” fibrotic phenotype of the LAPC9 CAFs in in vitro 3D settings (Fig. 3E–G, and Supplementary Fig. 4B). Moreover, PNPCa CAFs showed higher *Acta2*, *Col1a1*, and *Col5a1* expression upon co-culture with PNPCa tumor cells with respect to PNPCa CAF collagen monocultures (in which the expression of these genes was barely detectable), while LAPC9 CAFs showed a similar gene expression irrespective of co-culture with PNPCa cancer cells (Fig. 3E–G). These data overall suggest that the pro-tumorigenic properties of the LAPC9 CRPC stroma are intrinsic and are retained in collagen 3D (co)-cultures.

To corroborate the pro-tumorigenic effect of LAPC9 CAFs on PNPCa organoid viability, we performed flow cytometry analysis for the apoptotic marker Annexin V on dissociated tumor organoids and CAFs after collagen co-culture. Prior to co-culture recombination, PNPCa tumor cells obtained after PDX tissue digestion were labeled with BioTracker^TM^555, a lipophilic dye that binds to the phospholipid cell membrane (Fig. 3C) enabling discrimination of tumor cells (labeled) and stromal cells (unlabeled) in the flow cytometry analysis (Fig. 3H, and Supplementary Fig. 4C). In line with the organoid area quantification data, analysis of Annexin V-negative (live cells) and Annexin V-positive (apoptotic cells) tumor (BioTracker555+) cells indicated a higher percentage of living cells and a lower percentage of apoptotic cells for PNPCa organoids in the hybrid versus the matched CAF condition (Fig. 3H–J and Supplementary Fig. 4D). Similarly, PNPCa organoids in the matched co-cultures displayed a higher percentage of living cells and a lower percentage of apoptotic cells than PNPCa monocultures (Figs. 3H–J and Supplementary Fig. 4D).

In summary, the direct 3D tumor-CAF collagen co-cultures established herein can mimic, at least partially, features of the parental tumor tissue as demonstrated by the presence of LAPC9-derived CAFs with a myCAF phenotype similar to the in vivo setting. Furthermore, higher PNPCa organoid proliferation and viable tumor cell index in hybrid tumor-CAF co-cultures strongly suggest that CRPC-derived CAFs possess pro-survival and pro-tumorigenic properties.

### Spi1 is a master regulator of the fibroblast-mediated reactive stroma response in LAPC9 stromal cells

**Fig. 4:**
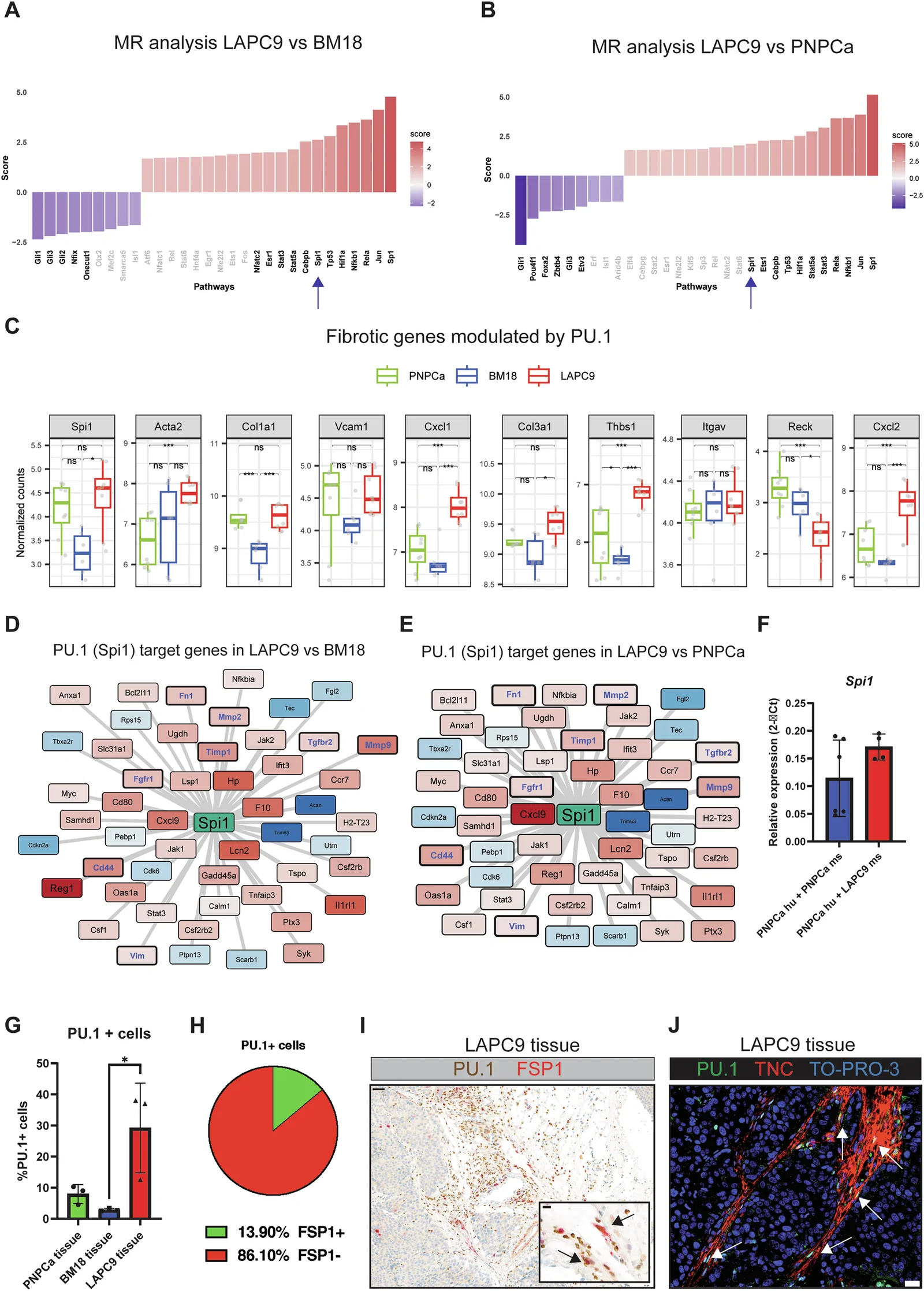
*Spi1*, coding for the transcription factor PU.1, is a potential mediator of the LAPC9 pro-fibrotic phenotype. **A-B** Bar plots representing the MR (transcription factors) showing higher/lower activity (score) in LAPC9 vs BM18 stroma (**A**) and LAPC9 vs PNPCa stroma (**B**). *Spi1* is marked with an arrow. Genes highlighted in bold show a statistically significantly higher/lower activity (*p*-value < 0.05). **C** Boxplots for the expression levels (normalized counts DEseq2) of fibrotic genes modulated by PU.1 transcriptional activity in p0 PNPCa, BM18, and LAPC9 stromal cells based on the transcriptomic data. *n* = 4 biological replicates for PNPCa and LAPC9 (*n* = 2 technical replicates each), *n* = 3 biological replicates for BM18 (*n* = 2 technical replicates each). Statistics: Wald test with Benjamini and Hochberg multiple correction, *p* < 0.05 (*), *p* < 0.01 (**), *p* < 0.001 (***). **D, E** Network representation of *Spi1* target genes according to the MR analysis performed. The box color scale indicates higher (red)/lower (blue) expression of the reported gene in the LAPC9 vs BM18 (**D**) and LAPC9 vs PNPCa (**E**) stroma comparison. Gene symbols in blue font (outlined boxes) denote known ECM/CAF-related genes. **F** RT-qPCR performed on RNA extracted from PNPCa (human; hu) + LAPC9 CAFs (mouse; ms) and PNPCa + matched CAFs organoid hybrid co-cultures using mouse-specific primers. Mean, standard deviation, and each single value (technical and biological replicates) of the logarithm of the relative expression (log(2^−∆Ct^)) of *Spi1* gene to *Actb* and *Hprt* (housekeeping genes) is reported, *n* = 2 biological replicates with each *n* = 3 technical replicates per condition. Unpaired t-test, *p* < 0.05 (*), *p* < 0.01 (**), *p* < 0.001 (***), *p* < 0.0001 (****). **G** PU.1-CD45 double IHC staining quantification analysis. The bar plot represents the mean, standard deviation, and every single measurement (dots) of the percentage of PU.1-positive cells over the total number of non-epithelial cells detected in PNPCa, BM18, and LAPC9 tissues. Quantifications performed over *n* = 3 biological replicates per sample. Statistics: Kruskal–Wallis test with Dunn’s multiple comparison, *p* < 0.05 (*), *p* < 0.01 (**), *p* < 0.001 (***), *p* < 0.0001 (****). **H** PU.1-FSP1 double-IHC staining quantification analysis. Pie chart reporting the percentage of FSP1+ (green) and FSP1- (red) cells over the total of PU.1+ cells in LAPC9 tissue. The percentage is the average of *n* = 3 biological replicates. **I** Representative double-IHC staining for PU.1 (brown) and FSP-1 (red) performed on LAPC9 tissue. In the magnification, examples of PU.1 and FSP-1 double-positive cells are reported (arrows). Scale bar: 50 µm (full image and magnification). **J**. Immunofluorescent staining of LAPC9 tissue depicting PU.1 (green) and TNC (red). TOPRO-3 (far red channel, represented in blue) marks the nuclei. Arrows indicate examples of PU.1-positive cells in TNC-positive stromal areas. Representative image of *n* = 2 biological replicates. Scale bar: 20 µm.

To identify genes and molecular pathways involved in regulating the fibrotic response in the LAPC9 stroma, we analyzed the activity of transcription factors, also referred to as master regulators (MRs), in the gene expression profiles of the PNPCa, BM18, and LAPC9 stroma by using the decoupleR computational methods^41^. MRs that showed a significantly higher enrichment score in the LAPC9 stroma versus the androgen-sensitive PDX models included nuclear factor kappa B subunit 1 (*Nfkb1*), Jun Proto-Oncogene (*Jun*), Sp1 transcription factor (*Sp1*), ETS proto-oncogene 1 transcription Factor (*Ets1*), signal transducer and activator of transcription 3 (*Stat3*), and Spi1 proto-oncogene (*Spi1*), while the Hedgehog signaling (Hh) pathway GLI family zinc finger 1 (*Gli1*) and 3 (*Gli3*) MRs showed higher activity in both PNPCa and BM18 (Fig. 4A, B) with respect to LAPC9 stroma. Enrichment of *Sp1*, *Nfkb1*, and *Spi1* activity in the LAPC9 versus BM18 stroma was further validated via MR analysis of the DEG from our previously reported independent bulk RNA-seq dataset on different biological replicates^28^ (Supplementary Fig. 5A). Studies have shown that *Nfkb1*, *Jun*, *Sp1*, *Ets1*, *Stat3*, and *Spi1* mediate the TGFβ-dependent fibrotic response in fibroblasts and tissue fibrosis, with *Sp1*, *Ets1*, and *Jun* inducing *TNC* expression, in line with the observed phenotype^42–47^. Enrichment of *Jun* and *Spi1* activity was also found in the PNPCa versus the BM18 stroma (Supplementary Fig. 5B). Enrichment of *Gli1* and *Gli3*, the transcription factors that transduce Hh signaling pathway activity, in the stroma of the androgen-sensitive and low-tumorigenic PNPCa and BM18 PDXs (Fig. 4A, B and Supplementary Fig. 5A) suggests higher stromal Hh signaling activity in these models. This was confirmed by lower levels of the Hh pathway-related genes *Boc, Gli1, Gli2, Hhip, Ptch1*, and *Smo* in the LAPC9 versus the PNPCa and BM18 stroma assessed through transcriptomic dataset analysis (Supplementary Fig. 5C). Lower *Gli1* and *Gli2* expression in LAPC9 versus PNPCa and BM18 was also confirmed by RT-qPCR analysis of the PDX stromal fraction (Supplementary Fig. 5D). This is consistent with a study showing that stromal Hh signaling restrains PCa progression by maintaining the smooth muscle cell layer and preventing tumor cell micro-invasion^48^.

Among the MR genes showing higher activity in the LAPC9 stromal cells, *Spi1*, which encodes the transcription factor PU.1, was further investigated due to its documented role in promoting wound healing and fibrosis in multiple organs and due to the fact that a specific small molecule inhibitor is available (DB1976)^47,49^. To ascertain whether PU.1 may modulate the LAPC9 pro-fibrotic phenotype, we assessed in our RNA-seq dataset the expression of a pro-fibrotic gene signature known to be modulated by pharmacological inhibition of PU.1 activity^47^. This confirmed enrichment for the *Spi1-* target genes; C-X-C motif chemokine ligand 1 (*Cxcl1)*, *Acta2*, *Col3a1*, thrombospondin-1 *(Thbs1)*, C-X-C motif chemokine ligand 2 (*Cxcl2)*, in the LAPC9 versus PNPCa and BM18 stroma (even though *Acta2* showed enrichment only in LAPC9 versus PNPCa stroma, and *Col3a1* only in LAPC9 versus BM18 stroma) (Fig. 4C). Higher expression of *Acta2*, *Thbs1*, and *Col1a1* was also determined via RT-qPCR analysis of the PDX stromal fraction, and *Spi1* showed higher expression in the LAPC9 versus the other two models (even though the difference was statistically significant only in LAPC9 versus BM18) (Supplementary Fig. 5E). In further support of these findings, we observed that among the *Spi1* target genes (identified by the MR analysis) upregulated in the LAPC9 versus BM18 and LAPC9 versus PNPCa stroma (*p*-value < 0.01), several encode known ECM-remodeling and CAF-related genes^8^, including matrix metallopeptidase 2 (*Mmp2)*, matrix metallopeptidase 9 (*Mmp9), Cd44*, TIMP metallopeptidase Inhibitor 1 *(Timp1*), fibroblast growth factor receptor 1 *(Fgfr1)*, fibronectin 1 *(Fn1)*, transforming growth factor beta receptor 2 *(Tgfbr2)*, and *Vim* (Fig. 4D, E and Supplementary Table 2). Furthermore, RT-qPCR using mouse-specific primers of PNPCa hybrid (*PNPCa tumor* + *LAPC9 CAFs*) versus PNPCa-matched (*PNPCa tumor* + *CAFs*) collagen co-cultures (described in in Fig. 3) revealed a trend toward higher *Spi1* expression in LAPC9-derived CAFs versus PNPCa-derived CAFs (Fig. 4F). This difference was not statistically significant; however, this was similar to what observed for the *Spi1* gene expression in LAPC9 versus PNPCa PDX-derived (p0) stromal cells (Fig. 4C and Supplementary Fig. 5E). Collectively, these data suggest that PU.1 is a mediator of the LAPC9 stroma CRPC-associated pro-fibrotic phenotype.

At the protein level, the total number of PU.1-positive stromal cells was higher in the LAPC9 tissue (around 30%), compared to PNPCa and BM18 tissues (<10%) (Fig. 4G and Supplementary Fig. 6A), validating the transcriptomic data. PU.1 is a transcription factor known to be involved not only in fibrosis (as described so far), but also in hematopoietic cell maturation, playing a role in monocyte and B cell differentiation^50^. *Spi1* gene expression alterations result in hematopoietic abnormalities such as leukemia^51^. Therefore, regarding their cellular identity, PU.1 expressing cells may be of fibroblast or myeloid lineage (monocyte, macrophage), but not of lymphoid lineage origin, given that the PDX tumors are maintained in immunocompromised *CB17-SCID* mice, which lack T and B cells. Immunohistochemical analysis revealed that in LAPC9 tumor tissues, PU.1 is almost exclusively co-expressed with CD45 (PTPRC) marker of hematopoietic/immune-derived cells (Supplementary Fig. 6A, B). Western Blot analysis on EpCAM+ tumor cells, CD45- and CD45 + FACS-sorted cell lysates (Supplementary Fig. 6C) confirmed that PU.1 expression is restricted to the CD45+ stromal fraction (Supplementary Fig. 6D). CD45 expression can be either associated with fibrocytes, i.e., cells of hematopoietic origin that are recruited from the bone marrow into different tissues, and display expression of fibroblast/MFB markers (e.g. collagen type I, αSMA) contributing to tissue fibrosis through ECM secretion and remodeling^52–55^, or with myeloid-derived macrophages that transdifferentiate into MFBs (monocyte/macrophage-to-myofibroblast transition, or MMT)^56–58^. In fact, studies show that fibroblast-like cells in vivo can be CD45-positive, while they typically lose CD45-positivity during in vitro culturing^59^. To determine whether CD45-positive cells possess fibroblast properties, we performed immunostaining for the fibroblast marker Fibroblast-specific protein-1 (FSP-1)^52^. Double-IHC quantification analysis for PU.1 and FSP-1 revealed that the LAPC9 stroma contains approximately 15% of double PU.1- and FSP1-positive cells (Fig. 4H, I), suggesting that a subset of PU.1 -positive (and CD45-positive) cells display clear fibroblast properties (possibly fibrocytes), contributing to the LAPC9 stroma pro-fibrotic phenotype. This was also supported by the elongated nucleus morphology of PU.1 and FSP-1 double-positive cells, a typical feature of fibroblasts (Fig. 4I, magnification). Furthermore, IF analysis showed co-localization of PU.1-positive cells with TNC, α-SMA, and collagen-type-I -positive stromal areas (Fig. 4J and Supplementary Fig. 6E, F), supporting the fact that PU.1-positive cells contribute to the pro-fibrotic phenotype. No expression of PU.1 was detected in LAPC9 tumor cells (Fig. 4I, J and Supplementary Fig. 6A, D, E, F).

In summary, we identified the *Spi1* gene, coding for the transcription factor PU.1 and expressed by cells of hematopoietic origin (CD45-positive) displaying MFB/CAF properties, as a specific MR of the CRPC-CAF phenotype.

### Blockade of PU.1 activity inhibits the myofibroblast CAF phenotype induction, which impacts tumor organoid growth

**Fig. 5:**
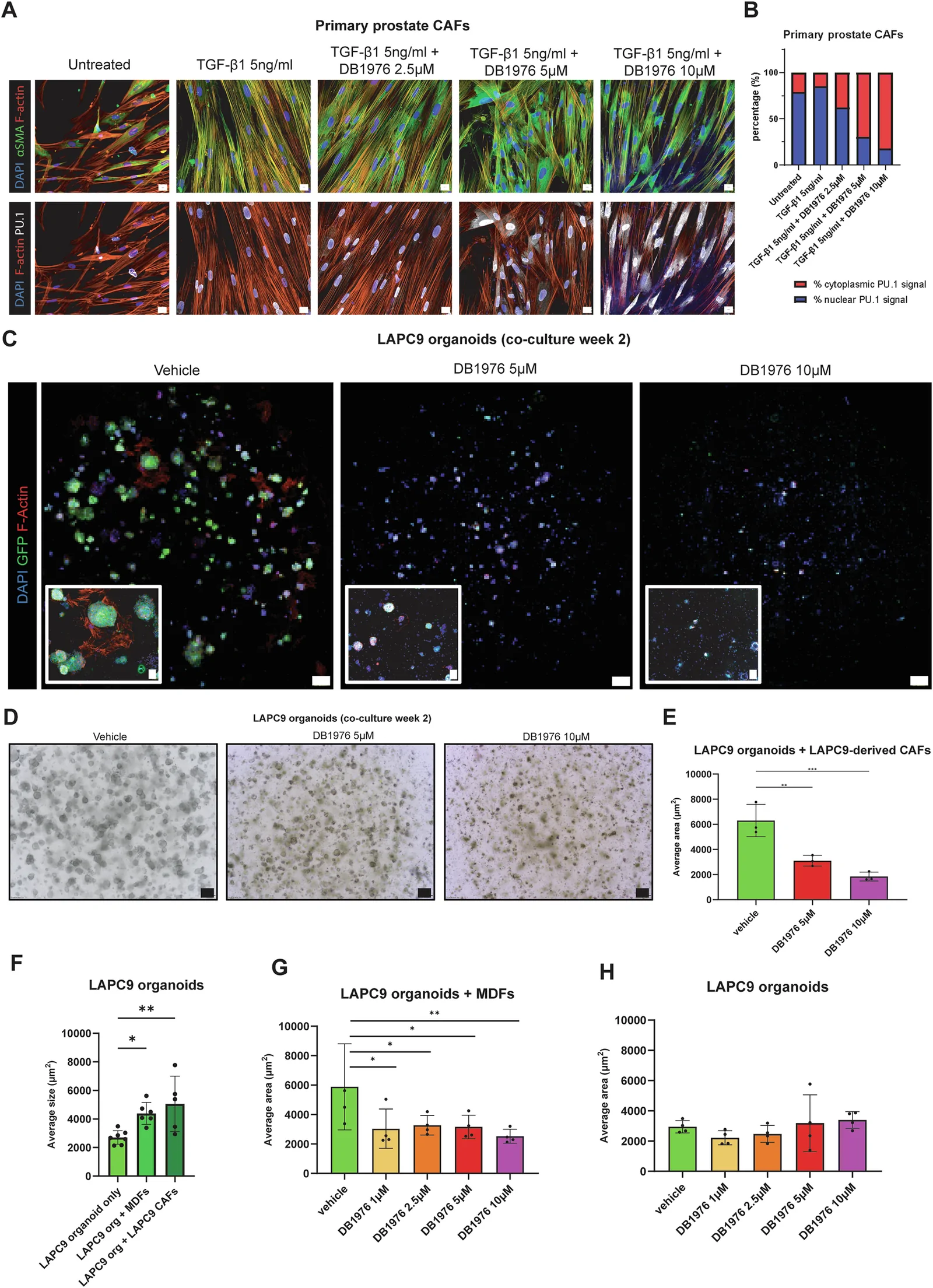
Blockade of PU.1 activity inhibits the myofibroblast phenotype, indirectly counteracting tumor organoid formation. **A** Whole-mount staining of formalin-fixed human primary prostate CAFs after co-treatment with 5 ng/ml TGFβ1 and the indicated concentration of DB1976 or vehicle equivalent for 5 days under serum-starved conditions. Top panels: α-SMA (green), F-actin (red), bottom panels: F-actin (red), PU.1 (far red channel, represented in white). DAPI (blue) marks the nuclei. Scale bar: 20 µm. **B** PU.1 nuclear (blue bars) and cytoplasmic (red bars) signal quantification analysis performed with the Fiji software based on the primary CAF immunofluorescence images described in *A (bottom row)*. PU.1 nuclear signal was defined by co-localization with DAPI signal (marker for cell nuclei). **C** Whole-mount fluorescence staining of formalin-fixed LAPC9-GFP tumor-matched CAF collagen co-cultures (unsorted tumor and stromal cells) stained with Phalloidin (to visualize F-actin) after 2-week DB1976 treatment at the indicated concentration. Representative images of *n* = 3 technical replicates. GFP (green), F-actin (red). DAPI (blue) marks the nuclei. Scale bar: 200 µm (full images), 20 µm (magnifications) and **D** brightfield imaging of the same co-cultures (LAPC9-GFP + LAPC9 CAFs). Representative image of *n* = 3 technical replicates. Scale bar: 200 µm**. E** Measurement of the average organoid area size (µm^2^) based on brightfield images (**D**) of LAPC9 organoids +CAF (unsorted), performed with the Fiji software after DB1976 2-week treatment. Mean, standard deviation, and every single measurement (dots) are reported. Ordinary one-way ANOVA with Dunnett’s multiple comparison, *p* < 0.05 (*), *p* < 0.01 (**), *p* < 0.001 (***), p < 0.0001 (****), *n* = 3 image replicates per condition. **F** Fiji quantification of average organoid area size (µm^2^) based on brightfield images of LAPC9-GFP organoids in collagen monoculture, co-cultured with MDFs, or LAPC9 CAFs (unsorted). Mean, standard deviation, and every single measurement (dots) are reported. Ordinary one-way ANOVA with Tukey’s multiple comparisons, *p* < 0.05 (*), *p* < 0.01 (**), *p* < 0.001 (***), *p* < 0.0001 (****), *n* = 4 image replicates per condition. **G–H** Fiji quantification of average organoid area size (µm^2^) based on brightfield images of LAPC9-GFP organoids co-cultured with MDFs (**G**), or in monoculture (**H**) after 2-week DB1976 treatment. Mean, standard deviation, and every single measurement (dots) are reported. Ordinary one-way ANOVA with Dunnett’s multiple comparisons, *p* < 0.05 (*), *p* < 0.01 (**), *p* < 0.001 (***), *p* < 0.0001 (****), *n* = 4 image replicates per condition.

PU.1 represents an attractive therapeutic target because its expression is associated with the TME (CAF and immune cells) and because of the possibility of selectively inhibiting its transcriptional activity. To validate the functional effect of PU.1 on MFB phenotype induction, human primary PCa-derived CAFs were serum-starved, stimulated with the pro-fibrotic TGF-β1 growth factor to induce PU.1 expression, and subsequently co-treated with increasing doses of the PU.1 inhibitor compound DB1976. Upon TGFβ1 treatment, aligned and elongated filamentous actin (F-actin) stress fibers became apparent, as visualized using phalloidin staining (Fig. 5A). The incorporation of α-SMA in the phalloidin-positive stress fibers and PU.1 nuclear expression by CAFs was detected upon TGF-β1 treatment, confirming the role of PU.1 downstream of TGFβ-signaling in MFB transition (Fig. 5A). Upon DB1976 treatment (2.5 μM, 5 μM, and 10 μM doses), PU.1 was partially re-localized to the cytoplasm (Fig. 5A), as confirmed by cytoplasmic/nuclear quantification analysis of PU.1 signal (Fig. 5B), suggesting inhibition of its transcriptional activity. Concomitantly, we observed a partial reversion of the MFB phenotype with loss of α-SMA localization to the actin fibers (Fig. 5A). We also validated the PU.1 role in acquisition of MFB phenotype upon TGF-β1 treatment in mouse dermal fibroblasts (MDFs), representing non-cancerous fibroblasts, observing similar results to those reported for prostate CAFs (Supplementary Fig. 7A, B). Moreover, the reduction of phalloidin-positive stress fibers upon DB1976 treatment was also observed in MDFs cultured in a 3D setting in a collagen matrix (Supplementary Fig. 7C).

We then tested the effect of CAF-specific PU.1 activity inhibition on tumor organoid formation using our collagen-based direct tumor-CAF 3D co-culture system. To this end, LAPC9-tissue digested tumor (GFP-labeled) and matched stromal single cells (unsorted) were directly seeded into collagen to allow organoid formation in the presence/absence of DB1976, thus ensuring a robust comparison between treated and untreated settings. Compared to the growth of PNPCa and LAPC9 organoids in ULA (suspension) settings, LAPC9 organoids required a longer time to form in collagen (14–17 days versus 6–7 days), reaching a smaller diameter size (average 100 μm diameter in collagen versus average 210 μm diameter in ULA) (Supplementary Fig. 8A, B).

Following a 2-week DB1976 treatment (2.5 μM, 5 μM, and 10 μM doses) a reduction of CAF F-actin stress fibers (stained with phalloidin) was observed compared to vehicle controls (Fig. 5C and Supplementary Fig. 8C). Whilst LAPC9 tumor co-culture organoids formed upon DB1976 treatment, the resulting organoids were smaller in size, indicative of a lower proliferation rate compared to the vehicle condition (Fig. 5C, D and Supplementary Fig. 8C). This was confirmed by imaging-based quantification of the organoid area size (Fig. 5E). We also performed DB1976 treatment (5 μM and 10 μM doses) in LAPC9 tumor-CAF co-cultures in which the organoids were pre-formed in ULA and then transferred to collagen. Similarly, we observed a reduction of phalloidin-positive stress fibers and the presence of organoids of smaller size upon treatment, even though organoids were overall larger due to the ULA pre-formation step (Supplementary Fig. 8D). In this setting, we also observed a reduced tendency of LAPC9 organoids to localize toward the edges of the collagen plugs upon DB1976 treatment (Supplementary Fig. 8D). In addition, we determined that there is no additive effect upon combined PU.1 activity inhibition and DHT removal (Supplementary Fig. 9A), demonstrated by the fact that there was a reduction in organoid number (Supplementary Fig. 9B) and average size (Supplementary Fig. 9C) both upon DB1976 treatment and DHT removal. Still, LAPC9 organoids could form even in the absence of androgens, in line with the fact that the LAPC9 represents a castration-resistant PCa model^27,28^. Moreover, the observed effect upon DB1976 treatment is mostly due to PU.1 activity inhibition in PU.1-positive fibroblast/MFB-like cells rather than myeloid PU.1-positive cells. Indeed, RT-qPCR analysis on LAPC9 tumor-CAF co-cultures after a 2-week DB1976 treatment revealed that the treatment reduces *Acta2* expression levels, while the expression of myeloid and pro-inflammatory marker genes (*Cd45, Cd11b, Csf2, Il1a, Il1b, Il6, Tnf*) did not show significant changes upon treatment and their expression was overall lower than *Acta2* (Supplementary Fig. 9D). However, the fact that *Cd45* expression is lower than *Acta2* (Supplementary Fig. 9D) possibly indicates a low number of CD45-positive cells in LAPC9 tumor CAF co-cultures. This differs from what was observed in the LAPC9 in vivo tissue, in which there is a high abundance of PU.1 and CD45-positive cells (Supplementary Fig. 6A, B), thus indicating that in vitro 3D culturing might induce phenotypical changes in PU.1-positive fibroblast-like cells, such as loss of *Cd45* expression, as already suggested in other studies^59^. To determine whether this process is specific to LAPC9-matched CAFs or not, we generated LAPC9 tumor-fibroblast collagen co-cultures by recombining LAPC9 tumor cells (MACS-depleted of their original stroma) with non-tumorigenic MDFs. Area size quantification of LAPC9 organoids co-cultured with LAPC9-derived CAFs (as previously described) or with non-tumorigenic MDFs revealed that the presence of fibroblasts, regardless of their origin, supports LAPC9 organoid formation with respect to LAPC9 organoid monocultures (Fig. 5F), differently from what was observed for PNPCa co-cultures in which LAPC9-derived CAFs showed a stronger pro-tumorigenic effect than matched PNPCa CAFs (described in Fig. 3). We then treated LAPC9 organoids co-cultured with MDFs or in monoculture with DB1976 from day 0 (at single-cell seeding). Like the aforementioned data, stress fiber formation and organoid size were decreased in the co-cultures treated with DB1976 (Fig. 5G and Supplementary Fig. 10A, B). Similar doses of DB1976, however, did not affect the organoid size of monocultured LAPC9 tumor cells (Fig. 5H and Supplementary Fig. 10C), confirming that DB1976 specifically acts on the co-cultured fibroblasts. Furthermore, organoids in monoculture showed a similar size to the organoids in co-culture treated with DB1976 (Fig. 5G treated conditions, Fig. 5H), further underscoring the strong inhibitory effect of DB1976 on fibroblast-driven organoid growth. Finally, we performed propidium iodide (PI) and Hoechst3342 staining to assess the effect of DB1976 on the viability of the LAPC9 tumor + MDF co-cultures and LAPC9 tumor monocultures. These analyses confirmed that most of the LAPC9 organoids and fibroblasts were viable (PI-negative and Hoechst3342-positive) under both vehicle and treated conditions (Supplementary Fig. 10B, C), indicating that DB1976 does not affect organoid or CAF viability at the doses employed.

Altogether, these data suggest that LAPC9 tumor cells influence the phenotype of adjacent fibroblasts, either non-tumorigenic MDFs or pro-tumorigenic LAPC9 CAFs, inducing a “typical” pro-fibrotic MFB phenotype to support their own growth; pharmacological blockade of PU.1 activity inhibits the pro-fibrotic phenotype, thus impairing tumor organoid formation and growth, without directly affecting tumor cell or fibroblast viability.

### SPI1/PU.1 expression is associated with AR-negative and non-neuroendocrine CRPC

**Fig. 6:**
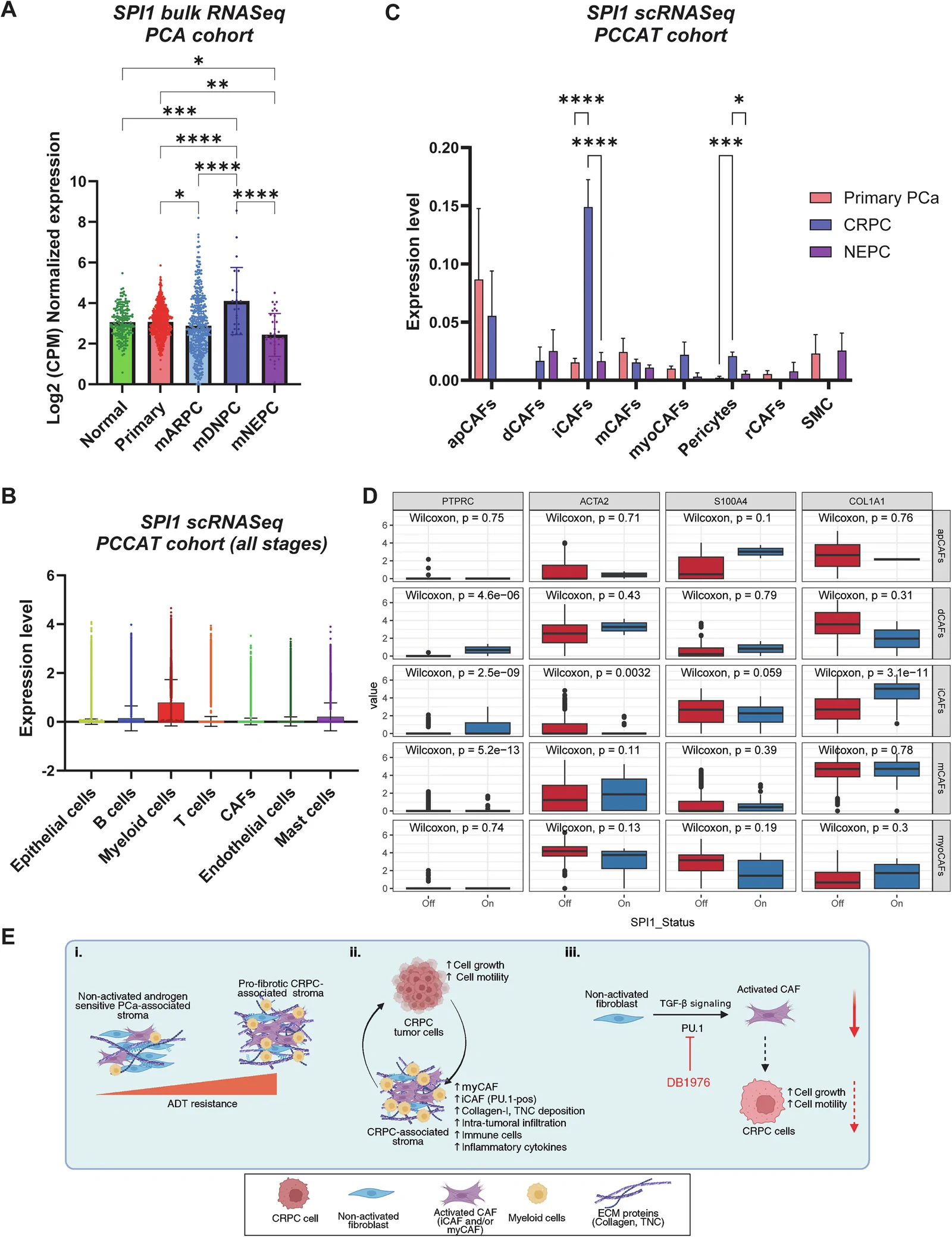
PU.1 (*SPI1*)-mediated formation of a pro-fibrotic CAF stroma is associated with CRPC progression. **A** Bar plots representing *SPI1* gene expression across different PCa stages based on a cross-study comparison of multiple bulk RNA-seq datasets performed through the Prostate Cancer Atlas ^61^. The logarithm of counts per million reads (log CPM) is reported on the y-axis. Mean, standard deviation, and every single measurement (dots) are reported. Ordinary one-way ANOVA with Tukey’s multiple comparisons, *p* < 0.05 (*), *p* < 0.01 (**), *p* < 0.001 (***), *p* < 0.0001 (****). ARPC: AR-positive castration-resistant prostate cancer, DNPC: double-negative prostate cancer, NEPC: neuroendocrine prostate cancer. **B** Bar plots showing human *SPI1* normalized gene expression level across prostate cell types (epithelial, B cells, T-cells, mast cells, myeloid cells, CAFs, and endothelial cells) in all PCa stages. Mean, standard deviation, and every single measurement reported. **C** Bar plots showing human *SPI1* normalized gene expression level across the indicated stromal cell types, comparing its expression in primary PCa, CRPC, and NEPC samples. For visualization purposes, the mean and standard error of the mean with no individual values were plotted. Bar plots with standard deviation and individual values are in Supplementary Fig. 11D. Two-way ANOVA with Šídák’s multiple comparisons test, *p* < 0.05 (*), *p* < 0.01 (**), *p* < 0.001 (***), *p* < 0.0001 (****). **D** Boxplots showing *PTPRC*, *ACTA2*, *S100A4*, and *COL1A1* (columns) normalized expression levels across the indicated cell types (rows) in CRPC samples. Annotated single cells have been stratified for their *SPI1* status; off: no *SPI1* expression detected, on: *SPI1* expression detected. **B**–**D** Data have been obtained from the single-cell RNA sequencing Prostate Cancer Cell Atlas (PCCAT)^62^. apCAF: antigen-presenting CAF, dCAF: differentiated CAF, iCAF: inflammatory-like CAF, mCAF: matrix-associated CAF, myoCAF: myofibroblast-like CAF, rCAF: reticular CAF, SMC: smooth-muscle cells. These subpopulations were defined in ref. ^62^. **E** Graphical summary of the proposed cellular and molecular stromal alterations in CRPC progression. **i** CRPC-associated stroma shows a pro-fibrotic phenotype compared to androgen-sensitive PCa-associated stroma. **ii** CRPC tumor cells induce the formation of a pro-fibrotic stroma enriched with immune cells, iCAF, and myCAF, which produces inflammatory cytokines, and ECM proteins (e.g., collagen-I, TNC). This reactive stroma might, in turn, promote tumor cell motility and invasive potential (intra-tumoral infiltration). **iii** TGFβ-signaling and the transcription factor PU.1 are involved in the acquisition of the reactive stroma phenotype (activated iCAFs and myCAFs), which supports CRPC tumor cell growth and motility (indicated by the black dashed arrow). Pharmacological inhibition of PU.1 transcriptional activity (DB1976 compound) prevents the reactive stromal phenotype induction (full red arrow), thereby indirectly impacting tumor cell growth and motility (dashed red arrow). Legend of the described cellular and non-cellular components is reported at the bottom. Created in BioRender. Karkampouna, S. (2026) https://BioRender.com/vcjuqbx.

To evaluate the clinical relevance of SPI1/PU.1 as a putative therapeutic target, we assessed its gene expression in publicly available human PCa cohorts, including advanced CRPC samples^60–63^. Cross-study comparison on bulk RNA-seq data, performed via the online publicly available Prostate Cancer Atlas database^61^ revealed significantly higher *SPI1* gene expression in metastatic CRPC with respect to primary PCa and normal prostate tissue samples (Supplementary Fig. 11A); while not relevant differences were found in PCa samples stratified according to their primary and secondary Gleason patterns (Supplementary Fig. 11B). More specifically *SPI1* expression was markedly higher in double-negative PCa cases (DNPC), i.e., AR-negative CRPC without a neuroendocrine phenotype, compared to AR-expressing CRPC and neuroendocrine PCa (Fig. 6A). These findings are consistent with our observation that the castration-resistant LAPC9 model does not show neuroendocrine features, but it does exhibit an AR-negative/AR-low phenotype^64^. Similarly, *SPI1* target genes previously defined via the MR analysis (Fig. 4D, E and Supplementary Table 2) were expressed at higher levels in DNPC compared to neuroendocrine CRPC (Supplementary Fig. 11C).

Analysis of the online publicly available single-cell RNA-sequencing Prostate Cancer Cell Atlas (PCCAT)^62,63^ confirmed that *SPI1* expression is mostly associated with cells of the hematopoietic myeloid lineage in human PCa (Fig. 6B) in line with our LAPC9 PDX data. Nonetheless, analysis of *SPI1* gene expression across the stromal cell compartment (including fibroblasts, pericytes, and smooth muscle cells) revealed that *SPI1* expression is enriched in fibroblasts displaying an iCAF phenotype in CRPC, but not in primary and metastatic neuroendocrine PCa samples (Fig. 6C and Supplementary Fig. 11D), in line with the bulk RNA-seq data (Fig. 6A). *SPI1* expression was instead not detected in iCAF from metastatic hormone sensitive PCa (similar to the PNPCa, and BM18 models), probably due to the small sample size of this cancer subtype in the PCCAT dataset. The iCAF subpopulation in CRPC samples also showed expression of the hematopoietic-lineage marker CD45 (encoded by *PTPRC*), FSP1 (encoded by *S100A4*), and collagen type I (*COL1A1*) (Fig. 6D). Moreover, *PTPRC* expression and a higher *COL1A1* expression were associated with positive SPI1 status, i.e. they were detected in those iCAF-annotated cells which also showed *SPI1* expression (Fig. 6D). Overall, these data are in line with our data from the LAPC9 tissue in which PU.1-positive cells express CD45 and possess fibroblast-associated properties; and these observation further imply that PU.1-positive fibroblasts represent a CAF subpopulation interacting with immune cells (iCAF) and/or fibroblasts of hematopoietic lineage origin, (fibrocytes)^52–54^.

In conclusion, analysis of publicly available transcriptomic datasets indicates that PU.1 (*SPI1*) expression is associated with AR-negative non-neuroendocrine PCa (DNPC), and in this setting, it is expressed by CD45-positive iCAF/fibrocytes, mediating the development of a pro-fibrotic and pro-inflammatory stromal phenotype that supports tumor cell growth and invasion. Pharmacological inhibition of PU.1 transcriptional activity (DB1976) could possibly impact CRPC progression (summary in Fig. 6E).

## Discussion

In this study, we aimed to characterize the TME of androgen-sensitive and castration-resistant PDX models of advanced PCa to identify key phenotypic changes occurring in CAFs during tumor progression. In parallel, our goal was to establish PDX-derived tumor-CAF 3D co-cultures that could mimic features of the TME, thus representing valuable in vitro tools to determine the effect of TME-targeting drug compounds. We employed three subcutaneous PDX models representative of androgen-sensitive (PNPCa, BM18) and castration-resistant (LAPC9) stages of advanced PCa, whereby the PNPCa model was obtained from a soft tissue metastasis, and the BM18 and LAPC9 models from bone metastases. Exploiting the different species of origin of tumor (human) and stromal (mouse) cells, PDX transcriptomic analysis revealed that the stromal (mouse-specific reads) gene expression profile differs among the three PDX models in line with previous findings from our lab^28^. Transcriptomic and histological analyses revealed that compared to the androgen-sensitive PNPCa and BM18 models, the TME of the castration-resistant LAPC9 model displays features of a “reactive” stroma, characterized by high collagen and TNC deposition and fibroblasts expressing α-SMA, typical of an MFB-associated phenotype^10^. Moreover, relative to PNPCa and BM18 stroma, LAPC9 stromal cells show higher expression of genes associated with a myCAF state gene signature in primary PCa^65^.

To study how CRPC cells induce the formation of a desmoplastic phenotype, we subcutaneously implanted LAPC9 tumor cells depleted of their original stroma to new mouse hosts to allow the tumor cells to interact with a completely naïve stroma. Histological characterization of LAPC9 tumors collected at different time points revealed a progressive increase in intratumoral collagen and TNC deposition, with the presence of nearly all TNC-negative stroma in the first time point analyzed (week 2). Furthermore, an increase in LAPC9 tumor cell invasive capabilities was observed along with the time of tumor growth, represented by an increased number of tumor-invasive nests. This higher invasive potential correlated with a higher amount of intratumoral TNC and collagen deposition. Altogether, these data suggest that castration-resistant PCa cells induce the formation of a fibrotic stroma, which infiltrates the tumor area, promoting, in turn, cancer cell invasiveness. A potential limitation of the study is the use of PDX models due to the lack of immunological components, which may impact extrapolation to human tumor biology. To establish an in vitro tumor-CAF co-culture model that can mimic features of the parental tumor, we developed collagen-based direct tumor-CAF 3D co-cultures, mimicking cell–cell and cell–ECM interactions. Transcriptomic analysis of PDX-derived fibroblasts grown in standard 2D settings revealed that they do not retain the gene expression profile of the original PDX stroma, suggesting that the absence of interactions with cancer cells and the ECM, adhesion to plastic, and/or cell culture medium composition modify the stromal transcriptomic profile. Therefore, to avoid the growth of stromal cells in 2D, we recombined PDX tumor and PDX stromal cells directly after tumor tissue digestion to generate collagen tumor-CAF 3D co-cultures. In this setting, LAPC9-derived CAFs displayed typical MFB features with higher expression of CAF-related genes such as *Acta2*, *Col1a1*, *Col2a1*, *Col5a1*, and *Mmp9*, compared to PNPCa-derived CAF, similar to what was observed in vivo. More importantly, we determined an increase in cell viability and a decrease in cell apoptosis of androgen-dependent PNPCa tumor cells upon co-culturing with LAPC9-derived CAFs, suggesting that CAFs derived from the castration-resistant LAPC9 model possess pro-tumorigenic properties. Overall, these data suggest that collagen-based tumor-CAF 3D co-cultures are able to recapitulate, at least partially, features of the in vivo TME differently from 2D-cultured fibroblasts.

After establishing a suitable in vitro co-culture model, we sought to investigate the molecular mechanisms that drive the LAPC9 pro-fibrotic phenotype. Analysis of the MRs driving the DEG profile observed in the stroma of the three PDX models identified several transcription factors to show higher activity in the LAPC9 versus the PNPCa and versus the BM18 stroma, some of which, such as *Nfkb1*, *Jun*, *Sp1*, *Ets1*, *Stat3*, and *Spi1* have been reported to play a role in tissue fibrosis and the TGFβ-dependent induction of MFB phenotype^42–47^. Among these genes, *Spi1*, coding for the transcription factor PU.1, represented a good candidate for further investigation since a selective small molecule inhibitor (DB1976) that specifically inhibits its binding to the DNA exists^49^, and it has been demonstrated that DB1976 is able to revert MFBs into a resting non-activated phenotype in the context of tissue fibrosis^47^. To the best of our knowledge, the pro-fibrotic role of PU.1 in solid cancers has not yet been investigated.

PU.1 is known to be expressed not only by fibroblasts but also by cells of hematopoietic origin (CD45-positive), in which it plays fundamental roles in monocyte and B-cell differentiation^50^. Western Blot analysis of FACS-isolated LAPC9 tumor cells, immune/hematopoietic cells (CD45-positive), and remaining stromal cells (CD45-negative) revealed that PU.1 is specifically expressed in the CD45-positive hematopoietic/immune cell fraction. These cells could be cells of myeloid origin (given that *CB17-SCID* mice lack lymphocytes), such as monocytes or macrophages. Nonetheless, a subset of PU.1-positive cells possesses fibroblast properties and, thus, it is likely to contribute to the LAPC9 stroma pro-fibrotic phenotype, given the presence of PU.1-expressing cells being positive for the fibroblast marker FSP-1 and localizing in α-SMA, collagen, and TNC-positive areas. These cells might be defined as fibrocytes, i.e., cells of hematopoietic origin that secrete collagen and display fibroblast features^53^. Our observation is in line with studies reporting the presence of CD45+/FSP1+, CD45+/FAP+, and CD45+/Col1+ fibroblast-like cells^52,54,59^, and with a study identifying *SPI1* as a transcription factor associated with fibrocyte-like apCAFs^55^. FSP1, co-expressed with PU.1 in a subset of PU.1-positive cells in the LAPC9 stroma, has been found to be upregulated in macrophage-derived MFBs during their differentiation (monocyte/macrophage-myofibroblast transition or MMT)^66^. These PU.1-positive myeloid cells may also contribute to the CRPC-associated pro-fibrotic phenotype, given the localization of PU.1-positive cells in TNC and collagen-positive areas and that a more inflamed TME represents a feature of the “reactive” stroma^67,68^. M2-type macrophages are also able to induce a pro-fibrotic phenotype in fibroblasts in a PU.1/mTOR-dependent manner in tissue repair after stroke^69^, highlighting the role of PU.1 in the crosstalk between fibroblast-macrophages. Moreover, expression of PU.1 in combination with the C/EBPα/β transcription factor in mouse embryonic fibroblasts (3T3 cells) induces the conversion of fibroblasts into CD45-positive macrophage-like cells^70^, highlighting the possibility that PU.1-expressing cells might interconvert between a fibroblast-like and a macrophage-like state (MMT). Finally, analysis of the single-cell RNA-seq (scRNA-seq) Prostate Cancer Cell Atlas (PCCAT)^62,63^, revealed that *SPI1* is specifically enriched in *PTPRC* (CD45) and *S100A4* (FSP1)-expressing iCAFs in the CRPC setting, but not in primary or neuroendocrine PCa. Overall, these data suggest that PU.1 (coded by *SPI1*) is a transcription factor enriched in CRPC and expressed by a subset of cells of hematopoietic origin, which possess fibroblast properties (fibrocytes), mediate inflammatory responses (iCAFs, apCAFs), and/or might interconvert into macrophages (MMT). The scRNA-seq data indicate that the findings from the stroma of the PDX models are translatable to the stroma marker expression and composition seen in human PCa tumors. After validating the stromal-specific expression of PU.1 in the LAPC9 tissue and its localization in reactive stromal areas (TNC- and αSMA-positive), we sought to determine whether PU.1 blockade might inhibit the LAPC9 pro-fibrotic phenotype, consequently impacting tumor cell growth. For this purpose, LAPC9 tissue-derived tumor-CAF collagen co-cultures were treated with the PU.1 inhibitor DB1976 for two weeks starting from the day of seeding. After two weeks of treatment, we observed a significant reduction in the amount of phalloidin-stained MFB actin fibers and the formation of smaller tumor organoids compared to the vehicle. To confirm the specific CAF-targeting effect, we recombined in collagen co-cultures LAPC9 tumor cells, depleted of their original stroma, with mouse dermal fibroblasts (MDFs), determining that the organoid size of LAPC9 tumor + MDF co-cultures was higher than the LAPC9 tumor cell monoculture control, but upon DB1976 treatment, the organoid size in co-culture was comparable to the monoculture control. Upon PU.1 inhibition, we also observed a reduced tendency of LAPC9 tumor organoids to localize toward the edges of the collagen plug, suggesting lower tumor cell motility, in line with our in vivo data showing that the presence of an intratumoral TNC-positive stroma promotes tumor cell invasiveness.

Analysis of publicly available bulk RNA-seq datasets from different stages of human PCa revealed higher expression of *SPI1* and its target gene expression in AR-negative and non-neuroendocrine PCa samples, showing similar features to the LAPC9 CRPC model (AR-negative/AR-low and non-neuroendocrine)^64^, thus enhancing the clinical translatability of our findings.

In this study, we also acknowledge potential limitations. Due to the relatively small number of biological replicates for some of the experiments performed, additional experimental validation could further support our findings. Furthermore, despite their advantages compared to simpler co-culture methods (e.g., indirect and/or 2D co-cultures), we acknowledge the possibility that tumor-CAF collagen 3D co-cultures might not fully recapitulate the complexity of the in vivo TME. Further investigations are required to fully understand the molecular mechanisms of PU.1-mediated fibrosis in CRPC, particularly the role of PU.1 in the crosstalk between fibroblasts and immune cells. To shed light on these mechanisms, the effect of PU.1 inhibition should also be studied in vivo or by using ex-vivo tissue slices^71^ that retain the parental tumor tissue architecture.

Our findings suggest that the CRPC-associated stroma, differently from the androgen-dependent PCa-associated stroma, shows pro-fibrotic and pro-inflammatory features, with a higher abundance of myCAF, myeloid cells/macrophages, and high ECM protein deposition (collagen and TNC). These stromal cells acquire an intra-tumoral localization, possibly increasing the tumor cell invasive potential. The PU.1 transcription factor (encoded by the *SPI1* gene) is a key orchestrator of the pro-fibrotic and pro-inflammatory “reactive” stromal phenotype. Pharmacological blockade of its transcriptional activity could potentially revert this phenotype, impacting tumor cell growth.

In conclusion, this study paves the way for further investigations on the CRPC-associated pro-fibrotic phenotype and on combinatorial treatment approaches, simultaneously targeting CRPC cells and their TME, highlighting the applicability of tumor-CAF 3D co-cultures as tools to pursue these objectives.

## Materials and methods

### Animal experiments

All animal experiments were approved by the ethical committee of Canton Bern (animal license BE 67/20 and BE103/23). At humane endpoint (tumor size = 1 cm^3^), mice were euthanized, and the PDX tumor tissue was collected, and a part of it was implanted subcutaneously in new male *CB17-SCID* immunodeficient mice (8-10 week- old) under anesthesia (Domitor® 0.5 mg/kg, Dormicum 5 mg/kg, Fentanyl 0.05 mg/kg). After surgery had been completed, an antidote was given (Alzane 1.1 mg/kg, Anexate 0.45 mg/kg, Temgesic 0.075 mg/kg), and mice were monitored post-surgery. Tumor growth (palpation) and body weight were assessed weekly. For reporting of animal experiments, relative to Fig. 2, the ARRIVE guidelines have been used. The groups were allocated based on time from LAPC9 tumor implantation in weeks 2, 3, 4, and 5 to assess the kinetics of stroma infiltration. The week 5 group represents the fully formed tumor condition (control group). The experimental unit is a single animal within which *n* = 2 tumors were implanted, each for a separate downstream analysis (histology, RNA expression). The group size was *N* = 3 animals per time point, with a total of 12 animals and 24 tumor tissues. The sample size was selected on the basis of LAPC9 tumor kinetics, the stability of the tumor model among different tissues/animals, and prior observations on three distinct biological tumors yielding statistically significant results. No a priori inclusion or exclusion criteria were set. At downstream analyses, the tumor samples used for RNA isolation were omitted due to the low amount of stromal transcriptome at the early stages of tumor formation. An additional time point (Week 4) was excluded during quality control of the histomorphology of the tumors. The number of experimental units is reported in each Figure legend. Experimental units were randomized based on body weight differences and across different cages to avoid confounder effects. Outcome measures used were tumor formation, tumor proliferation, stromal infiltration, and stromal molecular markers. All animals were housed under regular light and dark cycles and on a standard chow diet. Body conditioning score and tumor growth were the human endpoints for this study. To minimize distress and aggression, additional nourishment materials were added to the cages.

### PDX tissue digestion and organoid derivation

PNPCa, BM18, and LAPC9 subcutaneous PDX tumors were collected during serial passaging. Mechanical and enzymatic dissociation were used to obtain a single cell suspension. Enzymatic digestion occurred through 1–2 h incubation with Collagenase Type-II 5 mg/ml (Gibco) at 37 °C, followed by incubation of 20-30 min with TrypLE (ThermoFisher). PDX-derived human and mouse single cells were separated through MACS by using a Mouse Cell Depletion Kit (Miltenyi Biotec, 130-104-694). PDX-derived human (tumor) or recombined (tumor+stroma) cells were seeded in 6-well, 24-well, or 96-well ULA plates (Corning) in PCa organoid medium and cultured at 37 °C, 5% CO_2_ to allow organoid formation (3–8 days).

PCa organoid medium consists of DMEM/F12 (Gibco) supplemented with 1% GlutaMAX (Gibco), Hepes 10 mM, Primocin 1:500 (inVivoGen), Fetal Bovin Serum (FBS) 5% (Gibco), B27 Supplement 2% (Thermo Fisher), Nicotinamide 10 mM, R-spondin 500 ng/ml, N-acetyl-cysteine 1.25 mM, SB202190 10 μM, Noggin 100 ng/ml, Y-27632 (Rock Inhibitor) 10 μM, Dihydrotestosterone (DHT) 1 nM, Wnt3A 10 ng/ml, HGF 50 ng/ml, A83-01 (TGF-ß inhibitor) 500 nM, EGF 50 ng/ml, FGF10 10 ng/ml, FGF2 1 ng/ml, and PGE2 1 μM. Rock inhibitor was added only in the initial days to allow organoid formation, but not for medium refresh.

### Fibroblast culture

MDF, PDX-derived fibroblasts, and patient-derived primary PCa CAF were grown in Dulbecco’s modified Eagle Medium (DMEM) GlutaMAX (Gibco) supplemented with 10% FBS, 1% Penicillin-Streptomycin (Life Technologies). For MDF and PDX-derived fibroblasts, the medium was additionally supplemented with 1% Insulin Transferrin Selenium (ThermoFisher) and FGF2 10 ng/ml (referred to as fibroblast medium). PDX-derived fibroblasts were obtained by seeding the PDX MACS-purified mouse fraction in regular cell culture Flasks. MDF was purchased by ScienceCell (Cat# M2300-57; Lot#17791). Patient-derived primary PCa CAF have been received from our collaborator (N.S.). For MFB phenotype induction, primary prostate CAF and MDF were treated with TGF-β1 5 ng/ml for 5 days. Fibroblasts were serum-starved (0.3% FBS) for 24 h before TGF-β1 treatment initiation.

### Tumor-CAF 3D collagen co-cultures

For direct collagen-based tumor-CAF 3D co-cultures, PDX tissue-digested tumor and stromal cells were cultured in ULA plates (Corning) to allow organoid formation (density of 0.5−0.8 million cells per well of a 6-well ULA plate). Once formed, organoids were transferred in 20 μl of rat collagen 3 mg/ml solution (R&D Systems, 3447-020-01) in regular 24-well tissue culture plates. The gelling procedure was performed by using NaOH for pH neutralization, followed by incubation for 30–40 min at 37 °C. Subsequently, a 1:1 ratio of PCa organoid medium and fibroblast medium was added. The fibroblast medium was supplemented with DHT 1 nM, and the PCa organoid medium was prepared without A83-01 (TGF-ß inhibitor).

For the generation of hybrid PNPCa tumor- LAPC9 CAF co-culture establishment, MACS-purified tumor and stromal cells were recombined at a 4:1 to 6:1 ratio, depending on the original PNPCa tumor/stromal cell ratio obtained after tumor tissue digestion. MACS-purified LAPC9 stromal cells underwent a second round of FACS-mediated purification to avoid recombination of LAPC9 tumor cells (GFP-positive) in the hybrid co-culture. To effectively distinguish unlabeled tumor and stromal cells, PNPCa live tumor cells were incubated for 20 min at 37 °C with BioTracker™ 555 Orange Cytoplasmic Membrane Dye (Millipore, SCT107) before recombination.

For LAPC9 tumor-CAF co-culture generation, PDX-derived tumor (GFP-labeled) and stromal cells were seeded directly as single cells in a collagen 3 mg/ml solution with a final cell density of 50.000 cells per 20 μl plug to allow organoid formation. LAPC9 tumor cells (MACS-depleted of their original stroma) were also recombined in a 3:2 ratio with MDFs as single cells in collagen for a total cell amount of 50.000 cells per collagen plug. DB1976 treatment at the 1 μM, 2.5 μM, 5 μM, and 10 μM doses was performed for two weeks starting from the day of cell seeding in the presence (DHT 1 nM) or absence of DHT. In addition, LAPC9 tumor-CAF co-cultures were generated by allowing tumor organoid formation in ULA and subsequently transferring the organoids to collagen for stromal cell adhesion. In this setting, DB1976 treatment was performed for seven days after tumor-CAF co-cultures had formed in collagen.

For all co-culture experiments, at the endpoint, the collagen 3D co-cultures were fixed with 4% PFA for histological analysis, lysed in TriPure for RNA extraction, or digested with Collagenase Type-II and TrypLE to obtain a single-cell suspension for flow cytometry analysis.

### Generation of GFP-Luciferase-labeled PDX models

For the generation of GFP-Luciferase labeled LAPC9 PDX models, MACS-purified LAPC9 human (tumor) single cells were seeded in collagen-coated plates (rat collagen 0.3 mg/ml, R&D Systems, 3447-020-01) and transduced with GFP-Luc lentiviral vectors (ATG-2460; GFP-click beetle luciferase) at a multiplicity of infection (MOI) of 1. After 2 days, LAPC9-GFP-Luc tumor cells were transferred to 3D culture conditions to allow organoid formation. Once formed, organoids were resuspended in Matrigel (Corning) and subcutaneously injected into mice (under anesthesia). Tumor growth was monitored through the IVIS Imaging System (PerkinElmer) following intraperitoneal injection of D-sodium salt luciferin (150 mg/kg). To deplete potential non-transduced tumor cells, the newly generated LAPC9-GFP-Luc tumors were collected at the endpoint, digested, and FACS was performed to select the GFP+ cells. FACS-purified GFP+ cells were then re-injected subcutaneously into new mouse hosts.

### Bulk RNA-sequencing and barcoding (BRB-seq) data analysis

RNA was extracted from PDX tissue-derived stromal (mouse) fraction cells and PDX-fibroblasts lysed in TriPure Isolation Reagent (Sigma-Aldrich). RNA extraction was performed using the Direct-zol RNA Microprep kit (Zymo Research). RNA concentration was measured with NanoDrop™ 2000/2000c Spectrophotometer and with Qubit^TM^ RNA Broad Range (BR) Assay Kit (ThermoFisher). RNA quality was assessed through the Agilent Total RNA 6000 Nano Kit (Bioanalyzer) or with the Qubit™ RNA IQ Assay Kits (Thermo Fisher). RNA samples with an amount > 200 ng and RNA integrity number >6 were sent for sequencing. Samples were sent to Alithea Genomics (Lausanne, Switzerland) for sequencing using the BRB-seq method^38^. fastQC (v0.11.7) was used to assess the quality of sequencing reads. Sequencing reads were aligned to either the human genome (GRCh38, Ensembl 102) or the mouse genome (GRCm38, Ensembl 102) with STAR361 (v2.7.10a).

Downstream analyses were conducted using R (v4.4.2). Deduplicated unique molecular identifiers (umi) count matrices were used to create the initial DESeq2 object^72^ (v1.46.0). Genes with fewer than 3 counts in fewer than 3 samples were removed.

Differential expression analysis was performed using DESeq2 with the Wald method. Shrinkage of fold changes was applied using the “normal” method as specified in the DESeq package. Genes were considered differentially expressed if they had an adjusted *p*-value below 0.05 and an absolute shrunken fold change greater than 2.

Enrichment analysis was conducted using the clusterProfiler package^73^ (v4.14.4) with the compareCluster function and the “enrichGO” method using the gene ontology biological processing gene sets. MR inference was performed using the decoupleR package^41^ (v2.12.0) with the decouple function, utilizing the collecTRI mouse regulon^74^. Subsequently, the different scoring methods were combined using the run_consensus function from decoupleR.

### H&E staining

Tissues were fixed in 4% paraformaldehyde (PFA) and then dehydrated in increasing concentrations of ethanol (from 50% to 100%) for paraffin embedding. After paraffin embedding, tissue slices were cut (5 μm tissue slices), and de-paraffinization was performed by transferring the tissue slices in xylol, decreasing concentrations of Ethanol (from 100% to 70%), and distilled H_2_O. Tissues were then incubated with Hematoxylin (blue, marking cell nuclei) and Eosin (pink, marking basic cytoplasmic and extracellular matrix proteins). Tissues were then incubated with increased Ethanol concentrations and xylol and finally mounted with Entellan®/Eukitt® Mounting medium (Sigma Aldrich). Images were acquired through the Panoramic 250 Flash II Slide Scanner (3DHistech).

### Immunofluorescence staining

Immunofluorescence staining was performed on FFPE PDX tissue sections and collagen-based tumor-CAF 3D co-cultures. Tissues were fixed in 4% PFA and dehydrated in increasing ethanol concentrations for paraffin embedding. 5 μm tissue slices were cut, and deparaffinization was performed. After deparaffinization, antigen retrieval was performed with Citrate Buffer (Vector Laboratories) for 10–15 min at pH = 6. Blocking was performed with 1% bovine serum albumin (BSA) dissolved in PBS 1X + 0.1% Tween. Primary antibodies were incubated overnight at 4 °C diluted in a blocking solution (list of primary antibodies used reported in Supplementary Table 3). Secondary antibodies were incubated at room temperature for 90 min diluted in PBS 1X + 0.1% Tween at a 1:250 dilution (list of secondary antibodies used reported in Supplementary Table 3). For cell nuclei detection, DAPI or TO-PRO-3 were incubated for 10–15 min in PBS 1X (1:500 or 1:1000 dilution, respectively) or with secondary antibodies (only for DAPI). Slides were mounted with ProLong Diamond Antifade (ThermoFisher). Images were acquired through the CQ1 Confocal Microscope (Cenibra GmbH) or through the Panoramic 250 Flash II Slide Scanner (3DHistech).

### Whole-mount staining

Whole-mount staining was performed on 2D- and 3D-cultured cells. For whole-mount IF staining, the samples were fixed in 4% PFA and permeabilized in 0.3% TritonX100/PBS 1X. Blocking was performed in 10% serum (same species of secondary antibodies) in PBS 1X + 0.05% Tween (or 0.05% Triton). Primary antibodies were incubated for 3 h at room temperature or overnight at 4 °C, diluted in blocking solution (list of primary antibodies used reported in Supplementary Table 3). Secondary antibodies were incubated at room temperature for 90 min diluted in PBS 1X at a 1:250 dilution. Phalloidin-iFluor555 Reagent (Abcam), DAPI, or TO-PRO-3 were incubated for 10–15 min in PBS 1X or with secondary antibodies (only for Phalloidin and DAPI). 2D cultured cells were cultured and stained in Polystyrene Vessel Tissue Culture Treated Glass Slides (Falcon). After the staining procedure was completed, the polystyrene vessel was removed, and the slides were mounted with ProLong Diamond Antifade (ThermoFisher). 3D cultured cells were stained in standard cell-culture plates and kept in PBS 1X.

For live cell staining (collagen tumor-CAF 3D co-cultures), Hoechst3342 at 1:5000 or PI at 1:200 dilutions were directly added to the cell culture medium.

Images were acquired using the CQ1 Confocal Microscope (Cenibra GmbH).

### IHC staining and double IHC staining

IHC and double IHC staining were performed at the Translational Research Unit (TRU) at the Institute of Tissue Medicine and Pathology, University of Bern. For single IHC staining (Ki67), antigen retrieval was performed in Tris EDTA for 30 min at 95 °C. The BOND RX staining platform was used, with signal detection with BOND polymer refine DAB (#DS9800, Biosystems).

For double IHC staining (PU.1, CD45, FSP-1), antigen retrieval was performed in Citrate buffer for 30 min at 100 °C. The BOND RX staining platform was used, with signal detection with BOND polymer refine DAB (#DS9800, Biosystems) and BOND polymer refine RED (#DS9390, Biosystems). Hematoxylin counterstaining is included in the staining platform. Slides were mounted with Pertex™ mounting medium (Biosystems). Double-IHC quantification analyses were performed with the Visiopharm software (v2023.09×64 Hørsholm, Denmark) and evaluated by an expert pathologist (S.d.B).

### Pathological assessment of tumor-stroma infiltration in vivo experiment

MACS-purified LAPC9-GFP-Luc human (tumor) single cells were implanted subcutaneously in Matrigel plugs. Tumor growth was monitored by palpation and weekly through the IVIS Imaging system. Tumors were collected for histological analysis at the following time points: week 2, week 3, and week 5. H&E staining, Ki67 IHC, and IF staining for stromal marker expression were performed. Blinded histopathological assessment and quantification of the stainings were performed by an expert pathologist (S.d.B.). To evaluate the presence of invasive tumor nests, Ki-67 stained tissue sections were used, as the strongly Ki-67–positive tumor tissue was morphologically clearly distinguishable from reactive stroma and non-neoplastic epithelial structures. Invasive nests were manually outlined by a pathologist, and the number and area of individual invasive nests were measured. Quantification of stromal marker expression (TNC, collagen) was performed with the Visiopharm software (v2023.09×64 Hørsholm, Denmark). The following workflow was applied: 1. Tissue segmentation and definition of region of interest (ROI), i.e., tumor versus stromal areas; 2. manual ROI correction; 3. manual definition of intra and peri-tumoral stromal areas; 4. detection of stromal TNC and collagen-positive tissue areas and their combination using a threshold classifier; 5. manual correction of non-specific labels (artifacts). The annotated intra- and peri-tumoral stromal areas were normalized for the total (peri+intratumoral) stromal area for each sample.

### RNA isolation and RT-qPCR

Total RNA was isolated with TriPure Isolation Reagent (Sigma-Aldrich). 0.2 ml of Chloroform was added per 1 ml of TriPure to separate the aqueous phase containing RNA. RNA was precipitated with isopropanol (0.5 ml per 1 ml of TriPure) and suspended in RNAse-free water. Alternatively, RNA was isolated using the Direct-zol RNA Microprep kit (Zymo Research). RNA concentration was measured with NanoDrop™ 2000/2000c Spectrophotometer or with Qubit^TM^ RNA Broad Range (BR) Assay Kit (ThermoFisher). cDNA was obtained by using M-MLV reverse transcriptase 200U/reaction (Promega) enzyme. qPCR was performed with FastStart Universal SYBR Green Master (Sigma) in MicroAmp™ Fast Optical 96-Well or 384-Well Reaction Plate (ThermoFisher) with a cDNA concentration of 1 ng/μl in 10 μl reaction volume per well. Forward and reverse mouse gene-specific primers were added at a concentration of 250 nM per well (list of primers used reported in Supplementary Table 4).

### Protein isolation and Western Blot

Proteins were isolated with RIPA buffer (Tris-HCl pH = 8 50 nM, NaCl 150 nM, Triton-X100 1%, Na-deoxycholate 0.5%, SDS 0.1%, EDTA 5 nM) plus Protease Inhibitors and Phosphatase Inhibitors. Protein concentration was obtained using the Pierce™ BCA Protein Assay Kit by reading 562 nm absorbance with Tecan Infinite M200 Pro. 4X Laemmli Loading Buffer (BioRad) with 10% ß-mercaptoethanol was added to protein lysates to obtain a final concentration of Loading Buffer 1X. SDS-page was performed with Mini-PROTEAN® Precast Gels (BioRad). Proteins were transferred to iBlot Transfer Regular nitrocellulose Stacks (ThermoFisher) by using iBlot 2 Dry Blotting System (ThermoFisher). Membrane blocking was performed with 5% BSA in TBS-Tween (TBS-T) 0.1%. Primary antibodies were diluted in 5% BSA in TBS- T 0.1%, while Horseradish peroxidase (HRP)-conjugated secondary antibodies in TBS-T 0.1% (list of antibodies used reported in Supplementary Table 3).

WesternBright ECL HRP substrate (Advansta) was used for membrane development. Signal was detected with the Gel Documentation system Fusion-FX7-820. The protein band quantification was performed with the software Fiji (ImageJ) and normalized to the loading control protein. Uncropped Western Blot images with size markers are reported in the Supplementary Information file.

### Flow cytometry

The obtained single-cell suspensions were resuspended in sterile PBS with 0.5% BSA and EDTA 2 mM (FACS Buffer) and filtered through 30–40 μm strainers. Mouse Fc-receptor blocking was performed at room temperature for 5 min with FcR Blocking Reagent (Miltenyi Biotec, 130-092-575). Fluorophore-conjugated antibodies were diluted in FACS Buffer in a 100 μl volume of cell suspension and incubated for 15–20 min at room temperature (list of antibodies used reported in Supplementary Table 3).

Washing was performed, and cells were resuspended at a density of 5–10mln/ml in FACS Buffer with DAPI or TO-PRO-3 0.1 μg/ml and kept on ice. For Annexin V staining (Biolegend, 640914), antibodies were incubated in Annexin V Binding Buffer (BD Pharmigen), containing calcium and magnesium. Flow cytometry and FACS acquisition were performed at the Flow Cytometry and Cell Sorting Core Facility (FCCS) at the Department for BioMedical Research, University of Bern. Flow cytometry was performed with a BD FACS SORP LSR II instrument. For FACS, the MoFlo ASTRIOS Eq or the BD FACSDiscover S8 with CellView instruments were used. After sorting, cells were collected in FBS. Data analysis (including gating and compensation) was performed using the FlowJo software (BD Biosciences).

### Organoid diameter and area measurement

Tumor organoid diameter and average area size were obtained using the Fiji (ImageJ, version 1.54p) Software Fiji. The diameter of tumor organoids in collagen tumor-stroma 3D co-cultures was manually measured (μm) using brightfield microscope images, in which it was possible to clearly distinguish tumor organoids and fibroblasts. Diameter measures across images were collected, and plots and statistics were generated. For tumor organoid area quantification in collagen tumor-stroma 3D co-cultures, a threshold was applied to 8-bit (black and white) brightfield and/or phalloidin- staining F-actin (red channel) images. The particle count function was used to identify spherical-shaped organoids by keeping the same particle analysis setting throughout all the measurements. An average organoid area size (μm^2^) was determined per image analyzed. Plot and statistics were generated.

### Nuclear/cytoplasmic PU.1 quantification analysis

To quantify the amount of nuclear/cytoplasmic PU.1 signal based on immunofluorescence analysis, the Fiji software was used. A threshold was applied to 8-bit blue (DAPI) channel images, and the particle count function was used to count the total cell nuclei per image. The particles generated were saved as a region of interest (ROI) through the ROI manager tool. Afterwards, a threshold was applied to 8-bit far red (PU.1) channel images (same threshold for all images), and binary (black/white based on threshold) images were exported. The amount of PU.1-positive (white) area was measured. Nuclear particles (ROI) were loaded to the binary images, and the amount of PU.1-positive area within the nuclei was measured. The percentage of PU.1 nuclear (within ROI) and cytoplasmic (outside ROI) area over the total PU.1 area was calculated per image. Plots were generated.

### Statistical analysis and reproducibility

For analysis, plot production, and statistical tests from the generated BRB-seq datasets, the R programming language (v4.4.2) was used. Statistical significance in gene expression was calculated via the Wald test with Benjamini–Hochberg multiple test correction.

For gene expression (RT-qPCR, and publicly available datasets^61,62^) and general quantification analysis (e.g., average organoid size, % positive cells), bar plots reporting the mean, standard deviation, and each individual value were plotted using the GraphPad Prism software (v10.6.0). In Fig. 6C, no individual values were plotted for visualization purposes, while the same plot with individual values is reported in Supplementary Fig. 11D.

Statistical analyses were also performed with GraphPad Prism. To compare differences among conditions according to one variable (e.g., gene expression among different co-culture conditions), One-way ANOVA was performed when *N* > 3. For One-way ANOVA, Dunnett’s multiple comparison was used when comparing all the conditions to a specific control condition, Tukey’s multiple comparison was used when comparing all the conditions among each other. In Figs. 3I, J and 4G, non-parametric tests (Friedman test for Fig.3I, J for paired data, and Kruskal-Wallis for Fig.4G for unpaired data) with Dunn’s multiple comparison were performed due to small sample size (*n* = 3 measurement/biological replicates per condition) and to bounded data which do not follow a normal distribution (percentage values). To compare differences among conditions according to two variables, Two-way ANOVA with Šídák’s or Tukey multiple comparisons test (*N* > 2) was performed. For each experiment, at least *n* = 3 technical replicates and at least *n* = 2 biological replicates were used. The numerical data sources for all the plots generated are reported in the Supplementary Data file.

### Ethics approval and consent to participate

All animal experiments were approved by the ethical committee of Canton Bern (animal license BE 67/20 and BE103/23).

### Reporting summary

Further information on research design is available in the Nature Portfolio Reporting Summary linked to this article.

## Supplementary information

Transparent Peer Review File

Supplementary Information

Description of Additional Supplementary Files

Supplementary Data

Reporting Summary

## Data Availability

All materials can be made available upon request to the corresponding authors. The RNA sequencing data generated in this study are available through the Gene Expression Omnibus (GEO) with accession number GSE296466. The RNA sequencing data related to Supplementary Fig. 5A is generated by a previous study and are available at EGA under EGAS00001004770. The scRNA-seq gene expression data necessary for the plots in Fig.6 and Supplementary Fig. 11, has been kindly provided prior to peer-reviewed publication by the Xia Lab (Oregon Health & Science University, USA) (publicly available dataset PCCAT. https://pccat.net/ and pre-print version^63^) and they are available in the Supplementary Data file.
