# Supplementary material for: PU.1 is a mediator of the reactive stromal response associated with castration-resistant prostate cancer
Source: Commun Biol. 2026 May 29;9:1174. doi: 10.1038/s42003-026-10297-7 (PMC13547233; doi:10.1038/s42003-026-10297-7)
Supplement: Supplementary file 1 [file 42003_2026_10297_MOESM1_ESM.pdf]

nature portfolio

Peer Review File

PU.1 is a mediator of the reactive stromal response associated with castration-resistant prostate cancer

Corresponding Author: Dr Sofia Karkampouna

This file contains all reviewer reports in order by version, followed by all author rebuttals in order by version.

Version 0:

Reviewer comments:

Reviewer #1

(Remarks to the Author)
This manuscript "PU.1 is a mediator of the reactive stromal response associated with castration-1 resistant prostate cancer" used in vitro collagen-based 3D tumor-CAF organoid co-culture system to study the role of a pro-fibrotic stroma in the castration-resistant (LAPC9) versus the castration-sensitive (PNPCa, BM18) models. The authors identified the transcription factor PU.1 as a mediator of the LAPC9 pro-fibrotic phenotype and showed that pharmacological inhibition of PU.1 reduced tumor organoid growth. In general, the authors used different approaches to address their conclusions. However, the authors may wish to address the following points:

1. Figure 2G-F, it might be important to see if there is a correlation between tumor invasion and stroma populations in PNPCa and BM18 models like LAPC9 model?

2. In LAPC9, does the high fibrotic, myofibroblast-rich stroma contribute to castration resistance phenotype? Would targeting those fibroblasts sensitize LAPC9 to castration?

3. It would be important to include LAPC9 and LAPC9 + LAPC9 CAFs , LAPC9 + PNPCa CAFs organoids in Figure 3D to determine the impact of matched or unmatched CAFs on LAPC9 organoid size.

4. Figure 3 E-I, it would be important to include an additional control showing the expression of CAF associated genes in LAPC9 CAF without coculture with PNPCa cells to know the basal line of those CAF associated genes and to determine if the upregulation of those genes require being the co-culture with PNPCa.

5. Figure 4C and line 349, the authors mentioned that "This confirmed enrichment of Spi1 itself and of its targets; C-X-C motif 349 chemokine ligand 1 (Cxcl1), Acta2, Col3a1, thrombospondin-1 (Thbs1), C-X-C motif chemokine ligand (Cxcl2), in the LAPC9 versus PNPCa and BM18 stroma". However, the data in Figure 4C showed that SPi1 and and Col3a1 are not significantly enriched in LAPC9 vs PNPCa. the authors may wish to consider rewriting that paragraph to reflect only significant changes.

6. Sup. Fig. 5C, the authors should consider running a statistical analysis on this data.

7. Figure 4H-J, it is less clear how the authors determine whether CD45+ cells possess fibroblast properties with these experiments (line 376). While 13.9% of fibroblasts are PU.1+ FSP1+, it is less clear how a subset of PU.1 and CD45 double-positive cells display clear fibroblast properties (line 379-380)? The authors may wish to include staining of CD45 marker to see if that is colocalize with PU.1 and fibroblast marker.

8. Line 416-417 PU.1 is not stromal-specific transcription factor. It is expressed by various immune cells. the authors should consider redrafting that.

9. It is beneficial to include in vivo data to determine the role of PU.1 in fibroblast activity and tumor growth using PU.1 conditional mutant mice with specific deletion in fibroblasts. however, we understand that generation of those mice might take longer time.

Reviewer #2

(Remarks to the Author)
This manuscript investigates phenotypic and molecular differences between androgen-dependent (PNPCa, BM18) and CRPC-associated (LAPC9) prostate cancer stroma using pre-clinical models. It is shown that CRPC stroma adopts a more myofibroblastic profile with greater capacity to stimulate tumor epithelial growth, and the authors identify PU.1 as targetable transcription factor driving this protumorigenic activity.

Overall, the manuscript is well written and data are generally convincing and relevant to the field. Addressing the statistical issues, refining the normalization and clinical dataset analyses, and clarifying some experimental interpretations will significantly strengthen the manuscript.

Major Comments

1. Statistical analysis
The statistical analysis needs careful revision. The authors should provide a full description in Materials and Methods, ensure that the graphical representations match the type of comparisons and inferences, and report appropriate statistical tests for all quantitative analyses. Specifically:

1.1 The use of R^2 from linear regression to infer correlation strength and direction is inappropriate. The description and conclusions should be adjusted to match regression output, or, preferably, a correlation test (Pearson or Spearman) should be performed, reporting both the coefficient (r) and p value.

1.2 Figure 2B: The data appear to come from two replicates with repeated measurements (tumor area in mm^2) over time, representing paired data. This should be plotted and tested accordingly (e.g., points connected by lines), while also showing the three replicates per time point as stated in the legend. Please clarify whether n = 2 or 3.

1.3 Figures 2D–F: Since the replicates are independent, bar plots (mean ± SEM) would be more appropriate than scatter plots. Again, clarify whether n = 2 or 3.

1.4 Figures 3K–L: The normalization to the PNPCa group is not correctly applied. The mean of the three PNPCa replicates should be set to 1, preserving replicate variance, rather than assigning “1” to each replicate individually.

2. Ki67 and microinvasion
The prediction of microinvasion from Ki67+ cell distribution overlooks proliferating CAFs. A more robust approach would involve a human-specific epithelial marker (without mouse cross-reactivity) or one known to label LAPC9 epithelial cells (e.g., PSA, CK8).

3. Hedgehog signaling
The suggestion that Hedgehog signaling activity is more restrained in PNPCa and BM18 compared to LAPC9 (based on Gli1–3 levels in Fig. 4A–B and S5) remains speculative. Since Gli repressors can have opposing effects, stronger evidence is needed. If protein-level data (full-length vs. repressor forms of Gli) cannot be obtained, the RNAseq dataset should be interrogated for Gli target genes (activators and repressors) to reinforce the conclusions.

4. SPI1/PU.1 and clinical data
The observation that SPI1 expression is increased in DNPC is interesting but limited by the small sample size (Fig. 6A). Authors might consider additional datasets available at GEO repository (or others) to strength the analysis. It would also be highly appreciated to analyze SPI1 expression across:
• PCa vs. normal prostate tissue
• Tumors stratified by Gleason score (≤6, 7, ≥8)
• Primary vs. metastatic tumors
• DNPC vs. NEPC

Minor Comments
6. Since this 3D co-culture system is standardized by the authors, including basic characterization metrics (e.g., organoid diameter over time, passaging capacity) would aid reproducibility and future comparisons.

7. Would the current terminology “assembloids” better describe the 3D co-culture system established?

8. Pixel intensity analysis of cytoplasmic vs. nuclear PU.1 staining would better illustrate DB1976’s impact on PU.1 activity.

9. Fig. 3J: Adjustments to show stromal (BioTracker555–) and tumor (BioTracker555+) cells, along with the viability/apoptosis readout, would improve clarity.Fig. 5F: Please use “LAPC9 organoids” (or equivalent) rather than “LAPC9 cells” to avoid implying 2D culture.

10. Fig. 1C: The legend refers to bolded genes, but these are not visible in the figure.

Reviewer #3

(Remarks to the Author)
In the present manuscript Bonollo and co-authors investigate the role of cancer-associated fibroblast (CAFs) in shaping the progression of castration-resistant prostate cancer (CRPC).
Upregulation of ECM-related and ECM-remodeling genes was found in the murine stroma cells of castration-resistant LAPC9-PDX, compared to androgen-sensitive (PNPCa27, BM1829) models. Building on these findings, the author show that LAPC9 tumor cells induce the formation of a pro-fibrotic stromal phenotype, with stromal areas progressively increased over time, and this correlates with the increasing area of tumor-invasive nests. Using novel 3D "hybrid" co-culture conditions the authors confirm the "reactive" pro-tumoral fibrotic phenotype of the LAPC9 CAFs in vitro. At the molecular level, the authors identify transcription factors that might orchestrate the observed phenomena. Among these, the role of transcription factor PU.1, was further investigated. Inhibition of PU.1 impairs organoid growth without impairing CAFs viability.
Overall the findings presented in the present study are novel and of potential great interest for the field.
Technically, they establish a novel in vitro co-culture model that does not rely on standard 2D culturing methods of PDX stroma. This method could potentially preserve CAF properties avoiding the transcriptional changes induced by 2D culturing methods.
Biologically, blockade of PU.1 transcriptional activity could potentially revert pro-tumoral fibrotic phenotype, impacting tumor cell growth and this finding could eventually be translated to clinical studies.
A few minor points need clarification:
1) The authors investigate the pro-fibrotic "reactive" role of castration-resistant CAF in the experiments shown in Figure 3 where they create PNPCa/LAPC9 hybrid co-cultures. The authors show that LAPC9-derived CAFs favour PNPCa cancer organoids growth. Can the author elaborate on the reasons for using PNPCa and not BM18? BM18 are derived from bone metastasis (like the LAPC9, and differently from the PNPCa that are derived from soft tissue) but androgen-sensitive. Do the authors expect the same results when LAPC9-derived CAFs interact with BM18? Are there molecular differences between PNPCa and BM18 that might affect the results?
2) The effect on PU.1 inhibition and tumor growth was investigated. The authors show that PU.1 expressing cells are CD45+ (myeloid or fibroblast) and ~15% of PU.1 + cells are FSP1-positive cells (aka fibroblast). Is it possible that part of the effect observed upon PU.1 pharmacological inhibition is caused by the myeloid PU.1+ cells? Have the authors measured any changes on cytokine secretion (i.e. IL-6, TNFa, GM-CSF)?
3) The authors show transcriptional changes in CAFs upon 2D cultures. Have the authors performed RNA seq for the 3D culturing method implemented? This would be useful to show that the 3D culturing settings prevent alterations. This could also be shown simply by qRT-PCR of a subset of genes that are highly altered in CAFs upon 2D cultures, and not in the 3D cultures.
4) In Figure 4C the first five plots are cut and significance stars are difficult to display.

Version 1:

Reviewer comments:

Reviewer #1

(Remarks to the Author)
The authors have addressed most of my concerns.

Reviewer #2

(Remarks to the Author)
The authors carried out a thorough revision of the manuscript and were able to address the putative comments from my previous review. Their responses are clear, and the updates to the text, figures, and analyses have noticeably improved the clarity and overall presentation of the work. Overall, the study now presents a stronger and more transparent analysis of the stromal differences between androgen-dependent and CRPC-associated prostate cancer models. I have just a few minor points that the authors might consider:

1) Sample size (n = 2–3): In several analyses, the number of biological replicates is relatively small. While the authors clearly explain the experimental constraints, it could be helpful to briefly mention this as a limitation in the Discussion to provide context for interpreting the results.

2) Figure 2I–K: I suggest revising the legend to clarify that Pearson correlation was used for the statistical analysis, and that the linear regression line is included only to help visualize the trend between variables.

3) In the updated legend for Figure 6, the statistical significance thresholds seem to vary between panels (for example, panels A and C). Unless there is a specific reason for this, the authors might consider using a consistent significance threshold across analyses and clearly indicating the chosen level (e.g., $p < 0.05$ or a more stringent threshold such as $p < 0.001$) in the figure legend and/or the Results section.

Reviewer #3

(Remarks to the Author)
I am satisfied that the authors have comprehensively and carefully addressed the concerns raised during the review process. I commend the authors for their thorough and constructive engagement, which has substantially improved the clarity, rigor, and overall presentation of the manuscript. In its current form, I believe the study meets the standards of Communications Biology and is suitable for publication.

Open Access This Peer Review File is licensed under a Creative Commons Attribution 4.0 International License, which permits use, sharing, adaptation, distribution and reproduction in any medium or format, as long as you give appropriate credit to the original author(s) and the source, provide a link to the Creative Commons license, and indicate if changes were made.
In cases where reviewers are anonymous, credit should be given to 'Anonymous Referee' and the source.
The images or other third party material in this Peer Review File are included in the article’s Creative Commons license, unless indicated otherwise in a credit line to the material. If material is not included in the article’s Creative Commons license and your intended use is not permitted by statutory regulation or exceeds the permitted use, you will need to obtain permission directly from the copyright holder.
To view a copy of this license, visit https://creativecommons.org/licenses/by/4.0/

Referee expertise:

Referee #1: tumor microenvironment

Referee #2: prostate cancer

Referee #3: prostate cancer

Reviewers' comments:

Reviewer #1 (Remarks to the Author):

This manuscript “PU.1 is a mediator of the reactive stromal response associated with castration-1 resistant prostate cancer” used in vitro collagen-based 3D tumor-CAF organoid co-culture system to study the role of a pro-fibrotic stroma in the castration-resistant (LAPC9) versus the castration-sensitive (PNPCa, BM18) models. The authors identified the transcription factor PU.1 as a mediator of the LAPC9 pro-fibrotic phenotype and showed that pharmacological inhibition of PU.1 reduced tumor organoid growth. In general, the authors used different approaches to address their conclusions. However, the authors may wish to address the following points:

1. Figure 2G-F, it might be important to see if there is a correlation between tumor invasion and stroma populations in PNPCa and BM18 models like LAPC9 model?

We thank the reviewer for the comment. To answer this question, H&E and Ki67 IHC sections from additional PDXs, PNPCa, BM18, were analyzed by means of histopathological assessment, in comparison to previously analyzed LAPC9 tissues. Due to the low stromal amount in PNPCa and BM18 tissue, for most of the tissues analyzed no tumor invasive nest areas could be identified, differently than LAPC9 tumor tissues in which tumor invasive nests could be detected (as described in the manuscript). This strongly suggests that the tumor cells in this model do not possess invasive properties, in line with the low aggressivity of these models and with a slower tumor growth kinetic in vivo[1,2] . A summary of the tumor invasive nest analysis performed for PNPCa, BM18, and LAPC9 tissues has been added as **Sup. Table 1 (Sup. Data File)** in the revised version of the manuscript (in which we also report if the analysis has been conducted using H&E and/or Ki67 IHC staining, see Reviewer 2, comment 2). The main text has also been adapted accordingly (line 213-216).

2. In LAPC9, does the high fibrotic, myofibroblast-rich stroma contribute to castration resistance phenotype? Would targeting those fibroblasts sensitize LAPC9 to castration?

We thank the reviewer for the comment. The extent of the stroma contribution to CRPC phenotype is highly interesting. Our view is that there is contribution during the development or progression of CRPC, however whether targeting the stroma through PU.1 inhibition would be sufficient to resensitize this model to castration, might be challenging to answer, especially in an already developed CRPC setting.

To address potential interplay between fibroblast phenotype and response to androgens, we assessed growth of LAPC9 organoids (androgen (DHT) removal) in combination with *Spi1* fibroblast targeting. We performed the DB1976 treatment experiment with LAPC9 tumor-CAF collagen co-cultures by growing LAPC9 co-cultures either in the standard DHT 1nM concentration, or without DHT (DHT 0nM) and performed treatment with DB1976 (5uM and 10uM doses) for 2-weeks. The LAPC9 CRPC organoids showed responsiveness to DHT, evident by higher number of organoid count and size compared to no DHT condition, but still, they could form also in the absence of DHT. DB1976 treatment significantly reduced the number of organoids in DHT conditions. Instead, no additive effect was observed upon DB1976 treatment and removal of DHT, indicating that the effect of fibroblast targeting is enhanced in presence of androgens, yet the androgen presence has a more impactful role. These results have been added in the main text (line 544-548) and as **Sup. Fig.9** in the revised version of the manuscript.

3. It would be important to include LAPC9 and LAPC9 + LAPC9 CAFs , LAPC9 + PNPCa CAFs organoids in Figure 3D to determine the impact of matched or unmatched CAFs on LAPC9 organoid size.

We thank the reviewer for the suggestion. Our data indicate that LAPC9 organoid size is comparable upon co-culture with matched (LAPC9-derived CAFs) or unmatched CAF (mouse-dermal fibroblasts, MDFs), showing a smaller size only in the LAPC9 monoculture (no fibroblasts) conditions; differently from what observed for PNPCa organoids as described in Figure 3. To better highlight this observation, we included an additional plot in Figure 5 (describing LAPC9 co-cultures) (**Fig.5F** in the revised version of the manuscript), in which we display the average LAPC9 organoid size in monoculture, in co-culture with MDFs, or with matched CAFs (unsorted tumor and stromal cells), showing a statistically higher area size for both co-culture conditions (line 561-566). Both MDFs and LAPC9 CAFs display a tumor-supportive myofibroblast phenotype since upon DB1976 treatment, we observed a reduction in organoid size for both co-culture set-ups (**Fig. 5E, 5G**). Given that this effect was observed using a non-tumorigenic fibroblast line

(MDFs), we expect a similar effect by using PNPCa-derived CAFs, thus we thought to be unnecessary to repeat this experiment with PNPCa CAFs. Overall, these data suggest that, differently from PNPCa tumor cells, LAPC9 CRPC cells are able to influence the phenotype of adjacent fibroblasts (normal and cancerous) to support their own growth. The manuscript text and figure have been updated accordingly.

4. Figure 3 E-I, it would be important to include an additional control showing the expression of CAF associated genes in LAPC9 CAF without coculture with PNPCa cells to know the basal line of those CAF associated genes and to determine if the upregulation of those genes require being the co-culture with PNPCa.

We thank the reviewer for the suggestion. RT-qPCR analysis of PNPCa and LAPC9 CAF grown in collagen without PNPCa cancer cells (controls) have been performed and the figure has been updated (**Figure 3E, 3F, 3G** in the revised version of the manuscript). Expression analysis of the myofibroblast genes *Acta2*, *Col1a1*, and *Col5a1* (mouse/stromal-specific primers) revealed that their expression is similar for LAPC9 CAF in monoculture or in co-culture with PNPCa tumor cells (line 310-318), in line with their tumor supportive properties described in Figure 3. On the other hand, PNPCa CAF show upregulation of these genes upon co-culture with cancer cells compared to their monoculture controls, in which the expression of these genes is neglectable. RT-qPCR for *Tnc* in the monoculture controls has not been performed, and thus the *Tnc* gene expression plot previously in Figure 3 has been moved to **Sup. Fig. 4B**. Overall, these data indicate that the pro-tumorigenic properties of LAPC9 CAF are intrinsic and they are maintained in collagen 3D co-cultures, differently than 2D cultures (as further discussed for Reviewer 3 comment 3).

5. Figure 4C and line 349, the authors mentioned that “This confirmed enrichment of Spi1 itself and of its targets; C-X-C motif 349 chemokine ligand 1 (Cxcl1), Acta2, Col3a1, thrombospondin-1 (Thbs1), C-X-C motif chemokine ligand (Cxcl2), in the LAPC9 versus PNPCa and BM18 stroma”. However, the data in Figure 4C showed that SPi1 and and Col3a1 are not significantly enriched in LAPC9 vs PNPCa. the authors may wish to consider rewriting that paragraph to reflect only significant changes.

Thank you for the comment. We adjusted the text accordingly by stating “This confirmed enrichment of the Spi1- itself and of its target genes; C-X-C motif chemokine ligand 1 (Cxcl1), Acta2, Col3a1, thrombospondin-1 (Thbs1), C-X-C motif chemokine ligand 2 (Cxcl2), in the LAPC9 versus PNPCa and BM18 stroma (even though Acta2 showed enrichment only in LAPC9 versus PNPCa stroma, and Col3a1 only in LAPC9 versus BM18

stroma) (Fig. 4C)." (line 403-407 in the revised version of the manuscript).

6. Sup. Fig. 5C, the authors should consider running a statistical analysis on this data.

We thank the reviewer for the comment. The statistical analysis has been performed (**Sup. Fig.5E** in the new version of the manuscript).

7. Figure 4H-J, it is less clear how the authors determine whether CD45+ cells possess fibroblast properties with these experiments (line 376). While 13.9% of fibroblasts are PU.1+ FSP1+, it is less clear how a subset of PU.1 and CD45 double-positive cells display clear fibroblast properties (line 379-380)? The authors may wish to include staining of CD45 marker to see if that is colocalize with PU.1 and fibroblast marker.

We thank the reviewer for the comment. Histopathological analysis of CD45 and PU.1 double IHC on LAPC9 tissues, performed by certified pathologist, demonstrated that the vast majority of PU.1+ cells is also CD45+ (while not all CD45+ cells are PU.1+), with the presence of PU.1+/CD45- cells being very low and probably due to analysis artefacts. In the previous version of the manuscript this was only shown qualitatively. To better clarify this point, we included in **Sup. Fig.6** (revised version) a pie chart displaying the percentage of CD45+ PU.1-, CD45+ PU.1+, and CD45- PU.1+ cells (Sup. Fig. 6B, line 430-437). To support this statement, Western Blot analysis on the LAPC9 EpCAM+ tumor fraction, the EPCAM- CD45- stromal/CAF fraction, and EPCAM- CD45+ stromal/immune fraction showed detection of a PU.1 band exclusively in the CD45+ fraction (**Sup. Fig. 6D**). Therefore, the subset of FSP1+ PU.1+ cells (displaying fibroblast properties) is also CD45+ given the co-expression of PU.1 and CD45.

8. Line 416-417 PU.1 is not stromal-specific transcription factor. It is expressed by various immune cells. the authors should consider redrafting that.
We adjusted the text accordingly by stating "PU.1 represents an attractive therapeutic target because its expression is associated with the TME (CAF and immune cells)…" (line 509-510) in the new version of the manuscript).

9. It is beneficial to include in vivo data to determine the role of PU.1 in fibroblast activity and tumor growth using PU.1 conditional mutant mice with specific deletion in fibroblasts. however, we understand that generation of those mice might take longer time.
We thank the reviewer for the comment. We understand that an in vivo validation of the role of PU.1 in fibroblasts would further strengthen our findings, however, due to time

constrains related to obtaining a genetically engineered mouse model with conditional PU.1 deletion in fibroblasts, we were unable to perform this type of experiment. Nonetheless this would be of very high interest for follow-up studies outside of the scope of this manuscript.

Reviewer #2 (Remarks to the Author):

This manuscript investigates phenotypic and molecular differences between androgen-dependent (PNPCa, BM18) and CRPC-associated (LAPC9) prostate cancer stroma using pre-clinical models. It is shown that CRPC stroma adopts a more myofibroblastic profile with greater capacity to stimulate tumor epithelial growth, and the authors identify PU.1 as targetable transcription factor driving this protumorigenic activity.

Overall, the manuscript is well written and data are generally convincing and relevant to the field. Addressing the statistical issues, refining the normalization and clinical dataset analyses, and clarifying some experimental interpretations will significantly strengthen the manuscript.

Major Comments

1. Statistical analysis
The statistical analysis needs careful revision. The authors should provide a full description in Materials and Methods, ensure that the graphical representations match the type of comparisons and inferences, and report appropriate statistical tests for all quantitative analyses.

We thank the reviewer for the suggestion. A section entitled **"Statistical analysis and reproducibility"** (line 1066-1086) has been added to Materials and Methods describing the statistical tests performed in general. Detailed information on specific plots/statistical analysis can also be found in figure legends. The number of replicates per experiment is also specified in the figure legends. We also made sure to include bar plots with individual values or boxplots to show the data distribution following the journal guidelines. In **Fig. 6C** for visualization purposes only, we included a bar plot with mean and standard deviation without individual values. Therefore, for clarity, we included the same plots with reported individual value in **Sup. Fig. 11D** (as also stated in the Materials and Methods section, line 1072-1074).

Specifically:

1.1 The use of R^2 from linear regression to infer correlation strength and direction is inappropriate. The description and conclusions should be adjusted to match regression output, or, preferably, a correlation test (Pearson or Spearman) should be performed, reporting both the coefficient (r) and p value.

We thank the reviewer for the comment. We reported in the figure new plots in which the Pearson correlation coefficient (r) and the p-value for the linear regression analysis is displayed (**Fig. 2I, 2J, 2K** in the revised version of the manuscript). The analysis shows a significant positive correlation between the percentage of tumor invasive area and the amount of total intra-tumoral stroma (**2I**), and Collagen+ intra-tumoral stroma (**2J**), while a positive correlation, even though not significant was found for TNC+ intra-tumoral stroma (**2K**). The main text has also been adapted accordingly (line 217-221) and in the figure legend.

1.2 Figure 2B: The data appear to come from two replicates with repeated measurements (tumor area in mm^2) over time, representing paired data. This should be plotted and tested accordingly (e.g., points connected by lines), while also showing the three replicates per time point as stated in the legend. Please clarify whether n = 2 or 3

We thank the reviewer for the comment. The tumor area measurement at different time points (week2, week3, week5) has been performed from Ki67 IHC tumor tissue sections derived from independent mice, whose tumors were collected at the indicated time points. Therefore, the data are not paired. To better highlight this we represented the data as bar plots (with individual measurements, mean, and standard deviation) and performed a statistical analysis on it (**Fig. 2B** revised version).
Regarding the number of replicates per condition (time point), H&E and Ki67 IHC staining (used for tumor area quantification and invasive nest count) have been performed in n=2 for week 2, week3, and n=3 for week5 (this is why in the previous version of the plot in Fig.2B, three replicates were reported for week5). However, IF staining for intra-tumoral stroma quantification has been performed for the same samples (n=2) in week2, and week 3 and for n=2 samples (out of n=3) for week 5, thus leaving an extra-replicate for week 5, which cannot be included in the correlation analysis (Fig. **2I-K**). To simplify, we included in our updated analysis only the samples for which H&E/Ki67 IHC staining (tumor invasive nest analysis and tumor area quantification) and IF staining (intra-tumoral stroma quantification) has been performed, leading to n=2, whole tumor areas assessed, for each reported plot.

1.3 Figures 2D–F: Since the replicates are independent, bar plots (mean ± SEM) would be more appropriate than scatter plots. Again, clarify whether n = 2 or 3.

In line with our response to comment 1.2, we have included bar plots (mean, standard deviation and individual values) instead of scatter plots (**Fig. 2D, 2E, 2F**) with n=2 tumor tissues from whole tumor areas assessed, per condition (as discussed above).

1.4 Figures 3K–L: The normalization to the PNPCa group is not correctly applied. The mean of the three PNPCa replicates should be set to 1, preserving replicate variance, rather than assigning "1" to each replicate individually.

We thank the reviewer for the observation. The plots (**Fig. 3I, Fig. 3J** in the revised manuscript version) have been updated accordingly in order to preserve the replicate variance in the control group.

2. Ki67 and microinvasion. The prediction of microinvasion from Ki67+ cell distribution overlooks proliferating CAFs. A more robust approach would involve a human-specific epithelial marker (without mouse cross-reactivity) or one known to label LAPC9 epithelial cells (e.g., PSA, CK8).

Thank you for this comment. We can confirm that both H&E- and Ki-67–stained tissue sections were available for the assessment of tumor invasive growth. Ki-67 staining was preferred for the manual outlining of invasive tumor nests because it provided improved visualization of tumor versus non-tumor tissue. We agree that Ki-67 is not a tumor- or epithelium-specific marker and that proliferating stromal cells, including cancer-associated fibroblasts, may also express Ki-67. However, in the tissues examined, tumor and stromal compartments were readily distinguishable based on established histomorphological criteria, as confirmed by the pathologist who performed the invasion assessment.

We acknowledge that the definition and labeling of invasive tumor nests were not entirely objective, as this assessment was performed manually by a pathologist. In addition, at the level of the analyzed tissue sections, invasive growth could regionally be difficult to distinguish from tumor separation given by the tissue section plane. Nevertheless, because invasive tumor nests could overall be reliably identified on morphological grounds, the use of an additional epithelial- or tumor-specific marker was not considered necessary for this analysis. To improve clarity and explicitly address these points, we have rephrased the relevant section of the Methods (line 986-989).

3. Hedgehog signaling

The suggestion that Hedgehog signaling activity is more restrained in PNPCa and BM18 compared to LAPC9 (based on Gli1–3 levels in Fig. 4A–B and S5) remains speculative. Since Gli repressors can have opposing effects, stronger evidence is needed. If protein-level data (full-length vs. repressor forms of Gli) cannot be obtained, the RNAseq dataset should be interrogated for Gli target genes (activators and repressors) to reinforce the conclusions.

We thank the reviewer for the comment. Since protein data have not been generated, we interrogated the RNA-seq dataset to assess the expression of Hedgehog pathway (Hh)-related genes, namely *Gli1, Gli2, Gli3* (transcription factors), *Ptch1, Ptch2* (receptors), *Boc, Cdon, Gas1* (co-receptors)*, Smo* (signal transducer), *Hhip, Kif7,* and *Sufu* (pathway regulators) to gain insight into potential Hh signaling pathway activity in PNPCa and BM18. We determined lower expression of *Boc, Cdon, Gli1, Gli2, Hhip, Ptch1*, *Ptch2*, and *Smo* in LAPC9 versus both PNPCa and BM18 (even though for Cdon, and Ptch2 not all comparisons were significantly different) (**Sup. Fig.5C** in the revised version of the manuscript, line 392-396), suggesting reduced Hh signaling pathway activity in the LAPC9 compared to the other two models. Lower expression of *Gli1*, and *Gli2* in LAPC9 versus both PNPCa, and BM18 was also confirmed by RT-qPCR analysis (mouse/stromal-specific primers), while *Smo* showed lower expression in both BM18 and LAPC9 compared to PNPCa (**Sup. Fig. 5D**). A study (*Yang Z, et al. Dis Model Mech. 2017 Jan 1;10(1):39-52*) showed that Hh signaling pathway maintains the smooth muscle cell layer and hampers microinvasion in prostate cancer, in line with the fact that the LAPC9 model is the only one showing micro-invasive tumor nests among the PDX analyzed.

4. SPI1/PU.1 and clinical data

The observation that SPI1 expression is increased in DNPC is interesting but limited by the small sample size (Fig. 6A). Authors might consider additional datasets available at GEO repository (or others) to strength the analysis. It would also be highly appreciated to analyze SPI1 expression across:

- PCa vs. normal prostate tissue
- Tumors stratified by Gleason score (≤6, 7, ≥8)
- Primary vs. metastatic tumors
- DNPC vs. NEPC

We thank the reviewer for the comment. We understand that the DNPC sample size is smaller compared to the other PCa groups, however, we could not find additional bulk RNA-seq datasets (https://www.prostatecanceratlas.org) available which include this

specific subtype. To obviate the problem, we focused on a more in-depth analysis of the integrated publicly available PCCAT PCa scRNA-seq dataset (https://pccat.net), including samples from normal, primary, metastatic hormone-sensitive, and metastatic CRPC (normal: 12 samples; primary PCa: 60 samples; metastatic hormone-sensitive: 2 samples; metastatic CRPC: 43 samples, NEPC: 10 samples). We contacted the authors who kindly provided raw single-cell gene expression values for the *SPI1* gene as well as other genes of interest, namely *PTPRC* (CD45), *S100A4 (*FSP1*)*, *COL1A1*, *ACTA2*, *TNC*, across the whole dataset. By focusing on *SPI1* gene expression in stromal cell/CAFs in primary PCa, CRPC, and NEPC, we found that *SPI1* showed the highest expression specifically in inflammatory CAFs (iCAFs) in CRPC, but not in primary PCa or NEPC, while no major differences in *SPI1* gene expression were found for the other CAF subtypes (**Fig. 6C**, revised manuscript version). We also did not detect *SPI1* gene expression in iCAF for metastatic hormone sensitive PCa (similar to our PNPCa, and BM18 model), probably due to the smaller sample size for this category (plot not reported in the manuscript). Moreover, the iCAF subpopulation in CRPC showed expression of *PTPRC* (CD45) and *S100A4* (FSP1) (**Fig. 6D**), in line with our LAPC9 data. Therefore, overall, these data corroborate our hypothesis that *SPI1* (PU.1) gene expression is specifically expressed by a CAF subpopulation of hematopoietic origin and/or involved in inflammation and interacting with immune cells (i.e. iCAF/fibrocytes) and it is higher in CRPC tumors (represented by the LAPC9 model). The text describing Figure 6 has been adapted accordingly (line 613-618, 626-640).

To answer the last part of the comment, we also further analyzed the bulk RNA-seq datasets (https://www.prostatecanceratlas.org) to assess *SPI1* expression in normal, primary, and metastatic CRPC, determining a slightly, but statistically significant, higher *SPI1* expression in metastatic CRPC (**Sup. Fig.11A**), while not biologically relevant differences were found in tumors stratified by primary and secondary Gleason patterns (**Sup. Fig.11B**). We finally updated the statistical analysis in **Fig. 6A** to show statistically significant higher *SPI1* gene expression in DNPC versus normal, primary, ARPC, and NEPC.

Minor Comments

6. Since this 3D co-culture system is standardized by the authors, including basic characterization metrics (e.g., organoid diameter over time, passaging capacity) would aid reproducibility and future comparisons.

Thank you for the comment. We have quantified the organoid diameters over time for PNPCa, and LAPC9 organoids formed in suspension, and LAPC9 organoids formed in collagen. The data have been added in **Sup. Fig. 8A, 8B** and the text has been modified accordingly (line 506-510). The PNPCa and LAPC9 organoid when cultured in suspension

(ultra-low attachment) reach an average of 200 μm diameter size in 6-7 days, as already shown in the lab (*Karkampouna, S., et al. Nat Commun **12**, 1117 (2021)*). LAPC9 organoid formation in collagen is instead slower reaching an average diameter size of 100 μm in 14-17 days. Regarding passaging capacity, for this study, PDX-derived organoids (in suspension or in collagen) were not passaged in vitro, but derived from PDX-tissue digested cells (freshly or cryopreserved). From previous experiments it has been established that PNPCa and LAPC9 organoids can be maintained in suspension until few passages (~p8) however not as long term cultures (*Karkampouna, S., et al. Nat Commun **12**, 1117 (2021);* Kang, J., et al. *J Exp Clin Cancer Res* **44**, 290 (2025)).

7. Would the current terminology “assembloids” better describe the 3D co-culture system established?

We thank the reviewer for the suggestion. Assembloids indeed refer to organoids consisting of aggregation among cell types. However, since typically their origin is from induced pluripotent stem cells we would like to refrain from using this term. Our system can mimic, at least partially, biologically relevant tumor-stroma cell-cell interactions due to continuous contact of these cell types, from in vivo tumor to in vitro, rather than assembly performed only in vitro. Therefore, we would prefer to retain that terminologies “tumor-CAF 3D co-cultures” or “organoid-CAF 3D co-cultures” used already in the manuscript.

8. Pixel intensity analysis of cytoplasmic vs. nuclear PU.1 staining would better illustrate DB1976’s impact on PU.1 activity.

We thank the reviewer for the suggestion. The analysis of cytoplasmic vs. Nuclear PU.1 staining was performed for both the primary CAFs and mouse-dermal fibroblast PU.1 staining following DB1976 treatment. The plots have been added as **Figure 5B**, and **Sup. Fig.7B** (line 519) in the revised version of the manuscript. The quantification clearly shows a reduction in the percentage of nuclear PU.1 localization upon DB1976 treatment in a dose-dependent manner. A section in Material and Methods has also been added (line 1051-1060).

9. Fig. 3J: Adjustments to show stromal (BioTracker555–) and tumor (BioTracker555+) cells, along with the viability/apoptosis readout, would improve clarity.

Thank you for the suggestion. Flow cytometry dot plots with BioTracker555 on the x-axis (stroma-tumor) and AnnexinV-FITC (cell viability/apoptosis) staining on the y-axis have been added (**Fig. 3H**, revised version).

Fig. 5F: Please use “LAPC9 organoids” (or equivalent) rather than “LAPC9 cells” to avoid implying 2D culture.

The titles have been updated to state “LAPC9 organoids” (**Fig.5E, 5F, 5G, 5H** in the revised version of the manuscript).

10. Fig. 1C: The legend refers to bolded genes, but these are not visible in the figure.

Thank you for noticing. The plot has been corrected accordingly (**Fig. 1C**, revised version).

Reviewer #3 (Remarks to the Author):

In the present manuscript Bonollo and co-authors investigate the role of cancer-associated fibroblast (CAFs) in shaping the progression of castration-resistant prostate cancer (CRPC).
Upregulation of ECM-related and ECM-remodeling genes was found in the murine stroma cells of castration-resistant LAPC9-PDX, compared to androgen-sensitive (PNPCa27, BM1829) models. Building on these findings, the author show that LAPC9 tumor cells induce the formation of a pro-fibrotic stromal phenotype, with stromal areas progressively increased over time, and this correlates with the increasing area of tumor-invasive nests. Using novel 3D “hybrid” co-culture conditions the authors confirm the “reactive” pro-tumoral fibrotic phenotype of the LAPC9 CAFs in vitro. At the molecular level, the authors identify transcription factors that might orchestrate the observed phenomena. Among these, the role of transcription factor PU.1, was further investigated. Inhibition of PU.1 impairs organoid growth without impairing CAFs viability. Overall the findings presented in the present study are novel and of potential great interest for the field.
Technically, they establish a novel in vitro co-culture model that does not rely on standard 2D culturing methods of PDX stroma. This method could potentially preserve CAF properties avoiding the transcriptional changes induced by 2D culturing methods. Biologically, blockade of PU.1 transcriptional activity could potentially revert pro-tumoral fibrotic phenotype, impacting tumor cell growth and this finding could eventually be translated to clinical studies.
A few minor points need clarification:

1) The authors investigate the pro-fibrotic "reactive" role of castration-resistant CAF in the experiments shown in Figure 3 where they create PNPCa/LAPC9 hybrid co-cultures. The authors show that LAPC9-derived CAFs favour PNPCa cancer organoids growth. Can the author elaborate on the reasons for using PNPCa and not BM18? BM18 are derived from bone metastasis (like the LAPC9, and differently from the PNPCa that are derived from soft tissue) but androgen-sensitive. Do the authors expect the same results when LAPC9-derived CAFs interact with BM18? Are there molecular differences between PNPCa and BM18 that might affect the results?

We thank the reviewer for the comment. Even though the BM18 model is derived from an androgen-dependent bone metastasis and PNPCa from an androgen dependent soft tissue metastasis, the PNPCa model was selected because our rationale for the establishment of hybrid co-cultures was to use two different PCa stromal types which were as similar as possible except for the sensitivity/insensitivity to androgens, to specifically highlight features associated with the CRPC stroma. The fact that the PNPCa stroma is phenotypically "closer" to the LAPC9 stroma than the BM18 stroma is with the LAPC9 stroma is evident by the PCA analysis in **Sup. Fig. 2A**, and by transcriptomic-based gene expression analysis in which CAF-related genes like *Col1a1*, *Col1a2*, *Nox4*, *Spp1*, and *Postn*, show higher expression in PNPCa and LAPC9 stroma compared to BM18 stroma (**Fig.1E**), as discussed in the text (line 151-156). Nonetheless, the stroma of the PNPCa model is still transcriptionally closer to the BM18 stroma, indicating that androgen (in)sensitivity plays a stronger impact in defining stromal phenotypes. Therefore, we expect that a hybrid BM18/LAPC9 co-culture experiment would lead to similar results to what we obtained, with LAPC9-derived CAFs supporting tumorigenesis of androgen-dependent BM18 tumor cells. The text has also been adapted to explain the rationale for model selection (line 301-304).

2) The effect on PU.1 inhibition and tumor growth was investigated. The authors show that PU.1 expressing cells are CD45+ (myeloid or fibroblast) and ~15% of PU.1 + cells are FSP1-positive cells (aka fibroblast). Is it possible that part of the effect observed upon PU.1 pharmacological inhibition is caused by the myeloid PU.1+ cells? Have the authors measured any changes on cytokine secretion (i.e. IL-6, TNFa, GM-CSF)?

We thank the reviewer for the comment. To answer this question, we performed RT-qPCR analysis for the fibroblast/myofibroblast marker *Acta2*, and the myeloid/pro-inflammatory genes *Cd45, Cd11b, Csf2, Il1a, Il1b, Il6, Tnf* on LAPC9 tumor-CAF co-cultures after a 2-week treatment with DB1976. Compared to the untreated condition, we observed a statistically significant lower *Acta2* expression upon treatment, while no statistically significant changes (except for *Cd11b*) for the myeloid/pro-inflammatory markers (**Sup. Fig. 9D** in the revised version of the manuscript). Furthermore, the expression of the myeloid/inflammatory markers was overall lower that *Acta2* (with very

high CT values or undetectable for some replicates). Therefore, these data overall suggest that the observed effect upon DB1976 treatment is mostly due to PU.1 activity inhibition in PU.1-positive fibroblast/MFB-like cells rather than myeloid PU.1-positive cells. These results have been added in the main text (line 524-534).

3) The authors show transcriptional changes in CAFs upon 2D cultures. Have the authors performed RNA seq for the 3D culturing method implemented? This would be useful to show that the 3D culturing settings prevent alterations. This could also be shown simply by qRT-PCR of a subset of genes that are highly altered in CAFs upon 2D cultures, and not in the 3D cultures.

We thank the reviewer for the comment. Unfortunately, RNA-seq on the generated collagen 3D co-cultures has not been performed. However, we performed RT-qPCR (mouse/stromal-specific primers) for selected CAF genes (*Acta2*, *Col1a1*, *Col2a1*, *Col5a1*, *Mmp3*, *Mmp9*, known to be upregulated in LAPC9 vs PNPCa stroma*)* on RNA extracted from PNPCa, and LAPC9 co-cultures formed in suspension and transferred into collagen, and compared their expression with the PNPCa and LAPC9 stroma after tumor-tissue digestion (p0), and PNPCa/LAPC9-derived fibroblasts cultured in 2D. We determined higher expression for stromal *Acta2*, *Col1a1*, *Col2a1*, *Col5a1*, and *Mmp9* in LAPC9 vs PNPCa CAFs in both collagen 3D co-cultures and p0 stroma (**Fig. 3B** in the revised version of the manuscript, line 288-296), but not in the p2/2D fibroblasts, in which the overall expression of these genes was downregulated (**Sup. Fig. 2C** in the revised version of the manuscript, line 288-296). The only gene showing upregulation in p2/2D was *Acta2*, in line with the fact that adhesion to plastic is known to induce α-SMA upregulation, altering fibroblast phenotype (line 288-296). These data, together with the RT-qPCR data on the PNPCa/LAPC9 hybrid co-cultures, in which we showed that LAPC9 CAF grown in 3D retained higher CAF marker gene expression (*Acta2, Col1a1*, and *Col5a1*) than PNPCa CAF regardless of the presence of PNPCa tumor cells (**Fig. 3E-G,** discussed in Reviewer 1 comment 4) strongly suggest that in 3D co-cultures, differently than in 2D, we can retain, at least partially, features of the original PDX stroma/CAF. This is also further supported by the fact that LAPC9-derived CAF possess tumor-supporting properties, as demonstrated by the data on the hybrid PNPCa/LAPC9 co-culture system (**Fig. 3D, 3H-J**) and by the LAPC9 tumor-CAF co-cultures treated with DB1976 (described in **Fig.5**).

4) In Figure 4C the first five plots are cut and significance stars are difficult to display

Thank you for noticing. The plots in **Figure 4C** have been updated accordingly.

REVIEWERS' COMMENTS:

Reviewer #1 (Remarks to the Author):

The authors have addressed most of my concerns.

We thank the reviewer for the acknowledgement of our work and positive response.

Reviewer #2 (Remarks to the Author):

The authors carried out a thorough revision of the manuscript and were able to address the putative comments from my previous review. Their responses are clear, and the updates to the text, figures, and analyses have noticeably improved the clarity and overall presentation of the work. Overall, the study now presents a stronger and more transparent analysis of the stromal differences between androgen-dependent and CRPC-associated prostate cancer models. I have just a few minor points that the authors might consider:

1) Sample size ($n = 2–3$): In several analyses, the number of biological replicates is relatively small. While the authors clearly explain the experimental constraints, it could be helpful to briefly mention this as a limitation in the Discussion to provide context for interpreting the results.

We thank the reviewer for the comment. To this end, we mentioned the relatively small number of biological replicates as a potential limitation of the study in the Discussion section (line 586-588 of the revised manuscript version).

2) Figure 2I–K: I suggest revising the legend to clarify that Pearson correlation was used for the statistical analysis, and that the linear regression line is included only to help visualize the trend between variables.

We thank the reviewer for the comment. The figure legend has been adapted accordingly (line 1173-1175).

3) In the updated legend for Figure 6, the statistical significance thresholds seem to vary between panels (for example, panels A and C). Unless there is a specific reason for this, the authors might consider using a consistent significance threshold across analyses and clearly indicating the chosen level (e.g., $p < 0.05$ or a more stringent threshold such as $p < 0.001$) in the figure legend and/or the Results section.

We thank the reviewer for the comment. For visualization purposes, we chose to show only statistically significant differences with p-value < 0.001 (denoted with ***) in panel

6A, while for panel 6C we showed differences from p< 0.05 (*). However, for consistency with all the other plots in the manuscript, we decided to include in panel 6A (revised version) also the differences with p-value < 0.05 (*) and p <0.01 (**). The figure legend (line 1271) and plot (Fig. 6A) have been adapted accordingly. Similarly, we also updated plots and legends for Suppl. Fig. 11B and 11C.

We also thank the reviewer for this comment, as we noticed a mistake in our figure legends reporting p-values. Previously, we reported "**" as indicative of p<0.005, while this indicates p<0.01. In the revised version of the manuscript, we now report correctly the threshold symbols based on the p-values: p< 0.05 (*), p< 0.01 (**), p< 0.001 (***), p< 0.0001 (****).

Reviewer #3 (Remarks to the Author):

I am satisfied that the authors have comprehensively and carefully addressed the concerns raised during the review process. I commend the authors for their thorough and constructive engagement, which has substantially improved the clarity, rigor, and overall presentation of the manuscript. In its current form, I believe the study meets the standards of Communications Biology and is suitable for publication.

We thank the reviewer for the constructive comments and acknowledgement of the improved version of the manuscript.
