# Supplementary material for: PU.1 is a mediator of the reactive stromal response associated with castration-resistant prostate cancer
Source: Commun Biol. 2026 May 29;9:1174. doi: 10.1038/s42003-026-10297-7 (PMC13547233; doi:10.1038/s42003-026-10297-7)
Supplement: Supplementary file 2 [file 42003_2026_10297_MOESM2_ESM.pdf]

Supplementary Information

Supplementary Figure 1: LAPC9 tumor cell invasiveness increases over the time of tumor growth while their proliferation capacity is unaltered.

Supplementary Figure 2: PDX stromal cells show distinct gene expression profiles following tissue digestion and in 3D collagen-based co-cultures, but not upon *in vitro* 2D culturing.

Supplementary Figure 3: PDX-derived stromal cells are retained in ULA conditions and acquire a typical fibroblast phenotype upon collagen co-culture.

Supplementary Figure 4: LAPC9 CAFs display myCAF properties and support the viability of PNPCa tumor cells in collagen-based hybrid PNPCa tumor + LAPC9 CAF co-cultures.

Supplementary Figure 5: *Spi1* shows higher activity in LAPC9 stroma, while Hedgehog pathway genes show higher activity in PNPCa and BM18 stroma.

Supplementary Figure 6: PU.1 in LAPC9 is expressed by CD45+ immune/hematopoietic-derived cells and it is associated with reactive stromal phenotype.

Supplementary Figure 7: PU.1 is a mediator of the pro-fibrotic phenotype in mouse dermal fibroblasts.

Supplementary Figure 8: Blockade of PU.1 activity inhibits the myofibroblast phenotype, impacting LAPC9 organoid formation in LAPC9 tumor-CAF collagen co-cultures.

Supplementary Figure 9. The effect of pharmacological inhibition of PU.1 activity is not altered in presence of androgens, and is mediated by fibroblast/MFB-like cells.

Supplementary Figure 10: Blockade of PU.1 activity inhibits the myofibroblast phenotype but does not directly impact tumor organoid and fibroblast viability.

Supplementary Fig.11. PU.1 (*SPI1*)-mediated formation of a pro-fibrotic stroma is associated with CRPC progression.

Supplementary Table 1: Tumor invasive nest analysis

Supplementary Table 2: *Spi1*-target genes

Supplementary Table 3: Antibodies used

Supplementary Table 4: Mouse-specific gene primers

Uncropped Western Blot images from Sup. Fig. 6D with size markers

Supplementary Figure 1

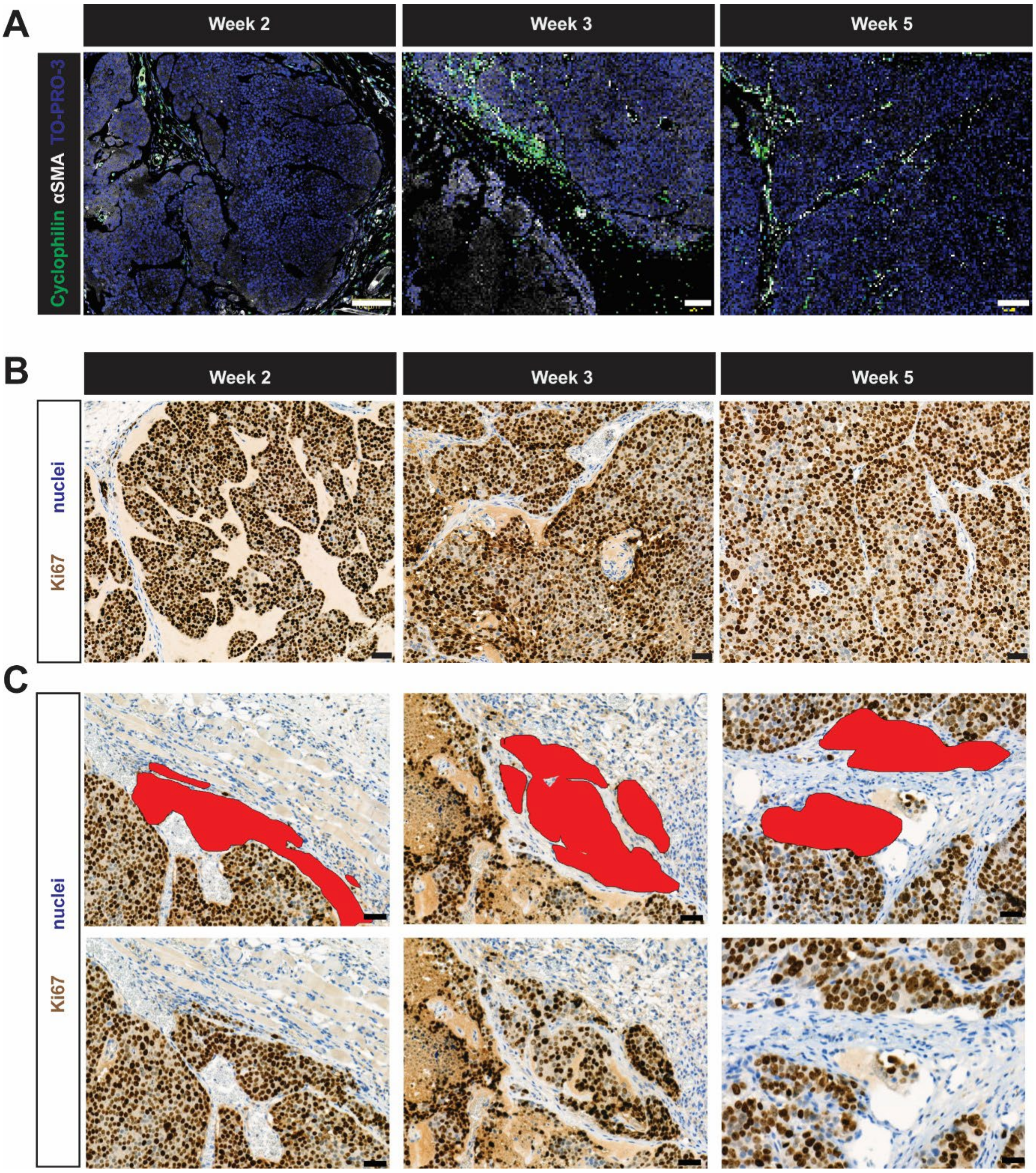

Supplementary Figure 1. LAPC9 tumor cell invasiveness increases over the time of tumor growth while their proliferation capacity is unaltered. A. Representative immunofluorescence staining images of the collected FFPE LAPC9 tumor tissues at week 2, week 3, and week 5. Cyclophilin A (green), and α-SMA (blue channel, represented in white). TOPRO-3 (far red channel, represented in blue) marks the nuclei. Scale bar: 100 µm. **B.** Representative immunohistochemistry staining images for Ki67. Scale bar: 50 µm. **C.** Representative images of pathologist-annotated tumor invasive nests in the LAPC9 tissue. Matched tumor invasive nests are shown, with (top) and without (bottom) the annotation label (red). Scale bar: 50 µm**.**

Supplementary Figure 2

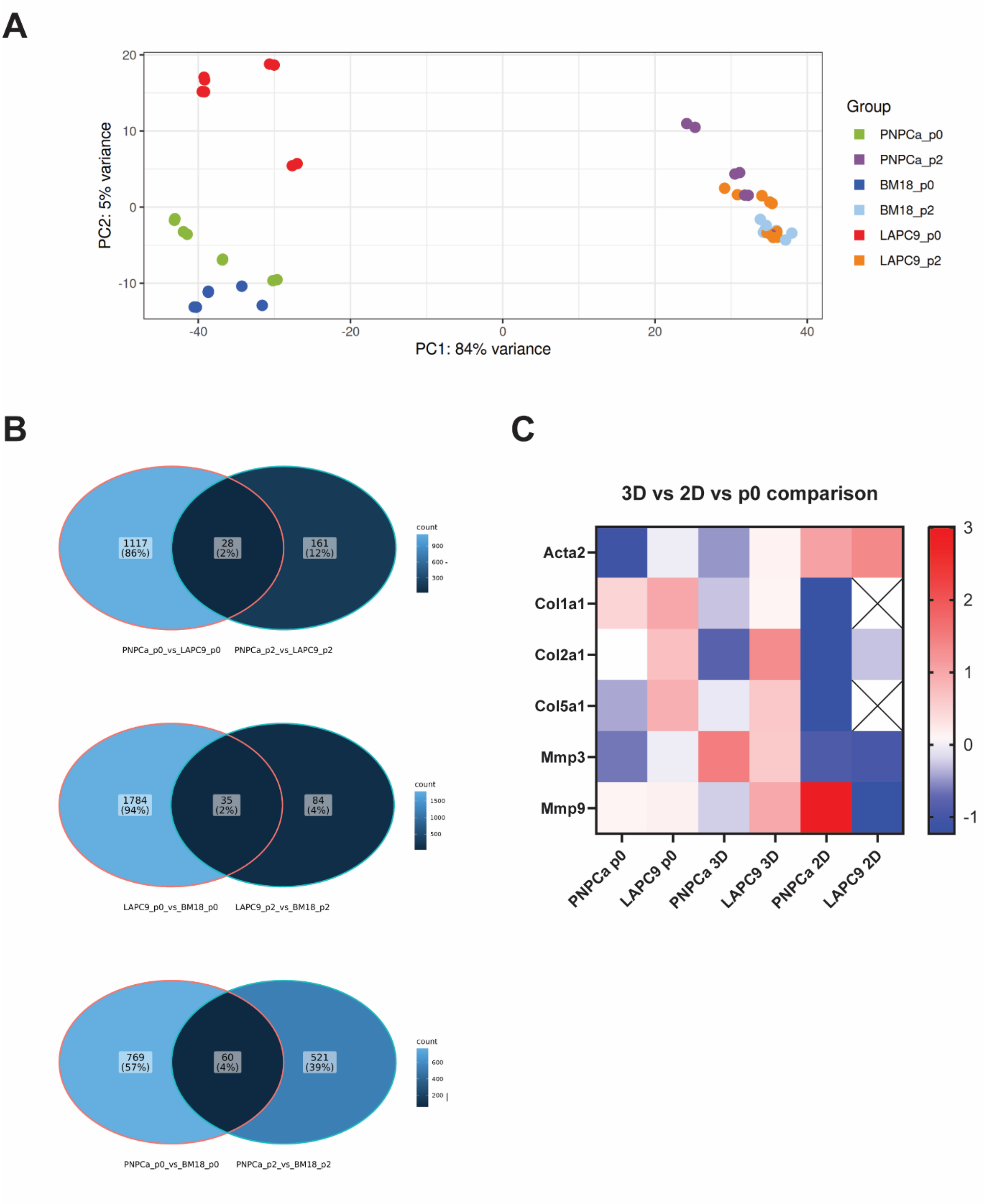

Supplementary Figure 2. PDX stromal cells show distinct gene expression profiles following tissue digestion and in 3D collagen-based co-cultures, but not upon *in vitro* 2D culturing. A. Principal components analysis (PCA) plot of the gene expression profile of PNPCa, BM18, and LAPC9 stromal (mouse) digested single cells (p0) and early-passage PDX-derived fibroblasts (p2). Each dot represents a technical/biological replicate. **B**. Venn Diagrams showing the overlap of DEGs between the PNPCa vs LAPC9, LAPC9 vs BM18, and PNPCa vs BM18 comparisons in the p0 and p2 conditions. **A, B.** n=5 biological replicates for LAPC9 p2, n=4 biological replicates for PNPCa p0, PNPCa p2, and LAPC9 p0, n=3 biological replicates for BM18 p0, and BM18 p2; n=2 technical replicates for each biological replicate. **C.** Heatmap representing the RNA expression of *Acta2*, *Col1a1*, *Col2a1*, *Col5a1*, *Mmp3*, and *Mmp9* in PNPCa and LAPC9 stromal cells after PDX tissue digestion (p0), in collagen tumor-CAF 3D co-cultures (3D), and in early-passage (p2) PDX-derived fibroblasts (2D). The color code reports the mean of the logarithm of the relative expression ($\log(2^{-\Delta Ct})$) of the indicated gene to *Actb* (housekeeping gene), with blue indicating lower expression, and red higher expression. Row-wise Z-score normalization per each gene across samples was performed for the heatmap visualization, n=2 biological replicates with each n=3 technical replicates per condition. Missing values are crossed in the heatmap.

Supplementary Figure 3

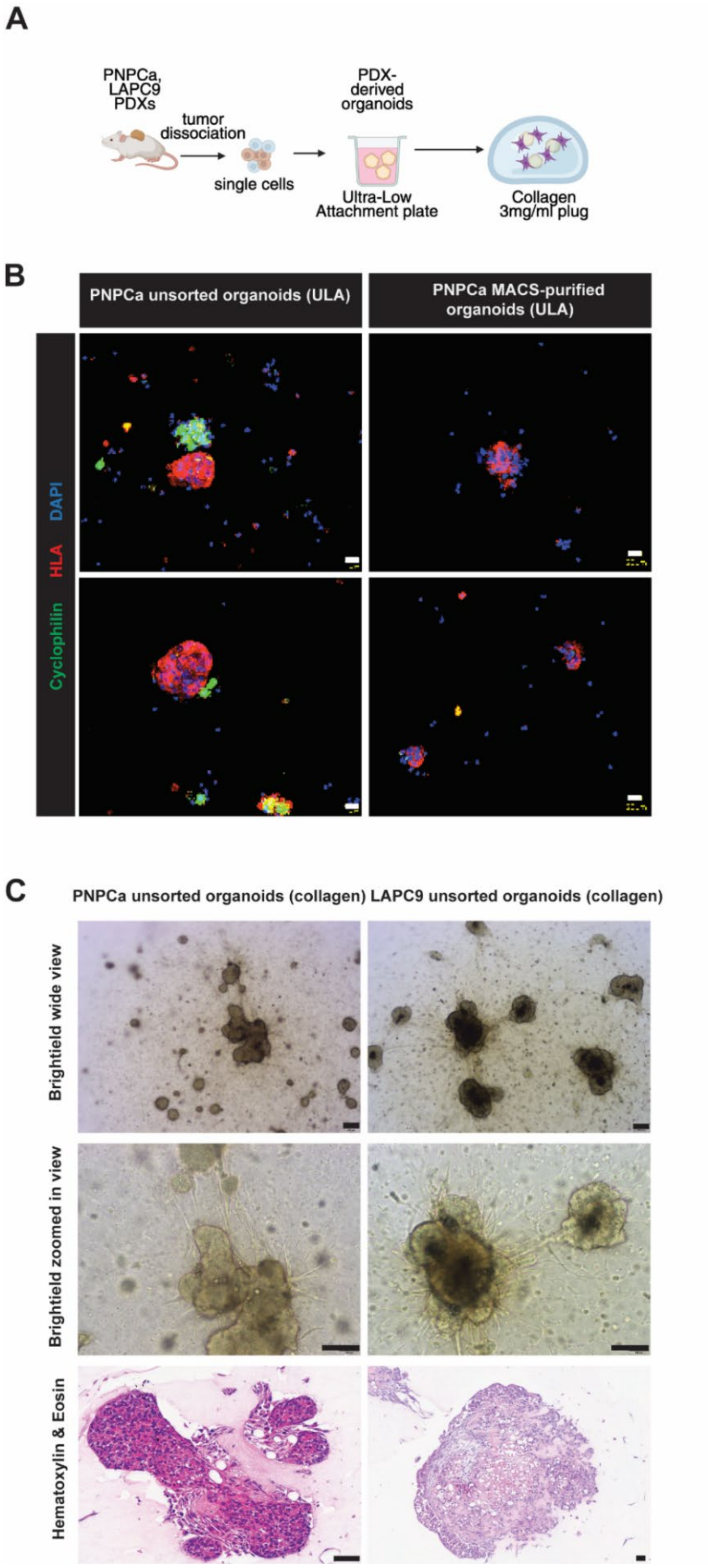

Supplementary Figure 3. PDX-derived stromal cells are retained in ULA conditions and acquire a typical fibroblast phenotype upon collagen co-culture. A. Schematic overview of the newly established PDX-derived collagen-based co-culture system. PNPCa and LAPC9 tissues were digested into single cells. Unsorted (tumor + stroma) single cells were seeded in ULA conditions to allow organoid formation and subsequently transferred into collagen 3mg/ml drops. Created in BioRender. Karkampouna, S. (2026) https://BioRender.com/0g4mdkx. **B.** Representative whole-mount immunofluorescence staining performed on PNPCa organoids obtained from unsorted (tumor + stroma) or tumor cell-only (MACS-depleted), grown in ULA condition. Cyclophilin A (green), HLA (red). DAPI (blue) marks the nuclei. Scale bar: 20 µm. **C.** Representative brightfield wide view (top) and zoomed in view (middle) images, and H&E (bottom, FFPE sections) images of PNPCa and LAPC9 tumor-CAF co-cultures without prior purification sorting. Scale bar brightfield wide view: 200µm. Scale bar brightfield zoomed-in view: 100µm. Scale bar HE: 50µm.

Supplementary Figure 4

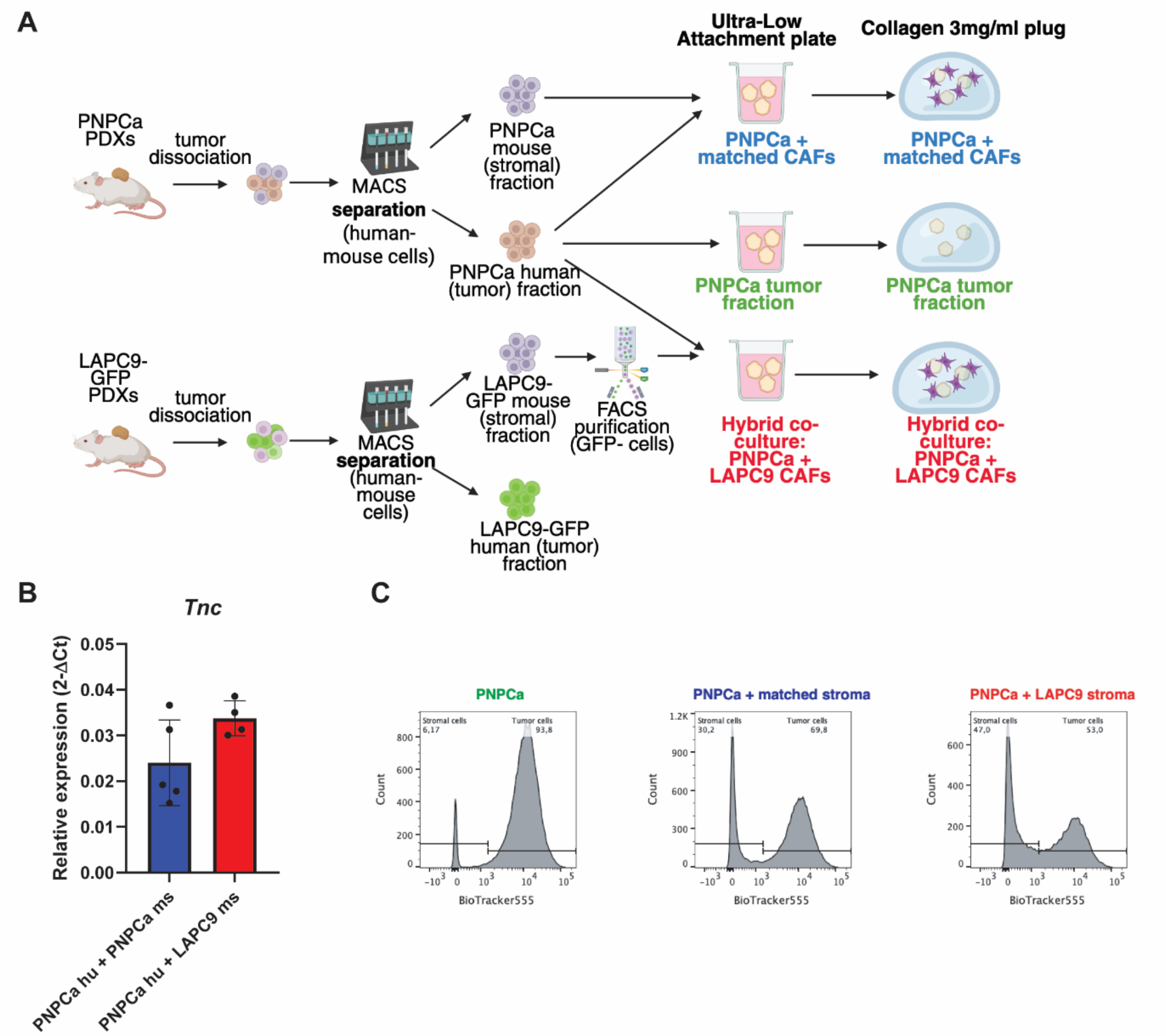

D

Annexin V +/- cells	Exp#1		Exp#2		Exp#3	
	%Tumor cells/ Live cells	%Tumor cells/ Apoptotic cells	%Tumor cells/ Live cells	%Tumor cells/ Apoptotic cells	%Tumor cells/ Live cells	%Tumor cells/ Apoptotic cells
PNPCa tumor cells	19.10	80.90	39.1	60.9	2.5	97.5
PNPCa tumor + PNPCa stromal cells	34.00	66.00	54.1	45.9	9	91
PNPCa tumor + LAPC9 stromal cells	41.50	58.50	67.3	32.7	10	90

Supplementary Figure 4. LAPC9 CAFs display myCAF properties and support the viability of PNPCa tumor cells in collagen-based hybrid PNPCa tumor + LAPC9 CAF co-cultures. A. Schematic of the establishment of hybrid PNPCa tumor + LAPC9 CAF co-cultures. PNPCa and LAPC9 tissues were digested into single cells, and the tumor (human) and stromal (mouse) fractions were separated via MACS. PNPCa tumor cells were recombined in ULA conditions with PNPCa-matched or LAPC9-GFP-derived CAFs (MACS and FACS-purified) or grown without CAFs as control. Before recombination PNPCa tumor cells were labelled with the BioTracker555 lipophilic dye. After organoids had formed, they were transferred into collagen drops**.** Created in BioRender. Karkampouna, S. (2026) https://BioRender.com/36utls4. **B.** RT-qPCR performed on RNA extracted from PNPCa tumor + PNPCa CAFs, and PNPCa tumor + LAPC9 CAFs collagen co-cultures, using mouse-specific primers. Mean, standard deviation, and each single value (technical and biological replicates) of the logarithm of the relative expression ($\log(2^{-\Delta Ct})$) of *Tnc* relative to *Actb* and *Hprt* (housekeeping genes) is reported, n=2 biological replicates with each n=3 technical replicates. Unpaired t-test, $p < 0.05$ (*), $p < 0.01$ (**), $p < 0.001$ (***), $p < 0.0001$ (****). **C.** Representative flow cytometry analysis of PNPCa + LAPC9 stroma (CAFs), PNPCa + matched stroma (CAFs) or PNPCa monocultures, performed at co-culture Day4. BioTracker555 signal was used to gate tumor (BioTracker555-positive) and stromal (BioTracker555-negative) cells. **D.** Table reporting the percentage of tumor/live, and tumor/apoptotic cells per co-culture condition (PNPCa tumor, PNPCa tumor + PNPCa CAFs, and PNPCa tumor + LAPC9 CAFs) over n=3 independent flow cytometry experiments (biological replicates).

Supplementary Figure 5

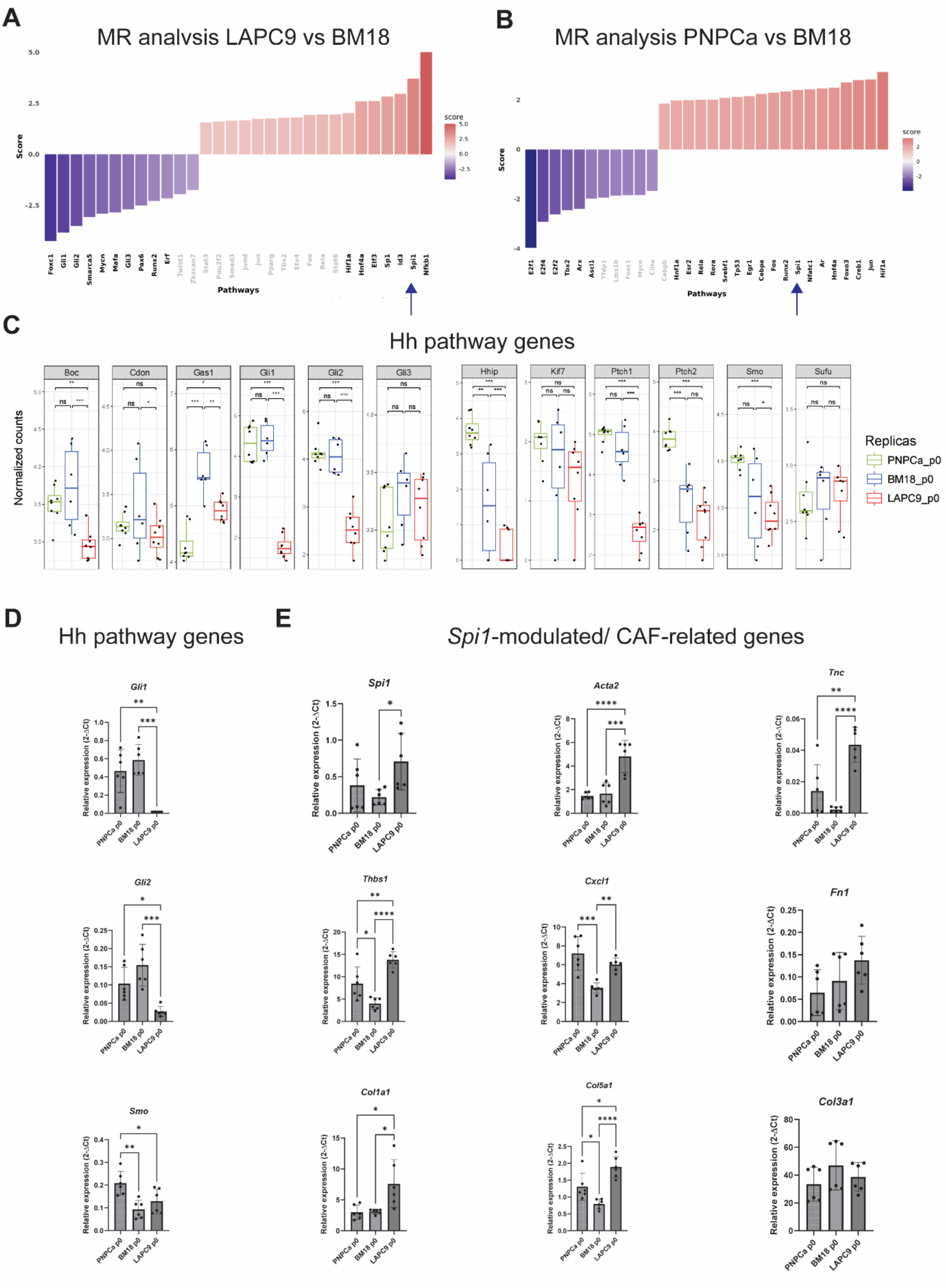

Supplementary Figure 5. *Spi1* shows higher activity in LAPC9 stroma, while Hedgehog pathway genes show higher activity in PNPCa and BM18 stroma. **A.** Bar plot representing the MR (transcription factors) showing higher/lower activity (score) in LAPC9 vs BM18 stroma from another dataset of PDX tumors [1] (with multiple biological replicates). *Spi1* position is indicated with an arrow. Genes highlighted in bold show a statistically significant higher/lower activity (p-value < 0.05). **B.** Bar plot representing MR (transcription factors) showing higher/lower activity in PNPCa vs BM18 stroma (BRB-seq dataset). Genes highlighted in bold show a statistically significant higher/lower activity (p-value < 0.05). **C.** Expression levels (normalized counts DEseq2) of Hedgehog (Hh) pathway-related genes in p0 PNPCa, BM18, and LAPC9 stromal cells. n=4 biological replicates for PNPCa and LAPC9 (n=2 technical replicates each), n=3 biological replicates for BM18 (n=2 technical replicates each). Statistics: Wald test with Benjamini and Hochberg multiple correction, p< 0.05 (*), p< 0.01 (**), p< 0.001 (***). **D, E.** RT-qPCR performed on RNA extracted from PNPCa, BM18, and LAPC9 stromal cells using mouse-specific primers for Hh pathway genes (**D**) and *Spi1*-modulated /known CAF-related genes (**E**). Mean, standard deviation, and each single value (technical and biological replicates) of the logarithm of the relative expression ($\log(2^{-\Delta Ct})$) of the indicated gene to *Actb* and *Hprt* (housekeeping genes) is reported, n=2 biological replicates with each n=3 technical replicates per condition. Ordinary one-way ANOVA with Tukey's multiple comparison, p< 0.05 (*), p< 0.01 (**), p< 0.001 (***), p< 0.0001 (****).

Supplementary Figure 6

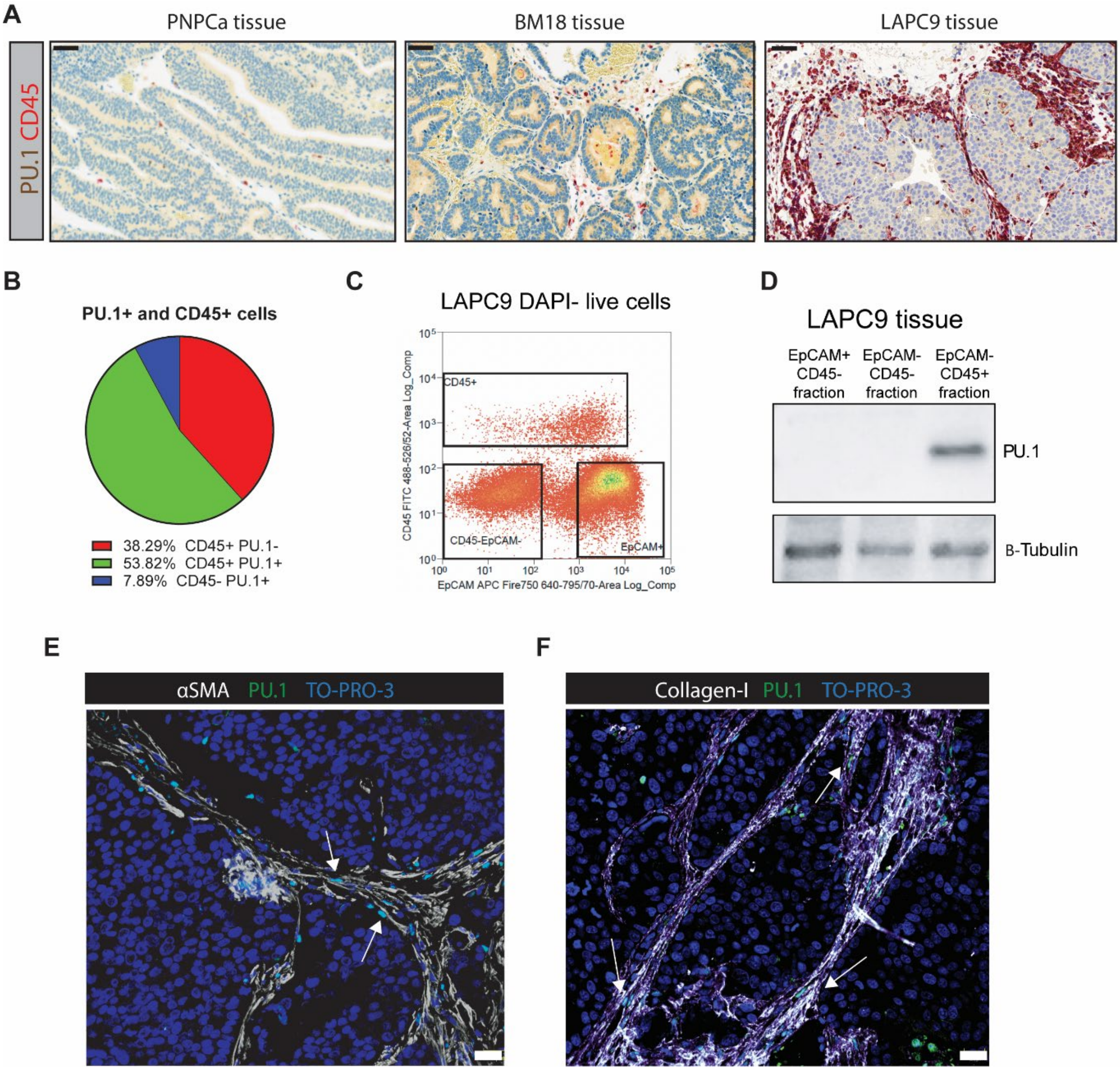

Supplementary Figure 6. PU.1 in LAPC9 is expressed by CD45+ immune/hematopoietic-derived cells and it is associated with reactive stromal phenotype. **A.** Double-immunohistochemistry (IHC) staining for PU.1 (brown) and CD45 (red) performed on PNPCa, BM18, and LAPC9 tissue sections. Scale bar: 50 µm (full image and magnification). **B.** PU.1-CD45 double-IHC staining quantification analysis. Pie chart reporting the percentage of CD45+ PU.1- (red), CD45+ PU.1+ (green), and CD45- PU.1+ (blue) cells over the total of PU.1+ and CD45+ cells in LAPC9 tissue. The percentage is the average of n=2 biological replicates. **C.** FACS gating to isolate LAPC9 EpCAM+ CD45- (tumor epithelial), EpCAM- CD45+ (immune), and EpCAM- CD45- (stromal) fractions. DAPI-positive dead cells were excluded before sorting. Representative dotplot of n=2 independent FACS experiment performed. **D.** Western Blot performed on LAPC9 EpCAM+ CD45- (tumor epithelial), EpCAM- CD45+ (immune), and EpCAM- CD45- fractions for the visualization of PU.1 on n=2 independent LAPC9 biological replicates (only one representative example shown). B-tubulin was used as the loading control. **E, F.** Immunofluorescence staining of LAPC9 tissue sections. **E**: α-SMA (blue channel, represented in white), PU.1 (green). **F**: Collagen type-I (blue channel, represented in white), PU.1 (green). TOPRO-3 (far red channel, represented in blue) marks the nuclei. Arrows indicate examples of PU.1-positive cells in α-SMA and collagen-I positive stromal areas. Representative image of n=2 biological replicates. Scale bar: 100 µm.

Supplementary Figure 7

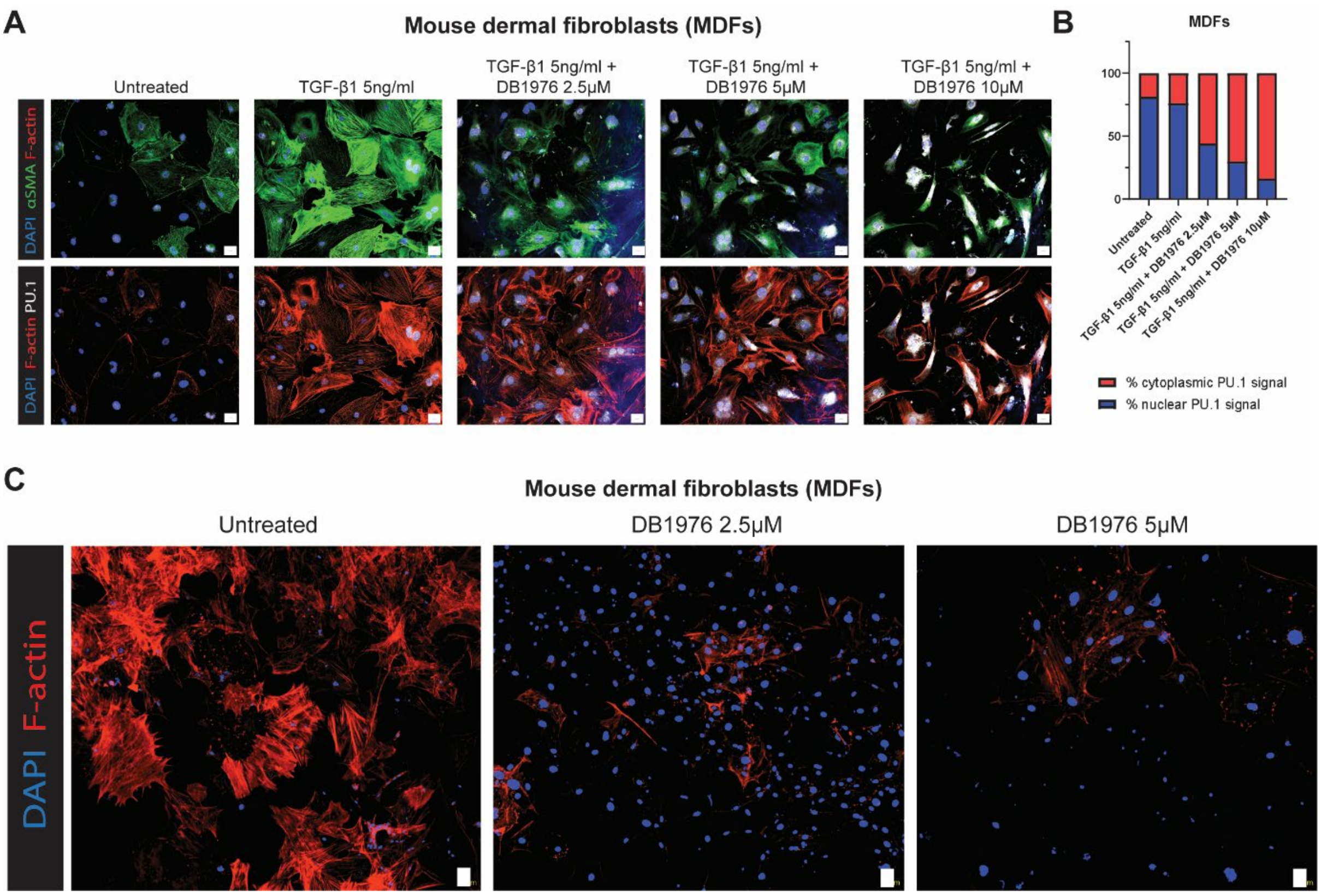

Supplementary Figure 7. PU.1 is a mediator of the pro-fibrotic phenotype in mouse dermal fibroblasts. A. Whole-mount staining images of formalin-fixed MDFs after TGF-β1 5ng/ml and DB1976 (indicated dose) co-treatment for 5 days in serum-starved conditions. Top panels: α-SMA (green), PU.1 (far red channel, represented in white), bottom panels: F-actin (red), PU.1 (white). DAPI (blue) marks the nuclei. Scale bar: 20 µm. **B.** PU.1 nuclear (blue bars) and cytoplasmic (red bars) signal quantification analysis performed with the Fiji software based on the MDF immunofluorescence images described in *A (bottom row)*. PU.1 nuclear signal was defined by co-localization with DAPI signal (marker for cell nuclei). Average of n=2 technical replicates reported. **C.** Whole-mount staining images of MDFs grown in collagen 3mg/ml plugs after 2-week DB1976 treatment (indicated dose). Representative image of n=3 technical replicates. F-actin (red). DAPI (blue) marks the nuclei. Scale bar: 20 µm.

Supplementary Figure 8

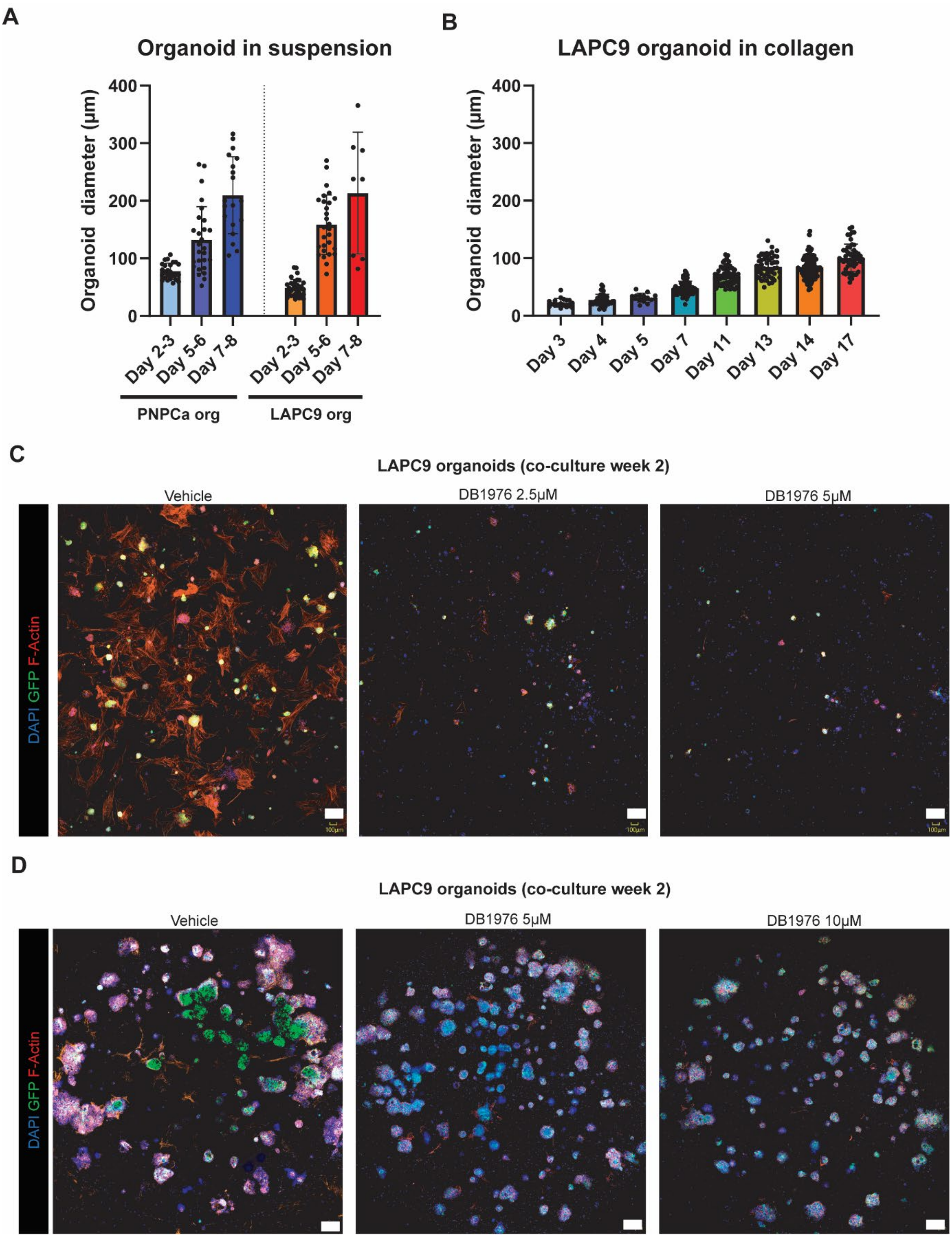

Supplementary Figure 8. Blockade of PU.1 activity inhibits the myofibroblast phenotype, impacting LAPC9 organoid formation in LAPC9 tumor-CAF collagen co-cultures. A. Bar plots representing the diameter (µm) of PNPCa and LAPC9 organoids formed in suspension (ULA setting) at Day 2-3, Day 5-6, and Day 7-8. Measurements have been performed with Fiji by using brightfield microscope images. Mean, standard deviation, and each single measurement (dots) reported. **B.** Bar plots representing the diameter (µm) of LAPC9 organoids formed in collagen at the indicated days. Measurements have been performed with Fiji by using brightfield microscope images. Mean, standard deviation, and each single measurement (dots) reported. **C.** Whole-mount fluorescence staining images of formalin-fixed LAPC9-GFP tumor-CAF collagen co-cultures (unsorted tumor and stromal cells) stained with Phalloidin (F-actin) after 2-week DB1976 2.5µM and 5µM treatment. Representative image of n=3 technical replicates. GFP (green), F-actin (red). DAPI (blue) marks the nuclei. Scale bar: 200 µm. **D.** Whole-mount fluorescence staining images of formalin-fixed LAPC9-GFP tumor-CAF collagen co-cultures (unsorted tumor and stromal cells) pre-formed in ULA. Treatment with DB1976 5µM and 10µM was performed for 1 week after co-culture transferring into collagen. Representative image of n=3 technical replicates. GFP (green), F-actin (red). DAPI (blue) marks the nuclei. Scale bar: 200 µm.

Supplementary Figure 9

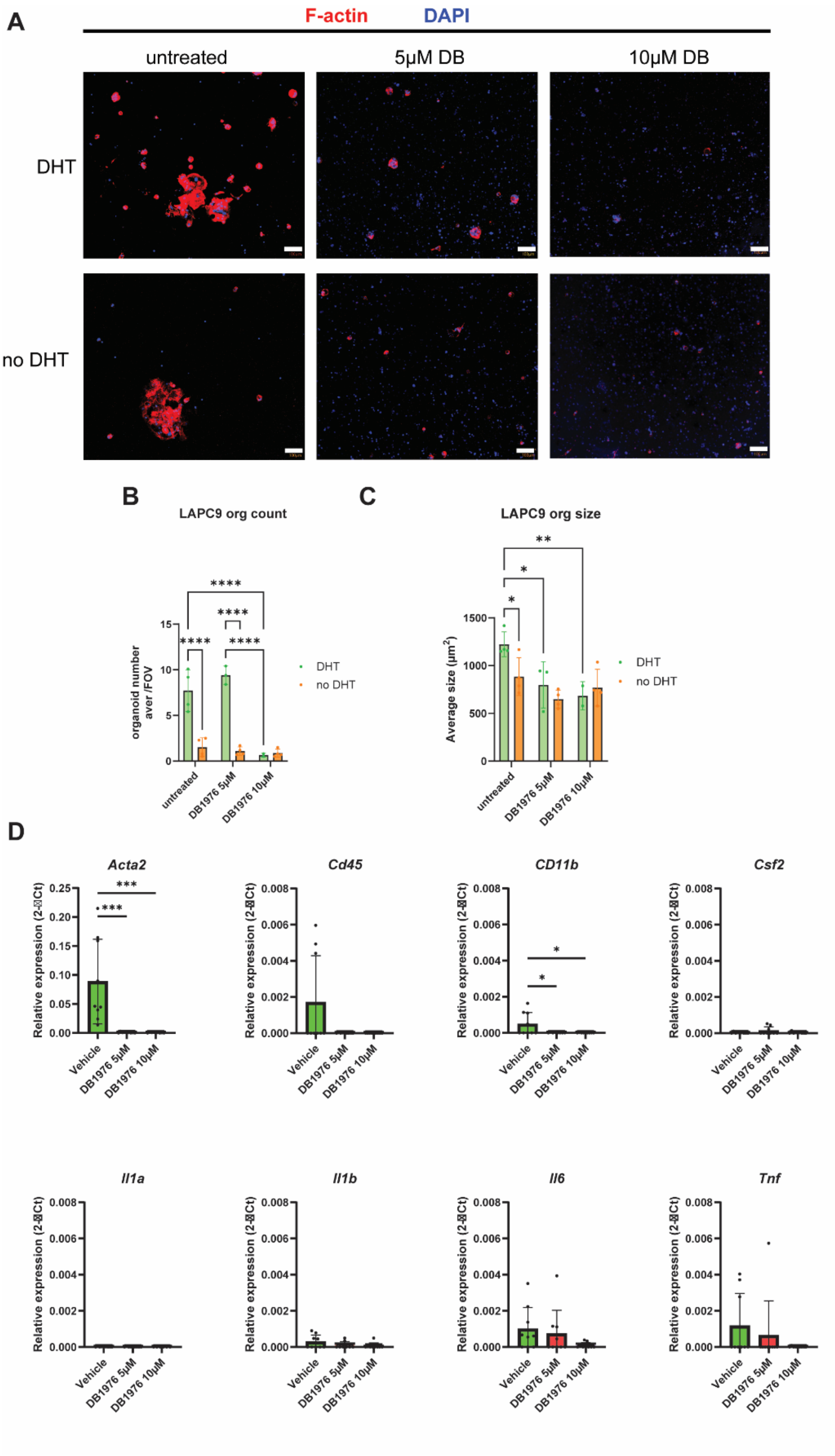

Supplementary Figure 9. The effect of pharmacological inhibition of PU.1 activity is not altered by the presence of androgens, and it is mediated by fibroblast/MFB-like cells. A. Whole-mount fluorescence staining images of formalin-fixed LAPC9-GFP tumor-CAF collagen co-cultures (unsorted tumor and stromal cells) stained with Phalloidin (F-actin) after 2-week DB1976 5µM and 10µM treatment in the presence (DHT 1nM) or absence of DHT (DHT 0nM). Representative image of n=4, whole well, technical replicates, F-actin (red). DAPI (blue) marks the nuclei. Scale bar: 100 µm. **B,C.** Fiji quantification of (**B**) the average organoid number per field of view (FOV), and (**C**) average organoid area size (µm^2) based on F-actin (phalloidin) staining images of LAPC9 organoids described in **A**. Mean, standard deviation, and individual measurements (dots) reported. Statistics: Two-way ANOVA with Tukey multiple comparisons test, $p< 0.05$ (*), $p< 0.01$ (**), $p< 0.001$ (***), $p< 0.0001$ (****). FOV images 4-9 per condition **D.** RT-qPCR performed on LAPC9 3D co-cultures after 2-weeks of DB1976 treatment at the indicated doses using mouse-specific primers against fibroblast and myeloid cell type specific markers. Mean, standard deviation, and each single value (technical and biological replicates) of the logarithm of the relative expression ($\log(2^{-\Delta Ct})$) of the indicated gene to *Actb* (housekeeping gene) is reported, n=3 biological replicates with each n=3 technical replicates per condition. Ordinary one-way ANOVA with Tukey's multiple comparison, $p< 0.05$ (*), $p< 0.01$ (**), $p< 0.001$ (***), $p< 0.0001$ (****). Y-axis was set to 0.25 for *Acta2* (higher expression), and to 0.008 for all other genes.

Supplementary Figure 10

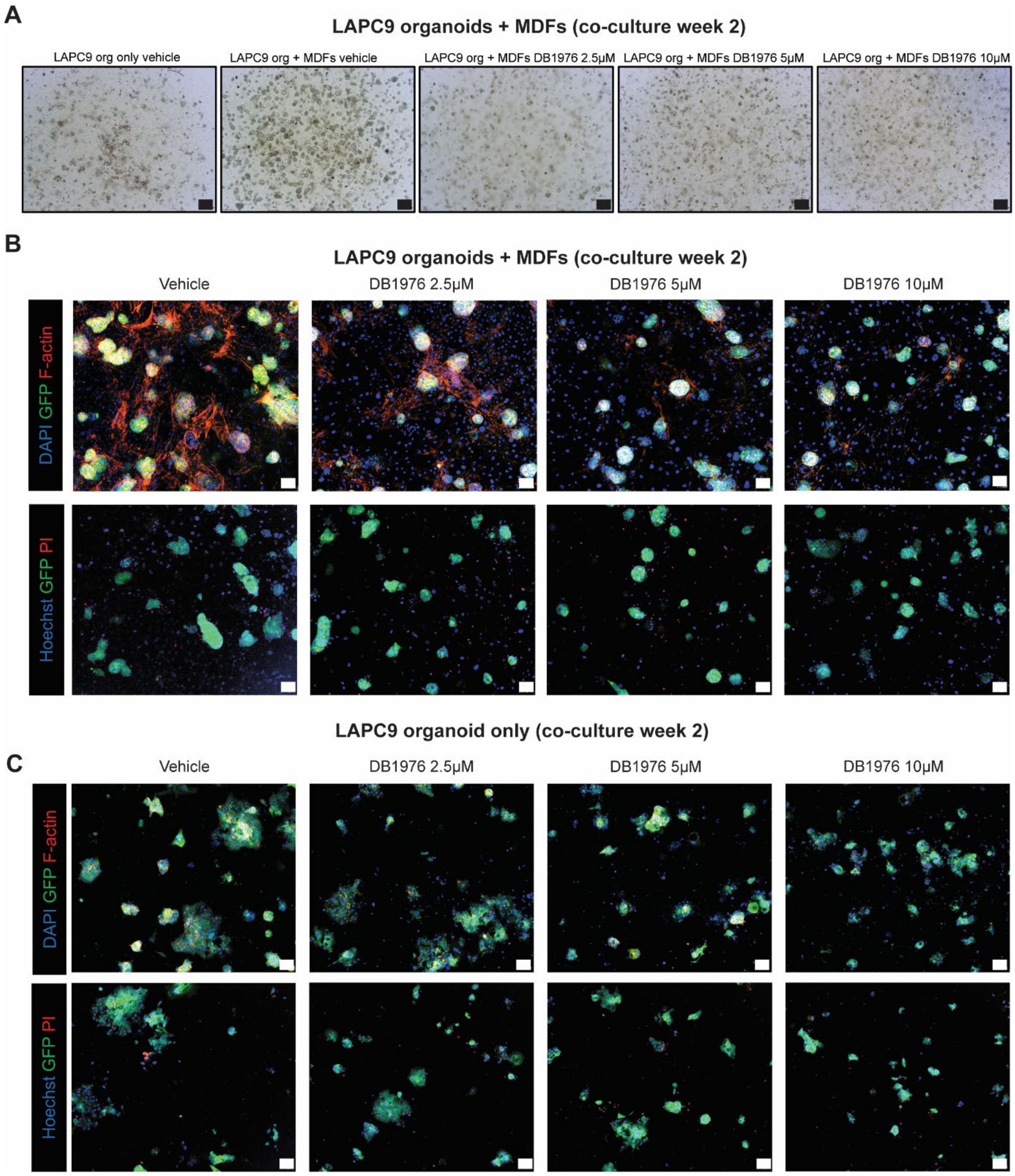

Supplementary Figure 10. Blockade of PU.1 activity inhibits the myofibroblast phenotype but does not directly impact tumor organoid and fibroblast viability. A. Brightfield images of LAPC9-GFP tumor + MDFs collagen co-cultures after 2-week DB1976 treatment at the indicated doses. Representative image of n=3 technical replicates. Scale bar: 200 µm. **B.** Phalloidin-staining (F-actin) images (top panels, formalin-fixed) and Hoechst4432 and propidium iodide (PI) staining images (bottom panels, live cells) of LAPC9-GFP tumor + MDF collagen co-cultures treated with DB1976 2.5µM, 5µM, and 10µM treatment for 2 weeks. Top panels: DAPI (blue), GFP (green), F-actin(red). Bottom panels: Hoechst3342 (blue), GFP (green), PI (red). DAPI or Hoechst3342 (blue) marks the nuclei. Representative image of n=2 technical replicates Scale bar: 20 µm. **C.** Same as in *B* but referred to LAPC9 tumor collagen monocultures.

Supplementary Figure 11

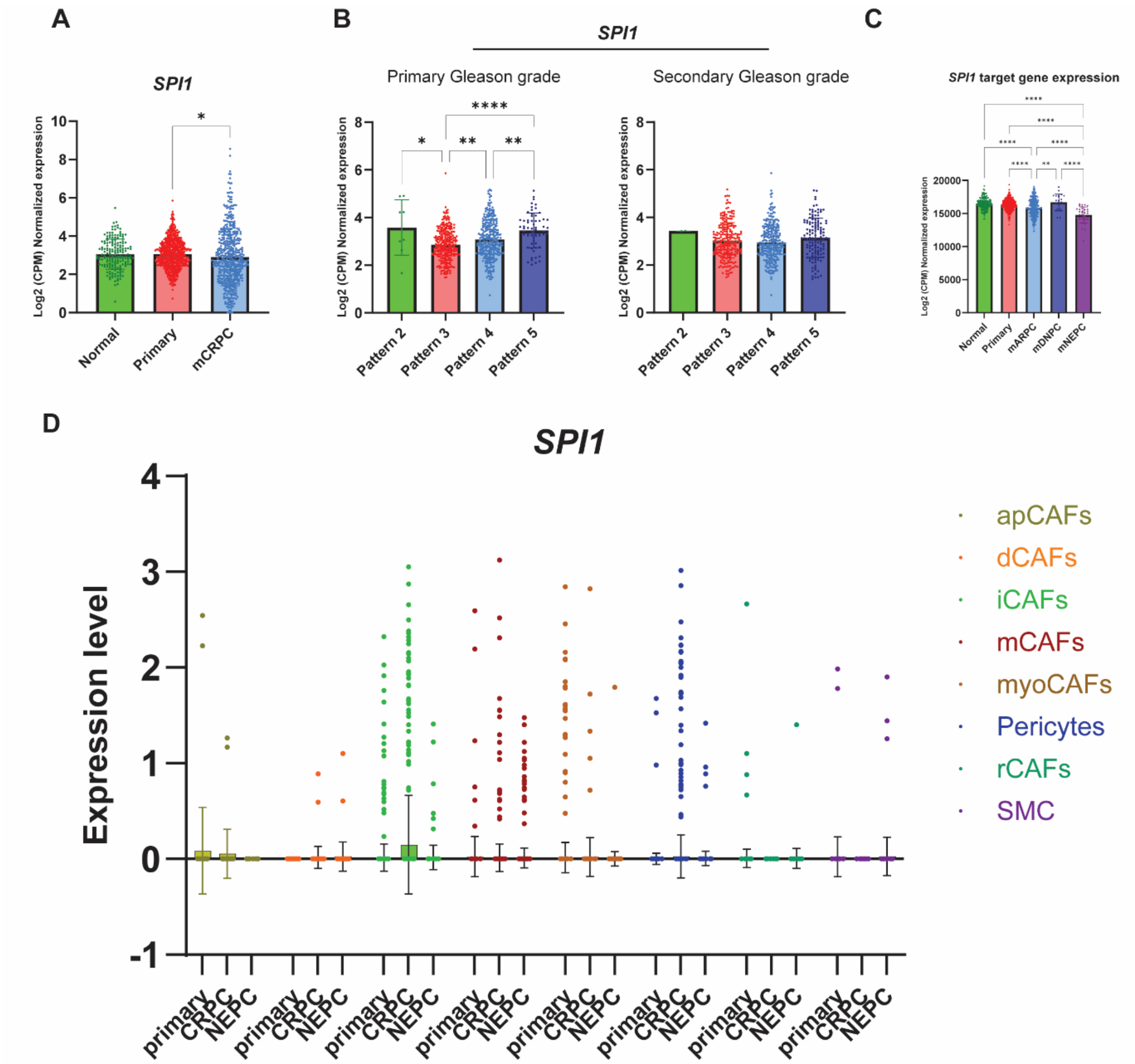

Supplementary Fig.11. PU.1 (*SPI1*)-mediated formation of a pro-fibrotic stroma is associated with CRPC progression. A. Bar plots representing *SPI1* gene expression in normal prostate, primary PCa, and metastatic CRPC (mCRPC). The logarithm of counts per million reads (log CPM) is reported on the y-axis. Mean, standard deviation, and each single measurement (dots) reported. Ordinary one-way ANOVA with Tukey's multiple comparisons, $p < 0.05$ (*), $p < 0.01$ (**), $p < 0.001$ (***), $p < 0.0001$ (****). **B.** Bar plots representing *SPI1* gene expression across PCa samples stratified according to primary (left) or secondary (right) Gleason grades. The logarithm of counts per million reads (log CPM) is reported on the y-axis. Mean, standard deviation, and each single measurement (dots) reported. Ordinary one-way ANOVA with Tukey's multiple comparisons, $p < 0.05$ (*), $p < 0.01$ (**), $p < 0.001$ (***), $p < 0.0001$ (****). **C.** Bar plots representing the expression of *SPI1* target genes (according to MR analysis, *Sup. Table 2*) across different PCa stages. Gene set score is reported on the y-axis. Ordinary one-way ANOVA with Tukey's multiple comparisons, $p < 0.05$ (*), $p < 0.01$ (**), $p < 0.001$ (***), $p < 0.0001$ (****). ARPC: AR-positive castration-resistant prostate cancer, DNPC: double-negative prostate cancer, NEPC: neuroendocrine prostate cancer. **A-C.** Data and sample stratifications are based on a cross-study comparison of multiple bulk RNA-seq datasets performed through the Prostate Cancer Atlas[2]. **D.** Bar plots showing human *SPI1* normalized gene expression level across the indicated stromal cell types comparing its expression in primary PCa, CRPC, and NEPC samples. Mean, standard deviation, and individual values (dots) reported. Another version of the plot is reported in *Fig. 6C*. Data have been obtained from the single-cell RNA sequencing Prostate Cancer Cell Atlas (PCCAT)[3]. apCAF: antigen-presenting CAF, dCAF: differentiated CAF, iCAF: inflammatory-like CAF, mCAF: matrix-associated CAF, myoCAF: myofibroblast-like CAF, rCAF: reticular CAF, SMC: smooth-muscle cells. These subpopulations were defined in[3] .

Supplementary Table 1: Tumor invasive nest analysis

PDX model	Sample	Count invasive nests	Invasive nest area (mm^2)	Total tumor area (mm^2)	Percentage of invasive nest area (%)	Analysis performed using
**LAPC9**	LAPC9 tissue sample #1 (week 2)	0	0.00	17.21	0.00	Ki67 IHC staining *
**LAPC9**	LAPC9 tissue sample #2 (week 2)	0	0.00	7.63	0.00	Ki67 IHC staining *
**LAPC9**	LAPC9 tissue sample #3 (week 3)	24	0.32	6.43	4.97	Ki67 IHC staining *
**LAPC9**	LAPC9 tissue sample #4 (week 3)	54	1.19	21.12	5.65	Ki67 IHC staining *
**LAPC9**	LAPC9 tissue sample #5 (week 5)	69	5.36	48.90	10.95	Ki67 IHC staining *
**LAPC9**	LAPC9 tissue sample #6 (week 5)	130	4.76	47.42	10.03	Ki67 IHC staining *
**LAPC9**	LAPC9 tissue sample #7	36	2.13	26.23	8.12	H&E staining
**BM18**	BM18 sample #1	not detected				H&E staining
**BM18**	BM18 sample #2	not detected				H&E staining
**PNPCa**	PNPCa sample #1	5	0.19	36.76	0.52	H&E staining
**PNPCa**	PNPCa sample #2	36	2.13	26.23	8.12	H&E staining

Supplementary Table 1: Invasive tumor nest analysis

Table reporting the count of invasive tumor nests, their area (mm2), the total tumor area (mm2), and the percentage of invasive tumor area over the total tumor area (%) for the indicated samples. The analysis has been performed on Ki67 IHC or H&E staining tissue sections.

***** = Invasive tumor nest analysis has also been performed on available H&E staining for these samples

Supplementary Table 2: *Spi1*-target genes

	LAPC9 vs BM18 p0 stroma			LAPC9 vs PNPCa p0 stroma		
**gene**	**log2 FoldChange**	**p-value**	**p-adj**	**log2 FoldChange**	**p-value**	**p-adj**
Acan	-3.602732539	4.76921e-05	0.000594637	-4.368516	4.0989e-07	1.16293e-05
Anxa1	0.754708488	9.10078e-06	0.000150572	0.779202314	7.08826e-07	1.87387e-05
Bcl2l11	0.69218315	0.001886081	0.011753088	0.508446259	0.008916461	0.048844822
Calm1	0.308051326	0.000450594	0.003784937	0.433684892	7.98347e-08	2.8191e-06
Ccr7	1.555923732	0.002617231	0.015125087	1.18124913	0.008774814	0.048266392
Cd44	1.988098752	1.18268e-20	8.29287e-18	1.546468224	1.49094e-15	2.46676e-13
Cd80	1.82138284	0.001950743	0.012059065	1.551544987	0.001147268	0.009862075
Cdk6	-1.038458684	3.78561e-09	1.92883e-07	-0.863762486	1.32917e-07	4.39822e-06
Cdkn2a	-2.195651965	8.55695e-09	3.9623e-07	-1.22833106	0.0011654	0.009973111
Csf1	0.760068893	4.25926e-05	0.000543481	0.560244337	0.000928017	0.008236922
Csf2rb	1.365804151	5.78953e-08	2.09461e-06	1.103766785	6.63998e-07	1.77126e-05
Csf2rb2	0.961560387	8.32606e-06	0.000139637	0.996865747	1.18022e-07	3.9951e-06
Cxcl9	2.374241656	4.39777e-06	8.1661e-05	3.448081872	9.11886e-13	9.12265e-11
F10	2.624378063	1.07529e-07	3.57418e-06	1.747370105	4.95463e-06	0.00010114
Fgfr1	1.085391794	2.65292e-13	4.11202e-11	0.689810764	2.18236e-07	6.7511e-06
Fgl2	-1.243792279	1.76844e-07	5.46899e-06	-2.449688104	5.06304e-29	4.65958e-26
Fn1	1.017063988	1.04492e-08	4.74889e-07	0.605200047	0.000230433	0.002646747
Gadd45a	1.422608542	1.61929e-08	6.97194e-07	0.640900732	0.002742423	0.019795182
H2-T23	0.811716518	9.2102e-06	0.000152211	0.711681017	1.3828e-05	0.000241826
Hp	2.878859401	5.14735e-07	1.33913e-05	1.89717021	0.000100624	0.001286805
Ifit3	1.377784507	0.002026834	0.012440653	1.022735241	0.009024331	0.049254843
Il1rl1	2.953800567	6.55321e-09	3.15347e-07	2.095678309	2.07772e-06	4.80289e-05
Jak1	0.788216066	3.82702e-10	2.5615e-08	0.679466017	1.57422e-09	8.68178e-08
Jak2	0.838540563	2.04465e-06	4.25246e-05	0.825906779	1.12125e-07	3.83964e-06
Lcn2	2.910393797	4.58215e-08	1.71249e-06	2.153527361	2.15946e-07	6.70844e-06
Lsp1	1.210963689	9.41636e-14	1.55793e-11	0.422538172	0.003042286	0.021332222

Mmp2	1.045342092	3.87679e-11	3.44473e-09	0.518184978	0.000353267	0.003739649
Mmp9	2.371632555	0.00024163	0.002237735	1.5207597	0.008798194	0.048340826
Myc	0.512518846	0.002464732	0.014401418	0.803848972	2.328e-07	7.112e-06
Nfkbia	0.44742151	0.001128867	0.007831369	0.649912453	2.7742e-07	8.31978e-06
Oas1a	1.633191681	6.81104e-05	0.000798817	1.450189462	5.25359e-05	0.000740988
Pebp1	-0.406234935	9.76301e-05	0.001081718	-0.661376251	7.13122e-13	7.29217e-11
Ptpn13	-0.823465767	0.012194075	0.048450554	-0.972261708	0.001165618	0.009973111
Ptx3	1.5886146	0.001180037	0.008108284	1.459484581	0.0013393	0.011167154
Reg1	4.394116119	4.33045e-12	4.7234e-10	1.716327678	0.000758871	0.006983982
Rps15	-0.635673992	2.22572e-15	5.28608e-13	-0.437037765	3.77185e-09	1.90861e-07
Samhd1	1.090433413	2.10472e-06	4.33454e-05	0.923753903	8.12478e-06	0.000153578
Scarb1	-1.136332119	1.22459e-07	3.98941e-06	-1.115704532	2.21781e-08	9.25133e-07
Slc31a1	1.077219623	2.50927e-09	1.36427e-07	0.393041668	0.005164829	0.031874312
Stat3	0.496217083	0.000104599	0.001140057	0.379437705	0.000931323	0.008261283
Syk	1.473130149	9.83762e-06	0.000160776	0.762075407	0.008528723	0.047301487
Tbxa2r	-1.356890837	1.28582e-05	0.000200569	-1.263777911	1.22842e-05	0.00021846
Tec	-2.32056741	1.16397e-09	6.99665e-08	-1.606937406	2.04417e-05	0.000338476
Tgfbr2	0.775350199	4.19132e-07	1.13035e-05	0.504294903	0.000286471	0.003166883
Timp1	1.818501151	6.58325e-07	1.63747e-05	1.206395551	0.000397031	0.004099772
Tnfaip3	1.058630318	1.4811e-07	4.70025e-06	0.759979196	3.45393e-05	0.000523732
Trim63	-4.130165128	1.02075e-07	3.4395e-06	-4.323430938	1.22696e-08	5.43557e-07
Tspo	0.665041028	0.001542893	0.010061603	0.601096943	0.001880873	0.014724006
Ugdh	1.296979134	4.48202e-11	3.9052e-09	0.945423044	6.13374e-08	2.23928e-06
Utrn	-0.7765656	2.55181e-08	1.02946e-06	-0.421664757	0.001260809	0.010617591
Vim	0.511337325	1.85093e-06	3.89355e-05	0.44886025	5.99465e-06	0.000119771

Supplementary Table 2: *Spi1*-target genes

Table reporting the list of *Spi1* target genes based on the master regulator (MR) analysis performed on LAPC9 vs BM18 and LAPC9 vs PNPCa p0 stroma. Log2 fold change, p-value, and adjusted p-value for the LAPC9 vs BM18 p0 stroma and LAPC9 vs PNPCa p0 stroma comparisons are reported. Genes highlighted in bold are known ECM/CAF-related genes.

Supplementary Table 3: Antibodies used

Primary antibodies	Application	Dilution	Company	Catalog #
α-SMA	IF	1 to 250	Invitrogen	PA5-18292
α-SMA	IF, WB	1 to 250 (IF),<br>1 to 1000 (WB)	Sigma Aldrich	A2547
Actin (Phalloidin-iFluor555 Reagent)	IF	1 to 500	Abcam	ab176756
AR	IF	1 to 50	Santa Cruz	sc-7305
CD146	IF	1 to 50	Abcam	ab75769
CD45	IF, IHC	1 to 200 (IF, IHC)	Thermo Fisher	14-0451-82
CD45-FITC	FACS	1 to 50	Miltenyi	130-110-796
Collagen type I	IF	1 to 500	Southern BiOTECH	1310-01
Cyclophilin A	IF	1 to 500	BioConcept	51418S
E-Cadherin	IF	1 to 200	R&D Systems	AF648
EpCAM (CD326)-APC-Fire750	FACS	1 to 20	Biolegend	324234
FSP1(S100A4)	IF, IHC	1 to 1000 (IHC)	Proteintech	66489-1-Ig
HLA Class 1 ABC	IF	1 to 100	Abcam	ab70328
Ki67	IF	1 to 200	Gene Tex	GTX16667
Ki67	IHC	1 to 200	DAKO Agilent	M7240
PU.1	IF, IHC, WB	1 to 200 (IHC),<br>1 to 400 (IF),<br>1 to 1000 (WB)	Cell Signaling	2258T
Tenascin C	IF, WB	1 to 50 (IF)<br>1 to 1000 (WB)	R&D Systems	MAB2138-SP
**Secondary antibodies**	**Application**	**Dilution**	**Company**	**Catalog #**
AlexaFluor 488 donkey anti-mouse	IF	1 to 250	life technolgoies	A-21202
AlexaFluor 488 donkey anti-rabbit	IF	1 to 250	life technolgoies	A21206

AlexaFluor 555 donkey anti-mouse	IF	1 to 250	life technolgoies	A31570
anti Rat HRP	WB	1 to 1000	Thermo Fisher	31470
anti-Rabbit AF647, donkey	IF	1 to 250	Abcam	ab150075
Anti-Rabbit IgG, HRP-Linked Ab	WB	1 to 2000	Cell signaling	7074
Donkey anti-Goat IgG (H+L)Alexa Fluor™ Plus 405	IF	1 to 250	ThermoFisher	A48259
Donkey anti-mouse IgG (H+L) Secondary Antibody, HRP	WB	1 to 2000	ThermoFisher	A16011
Donkey anti-Rabbit IgG (H+L)Alexa Fluor™ Plus 405	IF	1 to 250	ThermoFisher	A48258
Donkey anti-Rat IgG (H+L), Alexa Fluor™ Plus 555	IF	1 to 250	ThermoFisher	A48270

Supplementary Table 4: Mouse-specific gene primers

Gene	Forward primer	Reverse-primer
Acta2	AGCCATCTTTCATTGGGATGG	CCCCTGACAGGACGTTGTTA
ActB	CACTGTCGAGTCGCGTCC	TCATCCATGGCGAACTGGTG
Cd11b	CCATGACCTTCCAAGAGAATGC	ACCGGCTTGTGCTGTAGTC
Cd45	ATATCGCGGTGTAAAACTCGTC	TAGGCTTAGGCGTTTCTGGAA
Col1a1	ACGCCATCAAGGTCTACTGC	TTCCGTACTCGAACGGGAATC
Col2a1	GGGTCACAGAGGTTACCCAG	ACCAGGGGAACCACTCTCAC
Col3a1	CTGTAACATGGAAACTGGGGAAA	CCATAGCTGAACTGAAAACCACC
Col5a1	CTTCGCCGCTACTCCTGTTC	CCCTGAGGGCAAATTGTGAAAA
Csf2	TCGTCTCTAACGAGTTCTCCTT	CGTAGACCCTGCTCGAATATCT
Gli1	CTCGACCTGCAAACCGTAATC	TCCTAAAGAAGGGCTCATGGTA
Gli2	CAACGCCTACTCTCCCAGAC	GAGCCTTGATGTACTGTACCAC
Hprt	GTTGGGCTTACCTCACTGCT	TCATCGCTAATCACGACGCT
Il1a	CGAAGACTACAGTTCTGCCATT	GACGTTTCAGAGGTTCTCAGAG
Il1b	GCAACTGTTCCTGAACTCAACT	ATCTTTTGGGGTCCGTCAACT
Il6	TCTATACCACTTCACAAGTCGGA	GAATTGCCATTGCACAACTCTTT
Itgav	GGTCGCCTATCTTCGGGATG	TGAACTGGTTCAGGATGGGC
Mmp3	TTAAAGACAGGCACTTTTGGCG	CCCTCGTATAGCCCAGAACT
Mmp9	GGACCCGAAGCGGACATTG	CGTCGTCGAAATGGGCATCT
Smo	GAGCGTAGCTTCCGGGACTA	CTGGGCCGATTCTTGATCTCA
Spi1	GAGGTGTCTGATGGAGAAGCTG	ACCCACCAGATGCTGTCCTTCA
Thbs1	GGGGAGATAACGGTGTGTTTG	CGGGGATCAGGTTGGCATT
Tnc	GTAACGGTGGTGGATTCTGGGA	TCCGGTTCAGCTTCTGTAATGGTC
Tnf	CCCTCACACTCAGATCATCTTCT	GCTACGACGTGGGCTACAG

Uncropped Western Blot represented in Sup. Fig. 6D

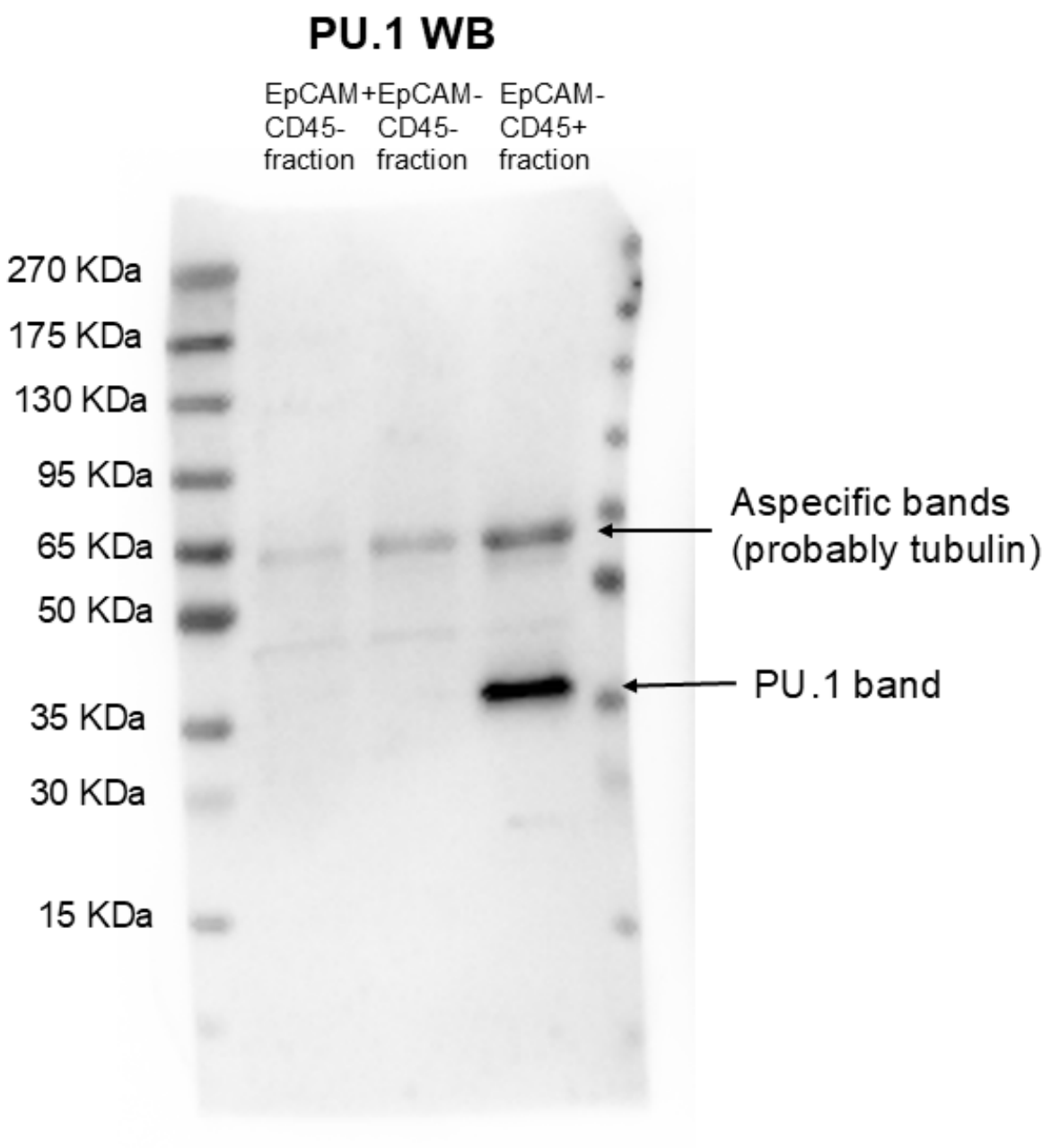

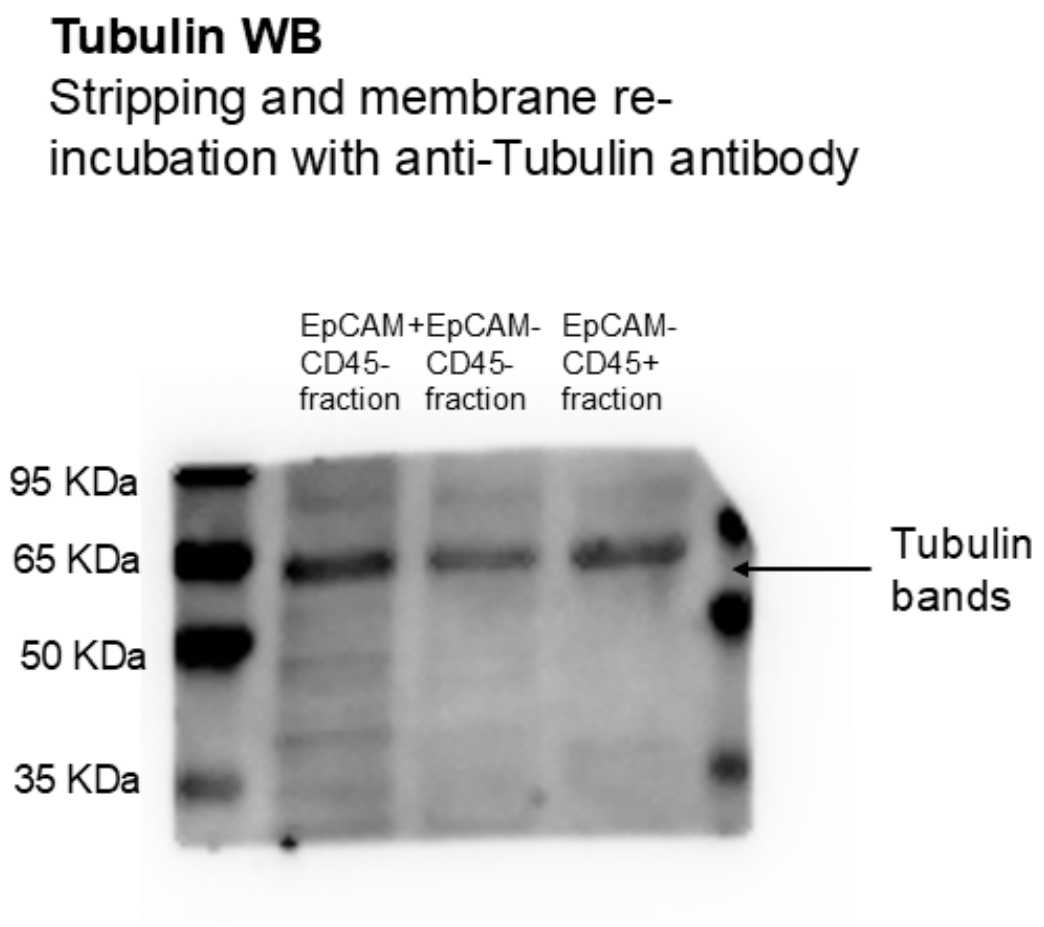

Marker: Broad Multi Color Pre-Stained Protein Standard (Gene Script, M00624-250)

References

1. Karkampouna, S. *et al.* Stroma Transcriptomic and Proteomic Profile of Prostate Cancer Metastasis Xenograft Models Reveals Prognostic Value of Stroma Signatures. *Cancers 2020, Vol. 12, Page 3786* 12, 3786 (2020).
2. ProstateCancerAtlas. https://prostatecanceratlas.org/app/profiler.
3. PCCAT. https://pccat.net/.
