# Supplementary material for: PU.1 is a mediator of the reactive stromal response associated with castration-resistant prostate cancer
Source: Commun Biol. 2026 May 29;9:1174. doi: 10.1038/s42003-026-10297-7 (PMC13547233; doi:10.1038/s42003-026-10297-7)
Supplement: Supplementary file 3 [file 42003_2026_10297_MOESM3_ESM.pdf]

Description of Additional Supplementary Files

File name: Supplementary Data

Description: The numerical data sources for all the plots generated are reported in the Supplementary Data file
