# Supplementary material for: PU.1 is a mediator of the reactive stromal response associated with castration-resistant prostate cancer
Source: Commun Biol. 2026 May 29;9:1174. doi: 10.1038/s42003-026-10297-7 (PMC13547233; doi:10.1038/s42003-026-10297-7)
Supplement: Supplementary file 4 [file 42003_2026_10297_MOESM5_ESM.pdf]

nature portfolio

Corresponding author(s): *Kruithof-de Julio & Karkampouna*

Last updated by author(s): *2026-04-24*

Reporting Summary

Nature Portfolio wishes to improve the reproducibility of the work that we publish. This form provides structure for consistency and transparency in reporting. For further information on Nature Portfolio policies, see our Editorial Policies and the Editorial Policy Checklist.

Statistics

For all statistical analyses, confirm that the following items are present in the figure legend, table legend, main text, or Methods section.

n/a	Confirmed	
☐	☒	The exact sample size (*n*) for each experimental group/condition, given as a discrete number and unit of measurement
☐	☒	A statement on whether measurements were taken from distinct samples or whether the same sample was measured repeatedly
☐	☒	The statistical test(s) used AND whether they are one- or two-sided<br>*Only common tests should be described solely by name; describe more complex techniques in the Methods section.*
☒	☐	A description of all covariates tested
☐	☒	A description of any assumptions or corrections, such as tests of normality and adjustment for multiple comparisons
☐	☒	A full description of the statistical parameters including central tendency (e.g. means) or other basic estimates (e.g. regression coefficient) AND variation (e.g. standard deviation) or associated estimates of uncertainty (e.g. confidence intervals)
☐	☒	For null hypothesis testing, the test statistic (e.g. *F*, *t*, *r*) with confidence intervals, effect sizes, degrees of freedom and *P* value noted<br>*Give P values as exact values whenever suitable.*
☒	☐	For Bayesian analysis, information on the choice of priors and Markov chain Monte Carlo settings
☒	☐	For hierarchical and complex designs, identification of the appropriate level for tests and full reporting of outcomes
☐	☒	Estimates of effect sizes (e.g. Cohen's *d*, Pearson's *r*), indicating how they were calculated

Our web collection on statistics for biologists contains articles on many of the points above.

Software and code

Policy information about availability of computer code

Data collection	Quantitative RT-PCR data was collected using the QuantStudio Design and analysis software (v 1.3.1). Flow cytometry data were collected using FACS Diva (v 8.0). RNASeq data were obtained through MercuriusTM BRB-Seq at Alithea Genomics. For numerical plot representation, the R programming language (v4.4.2) and the GraphPad Prism software (v10.4.1) were used. For descriptive schemes and figures BioRender.com was used.
Data analysis	All software used was either open source or commercially available.<br>Double-IHC quantification analyses were performed with the Visiopharm software (v2023.09x64 Hørsholm, Denmark).<br>For RNA-seq data analysis, quality check was performed using fastQC (v0.11.7). Sequencing reads were aligned to either the human genome (GRCh38, Ensembl 102) or the mouse genome (GRCm38, Ensembl 102) with STAR361 (v2.7.10a). Downstream analyses were conducted using R (v4.4.2). Deduplicated unique molecular identifiers (umi) count matrices were used to create the initial DESeq2 object68 (v1.46.0). Differential expression analysis was performed using DESeq2 with the Wald method. Enrichment analysis was conducted using the clusterProfiler package69 (v4.14.4) with the compareCluster function and the "enrichGO" method using the gene ontology biological processing gene sets. MR inference was performed using the decoupleR package40 (v2.12.0) with the decouple function, utilizing the collecTRI mouse regulon70. Subsequently, the different scoring methods were combined using the run_consensus function from decoupleR.

For manuscripts utilizing custom algorithms or software that are central to the research but not yet described in published literature, software must be made available to editors and reviewers. We strongly encourage code deposition in a community repository (e.g. GitHub). See the Nature Portfolio guidelines for submitting code & software for further information.

Data

Policy information about availability of data

All manuscripts must include a data availability statement. This statement should provide the following information, where applicable:

- Accession codes, unique identifiers, or web links for publicly available datasets
- A description of any restrictions on data availability
- For clinical datasets or third party data, please ensure that the statement adheres to our policy

The RNA sequencing data generated in this study are available through the Gene Expression Omnibus (GEO) with accession number GSE296466 (reviewer access token: ylqbumssfpmvnav). The RNA sequencing data related to Suppl.Fig. 5A is generated by a previous study and are available at EGA under EGAS00001004770.

Research involving human participants, their data, or biological material

Policy information about studies with human participants or human data. See also policy information about sex, gender (identity/presentation), and sexual orientation and race, ethnicity and racism.

Reporting on sex and gender	The tumor tissue of he PDX models (PNPCa, BM18, LAPC9) is of human origin. PDXs were generated from prostate cancer metastatic specimens (soft tissue and bone) of Caucasian men.<br>Reference studies for the three PDX models:<br>- Karkampouna, S., La Manna, F., Benjak, A. et al. Patient-derived xenografts and organoids model therapy response in prostate cancer. Nat Commun 12, 1117 (2021).<br>- McCulloch, D. R., Opeskin, K., Thompson, E. W. & Williams, E. D. BM18: A novel androgen-dependent human prostate cancer xenograft model derived from a bone metastasis. Prostate 65, 35–43 (2005).<br>- Craft, N. et al. Evidence for clonal outgrowth of androgen-independent prostate cancer cells from androgen-dependent tumors through a two-step process. Cancer Res 59, 5030–5036 (1999).
Reporting on race, ethnicity, or other socially relevant groupings	*Please specify the socially constructed or socially relevant categorization variable(s) used in your manuscript and explain why they were used. Please note that such variables should not be used as proxies for other socially constructed/relevant variables (for example, race or ethnicity should not be used as a proxy for socioeconomic status).<br>Provide clear definitions of the relevant terms used, how they were provided (by the participants/respondents, the researchers, or third parties), and the method(s) used to classify people into the different categories (e.g. self-report, census or administrative data, social media data, etc.)<br>Please provide details about how you controlled for confounding variables in your analyses.*
Population characteristics	*Describe the covariate-relevant population characteristics of the human research participants (e.g. age, genotypic information, past and current diagnosis and treatment categories). If you filled out the behavioural & social sciences study design questions and have nothing to add here, write "See above."*
Recruitment	*Describe how participants were recruited. Outline any potential self-selection bias or other biases that may be present and how these are likely to impact results.*
Ethics oversight	*Identify the organization(s) that approved the study protocol.*

Note that full information on the approval of the study protocol must also be provided in the manuscript.

Field-specific reporting

Please select the one below that is the best fit for your research. If you are not sure, read the appropriate sections before making your selection.

☒ Life sciences ☐ Behavioural & social sciences ☐ Ecological, evolutionary & environmental sciences

For a reference copy of the document with all sections, see nature.com/documents/nr-reporting-summary-flat.pdf

Life sciences study design

All studies must disclose on these points even when the disclosure is negative.

Sample size	For the in vivo experiments using PDX tumor tissues, N=3 mice were implanted with two tumors (one per flank), for each of the timepoints collected and for each of the distinct PDX tumors. This number of observations was sufficient to obtain groups with homogeneous mean and median tumor volumes. For histopathological analysis, n=2 whole slide tumor areas (biological replicates) were evaluated per time point.
Data exclusions	Tumor samples used for RNA isolation were omitted due to low RNA amount. Tumor samples for the week4 time points were excluded after quality control of the tumor histomorphology.
Replication	The replicability of the RNASeq data was checked by comparing two different batches of RNASeq data, from different passages of the PDX tumors. This also confirmed the stability of the transcriptomic profile of the PDX models used, along their in vivo serial passaging in time. RNASeq was performed in biological replicates and yielded homogeneous datapoint distributions. For all in vitro experiments, data were collected using a three to five technical replicates. For the in vitro organoid drug screening, replicates

were distributed across different plates to account for assay variability. All attempts to replicate in vivo and in vitro studies were successful.

Randomization: Mice included in the in vivo experiment were randomized after PDX implantation into time point groups. Group allocation was performed randomly after PDX implantation, taking into account that the body weight distribution in different groups was comparable, and that the group participants were not all belonging to the same cage but distributed in different cages.

Blinding: For the in vivo experiment, tumor growth assessment was done in single-blind mode. For downstream histological analysis and histopathological assessment of those tumors, the samples were coded. Expert pathologist evaluated in a blinded manner the coded samples. The sample description was revealed after the quantifications were performed by the pathologist.

Reporting for specific materials, systems and methods

We require information from authors about some types of materials, experimental systems and methods used in many studies. Here, indicate whether each material, system or method listed is relevant to your study. If you are not sure if a list item applies to your research, read the appropriate section before selecting a response.

Materials & experimental systems

n/a	Involved in the study
☐	☒ Antibodies
☒	☐ Eukaryotic cell lines
☒	☐ Palaeontology and archaeology
☐	☒ Animals and other organisms
☒	☐ Clinical data
☒	☐ Dual use research of concern
☒	☐ Plants

Methods

n/a	Involved in the study
☒	☐ ChIP-seq
☐	☒ Flow cytometry
☒	☐ MRI-based neuroimaging

Antibodies

Antibodies used: The list of all antibodies used, catalog number and dilution is provided in Supplementary Table 2 in the manuscript.

Validation: The primary antibody concentrations and method specifications (e.g. antigen retrieval) were used based on the protocols of the antibody manufacturer. For IF experiments, isotope controls same as the primary antibody species were used as negative control.
aSMA (prod#: PA5-18292): positive control human intestinal tissue engrafted in mouse (according to manufacturer's description), tested and evaluated by pathologist in LAPC9 tumor tissue, reacts with human, mouse
aSMA (prod#:A2567): positive control TGFbeta-induced human and mouse myofibroblasts (described in Fig. 5A and suppl. Fig.7A), reacts with human, mouse, rat, chicken, frog, canin, rabbit etc.
AR: positive control human prostate tissue, reacts with mouse, rat, human
CD146: positive control human spleen tissue (according to manufacturer's description), reacts with human, mouse, rat
CD45: positive control mouse bone marrow cells (PMID: 33651379), reacts with mouse
CD45-FITC: positive control mouse splenocytes according to manufacturer's description, reacts with mouse
Collagen Type-I: positive control human fibroblasts, reacts with conformational determinants on type I collagen (reactivity with human, mouse, rat, canine, chicken, hamster etc. reported according to manufacturer's webiste). Tested and evaluated by pathologist in LAPC9 tumor tissue.
Cyclophylin A: positive control mouse stromal tissue in PDX tumors (suppl. Fig. 1A), reacts with mouse
E-cadherin: positive control human stomach, human stem cells according to manufacturer's description, reacts with human, mouse
FSP1 (S100A4): positive control human colon and lung cancer tissue according to manufacturer's description, reacts with human, mouse
HLA Class 1 ABC: human tonsil tissue, reacts with the heavy chains of human HLA class 1 A, -B, and -C.
Ki67 (prod #:GTX16667): positive control human oral carcinoma (manufacturer's description), reacts with human
Ki67 (prod#:M7240): positive control high-grade lymphoma (manufacturer's description), tested on LAPC9 tumor tissues, reacts with human
PU.1: positive control Non-Hodgkin's lymphoma tissue (manufacturer's description), reacts with human, mouse
TNC: positive control human glioblastoma/astrocytoma cell line (manufacturer's description), and in human prostate tissue (PMID: 33334054), reacts with human, mouse

Animals and other research organisms

Policy information about studies involving animals; ARRIVE guidelines recommended for reporting animal research, and Sex and Gender in Research

Laboratory animals: CB17-SCID mice (origin: Charles River) were used for PDX tumor tissue maintenance and in vivo experiment (described in Figure 2)

Wild animals: The study did not involve wild animals

Reporting on sex: Male mice

Field-collected samples	This study did not collect field-originating samples.
Ethics oversight	Ethical approval for the animal experiments was obtained by the Cantonal Veterinary Ethical committee, Bern Switzerland (animal license BE 67/20 and BE103/23).

Note that full information on the approval of the study protocol must also be provided in the manuscript.

Plants

Seed stocks	*Report on the source of all seed stocks or other plant material used. If applicable, state the seed stock centre and catalogue number. If plant specimens were collected from the field, describe the collection location, date and sampling procedures.*
Novel plant genotypes	*Describe the methods by which all novel plant genotypes were produced. This includes those generated by transgenic approaches, gene editing, chemical/radiation-based mutagenesis and hybridization. For transgenic lines, describe the transformation method, the number of independent lines analyzed and the generation upon which experiments were performed. For gene-edited lines, describe the editor used, the endogenous sequence targeted for editing, the targeting guide RNA sequence (if applicable) and how the editor was applied.*
Authentication	*Describe any authentication procedures for each seed stock used or novel genotype generated. Describe any experiments used to assess the effect of a mutation and, where applicable, how potential secondary effects (e.g. second site T-DNA insertions, mosiacism, off-target gene editing) were examined.*

Flow Cytometry

Plots

Confirm that:

☒ The axis labels state the marker and fluorochrome used (e.g. CD4-FITC).

☒ The axis scales are clearly visible. Include numbers along axes only for bottom left plot of group (a 'group' is an analysis of identical markers).

☒ All plots are contour plots with outliers or pseudocolor plots.

☒ A numerical value for number of cells or percentage (with statistics) is provided.

Methodology

Sample preparation	Tumor tissue/co-cultures were digested into single cells, stained, and flow cytometry was applied (described in Material and Method Section)
Instrument	BD FACS SORP LSR II, the MoFlo ASTRIOS Eq, BD FACSDiscover S8 with CellView
Software	FlowJo (BD Biosciences)
Cell population abundance	Abundances of cell populations are reported as percentage number in the histogram plots (Fig. 3J and Suppl. Fig.4B)
Gating strategy	Gating strategy for flow cytometry experiments are described in Suppl. Fig. 4B and Suppl. Fig. 6B

☒ Tick this box to confirm that a figure exemplifying the gating strategy is provided in the Supplementary Information.
